# Linker and N-Terminal Domain Engineering of Pyrrolysyl-tRNA Synthetase for Substrate Range Shifting and Activity Enhancement

**DOI:** 10.3389/fbioe.2020.00235

**Published:** 2020-04-07

**Authors:** Han-Kai Jiang, Man-Nee Lee, Jo-Chu Tsou, Kuan-Wen Chang, Hsueh-Wei Tseng, Kuang-Po Chen, Yaw-Kuen Li, Yane-Shih Wang

**Affiliations:** ^1^Institute of Biological Chemistry, Academia Sinica, Taipei, Taiwan; ^2^Chemical Biology and Molecular Biophysics Program, Taiwan International Graduate Program, Academia Sinica, Taipei, Taiwan; ^3^Department of Chemistry, National Tsing Hua University, Hsinchu, Taiwan; ^4^Department of Applied Chemistry, National Chiao Tung University, Hsinchu, Taiwan; ^5^Institute of Biochemical Sciences, National Taiwan University, Taipei, Taiwan

**Keywords:** non-canonical amino acids, pyrrolysyl-tRNA synthetase, linker engineering, amber codon suppression, tRNA binding domain

## Abstract

The *Methanosarcina mazei* pyrrolysyl-tRNA synthetase (PylRS)⋅tRNA^Pyl^ pair can be used to incorporate non-canonical amino acids (ncAAs) into proteins at installed amber stop codons. Although engineering of the PylRS active site generates diverse binding pockets, the substrate ranges are found similar in charging lysine and phenylalanine analogs. To expand the diversity of the ncAA side chains that can be incorporated *via* the PylRS⋅tRNA^Pyl^ pair, exploring remote interactions beyond the active site is an emerging approach in expanding the genetic code research. In this work, remote interactions between tRNA^Pyl^, the tRNA binding domain of PylRS, and/or an introduced non-structured linker between the N- and C-terminus of PylRS were studied. The substrate range of the PylRS⋅tRNA^Pyl^ pair was visualized by producing *sfGFP-UAG* gene products, which also indicated amber suppression efficiencies and substrate specificity. The unstructured loop linking the N-terminal and C-terminal domains (CTDs) of PylRS has been suggested to regulate the interaction between PylRS and tRNA^Pyl^. In exploring the detailed role of the loop region, different lengths of the linker were inserted into the junction between the N-terminal and the C-terminal domains of PylRS to unearth the impact on remote effects. Our findings suggest that the insertion of a moderate-length linker tunes the interface between PylRS and tRNA^Pyl^ and subsequently leads to improved suppression efficiencies. The suppression activity and the substrate specificity of PylRS were altered by introducing three mutations at or near the N-terminal domain of PylRS (N-PylRS). Using a N-PylRS⋅tRNA^Pyl^ pair, three ncAA substrates, two *S*-benzyl cysteine and a histidine analog, were incorporated into the protein site specifically.

## Introduction

Expanding the genetic code is a pragmatic approach to incorporate over 200 different kinds of non-canonical amino acids (ncAAs) into proteins *in vivo* (Vargas-Rodriguez et al., 2018). This technology employs a bioorthogonal aminoacyl-tRNA synthetase (AARS)⋅tRNA pair to decode nonsense or rare codons in living systems genetically (O’Donoghue et al., 2012). One of most used AARS⋅tRNA systems for genetic code expansion is the pyrrolysyl-tRNA synthetase (PylRS)⋅tRNA^Pyl^ derived from the archaea *Methanosarcina barkeri* (*Mb*) or *Methanosarcina mazei* (*Mm*) or the eubacteria *Desulfitobacterium hafniense* (*Dh*) (Herring et al., 2007; Neumann et al., 2008; Yanagisawa et al., 2008). The PylRS⋅tRNA^Pyl^ pair has a wide range of bio-orthogonality in different species from prokaryotes to eukaryotes (Mukai et al., 2008; Neumann et al., 2008; Han et al., 2017). The *Mm*PylRS⋅*Mm*tRNA^Pyl^ (PylRS⋅tRNA^Pyl^) pair was naturally evolved for amber TAG codon recoding with slow enzyme kinetic properties (Guo et al., 2014). Uniquely, PylRS harbors a sophisticated and dynamic active site for recognizing pyrrolysine, a lysine analog modified with a 4-methylpyrroline-ring through an *N*^ε^-amide bond linkage (Hao et al., 2002). Various evolved PylRS⋅tRNA^Pyl^ pairs for incorporating lysine and phenylalanine analogs have been reported through directed-evolution and rational design approaches (Mukai et al., 2008; Neumann et al., 2008; Wang Y.S. et al., 2012; Wang et al., 2010; Guo et al., 2014). The diverse substrate scope and co-crystal structures of ncAA substrate-PylRS mutants reveal that the substrate can have multiple binding modes and a slower feature in enzymatic kinetic studies (Kavran et al., 2007; Nozawa et al., 2009). The directed evolution of full-length PylRS has been studied. However, a systematic investigation of interactions between the C-terminal domain (CTD) and the N-terminal domain (NTD) of the protein and the cognate tRNA, as well as the impact of those interactions of the dynamic substrate range, remains unexplored.

The co-crystal structure of *Mm*PylRS CTD with pyrrolysine revealed a distinct binding mode for the amino acid, which is deeply embedded into the hydrophobic pocket of the catalytic domain. The CTD of PylRS contains the binding domain that harbors tRNA^Pyl^ and ATP (Nozawa et al., 2009). The interplay between tRNA^Pyl^ and PylRS plays a key role in affecting enzymatic activity and substrate scope. The G1:C72 base pair and discriminator G73, which are located on the acceptor arm of tRNA^Pyl^, are thought to be required for PylRS recognition (Herring et al., 2007). Mutations in the D loop, T loop, or variable loop of tRNA^Pyl^ spatially perturb its shape, which in turn diminishes the activity of PylRS. The suppression efficiency is slightly influenced by a change in the anticodon loop compared to the D or the T loop, suggesting the inessential element for PylRS recognition (Ambrogelly et al., 2007).

**FIGURE 1:**
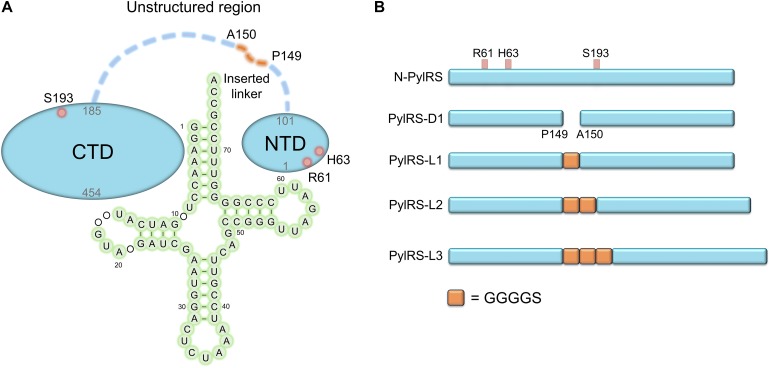
Protein designs of evolved *Mm*PylRS and their linker engineering. **(A)** Illustration of the interaction between *Mm*PylRS and tRNA^Pyl^. R61K/H63Y/S193R mutations (labeled in open red circles) are distributed on the tRNA binding domain of *Mm*PylRS NTD or CTD (labeled as filled blue circle). The detailed interactions from X-ray crystal structures in PylRS-NTD/tRNA (Suzuki et al., 2017) and PylRS-CTD/tRNA (Nozawa et al., 2009) are indicated in Figures 2, 3. tRNA 2D topology and sequence are labeled in green filled circles. The orange dashed line denotes the inserted linker between P149 and A150. **(B)** Engineered *Mm*PylRS constructs used in this work. N-PylRS represents *Mm*PylRS with R61K/H63Y/S193R mutations; PylRS-D1 represents *Mm*PylRS with insertion of the spacer containing the stop and the start codons between P149 and A150; PylRS-L1 indicates the insertion of SGGGGS linker between P149 and A150; PylRS-L2 for S(GGGGS)_2_ linker insertion; PylRS-L3 for S(GGGGS)_3_ linker insertion.

The anticodon loop and acceptor arm of the tRNA are fundamental and essential elements for endogenous AARSs recognition (Schimmel et al., 1993; O’Donoghue and Luthey-Schulten, 2003). As opposed to this binding mode, PylRS recognizes its cognate tRNA^Pyl^ distinctly. This characteristic helps PylRS to selectively distinguish tRNA^Pyl^ in the translational system. The T loop and the variable loop of tRNA^Pyl^ have hydrophilic interactions with the PylRS-NTD. The tRNA^Pyl^ is oppositely embraced by the NTD and the CTD of PylRS, which is in connection with the postulated flexible loops (Suzuki et al., 2017; Figure 1A). This compatible binding mode dynamically controls the interaction between PylRS and tRNA^Pyl^.

**SCHEME 1:**
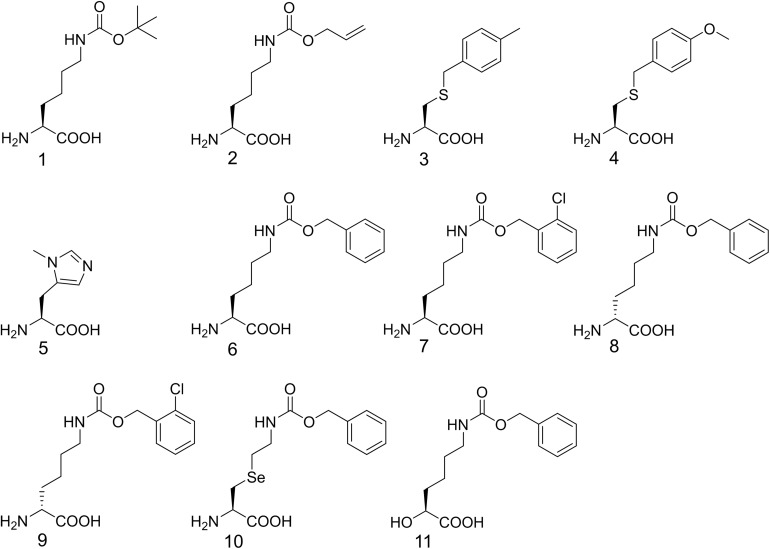
Chemical structures of the non-canonical amino acids described in this study. ncAA **1**: *N*^ε^-(*tert*-butoxycarbonyl)-L-lysine (BocK); **2**: *N*^ε^- (alloyloxycarbonyl)-L-lysine (AlloK); **3**: *S*-(4-methylbenzyl)-L-cysteine (MbzlC); **4**: *S*-(4-methoxylbenzyl)-L-cysteine (MeObzlC); **5**: *N*-π-methyl-L-histidine (3MeH); **6**: *N*^ε^-(carbobenzyloxy)-L-lysine (CbzK); **7**: *N*^ε^-(2-chlorocarbobenzyloxy)-L-lysine (ClCbzK); **8**: *N*^ε^-(carbobenzyloxy)-D-lysine (DCbzK); **9**: *N*^ε^-(2-chlorocarbobenzyloxy)- D-lysine (DClCbzK); **10**: (*R*)-2-amino-3-(2-benzyloxycarbonylaminoethylselanyl)propanoic acid (SeCbzK); **11**: (*S*)-6-(benzyloxycarbonylamino)-2-hydroxygexanoic acid (CbzKOH).

Moreover, the truncation of PylRS NTD significantly precludes the binding specificity of PylRS by making it incapable to charge tRNA^Pyl^ (Jiang and Krzycki, 2012). The mutations on NTD of the evolved chimeric PylRS (chPylRS) have played a crucial role in tuning the binding of tRNA^Pyl^ (Suzuki et al., 2017). The previous study of class II AARS seryl-tRNA synthetase (SerRS) showed that the R76 and the R94 of the NTD of *Mb*SerRS were curial for tRNA recognition, and the alanine mutations of both had abolished the amber suppression efficiency (Jaric et al., 2009). N-terminal Add-1 domain from an exceptional class I AARS arginyl-tRNA synthetase (ArgRS) of *Saccharomyces cerevisiae* was found to bind the D loop of tRNA^Arg^, which is a rare spatial arrangement among class I AARSs but similar to *Mm*PylRS and *Mb*SerRS (Cavarelli et al., 1998). The length of NTD extensively affects the orientation of tRNA^Arg^ by the positional change of ArgRS (Shimada et al., 2001). Using directed evolution, certain mutations generated on PylRS NTD accompanied by variations surrounding the catalytic site also led to increased suppression efficiencies as well as substrate specificity (Mukai et al., 2015). Evolved PylRS harboring three mutations on NTD was found to enhance the suppression efficiency in charging BocK (**1**, Scheme 1) (Sharma et al., 2018). The above studies support the idea that the mutations on the NTD might influence the binding mode and the activity of PylRS.

Here we report the generation of PylRS variants with mutations beyond the catalytic site, which has allowed us to further decipher the interaction between PylRS and tRNA^Pyl^. Fast screening of a ncAAs library using an amber-codon-installed *superfolder green fluorescent protein* (*sfGFP*) gene allowed us to determine the substrate range and suppression efficiency and, in turn, fine-tuning of the interaction between PylRS and tRNA^Pyl^. We found that extending the length of a linker between the NTD and the CTD of PylRS further probed the management of flexible loop and their effectiveness in ncAA incorporation yield.

## Materials and Methods

### General Strains and Plasmid Constructions

Natural amino acids and ncAAs were purchased from Chem Impex Inc (Wood Dale, IL, United States). The ncAA SeCbzK (**10**) (Wang Z.U. et al., 2012) and CbzKOH (**11**) (Li et al., 2012; Wang, 2012) (Scheme 1) were synthesized by the reported methods. PCR was performed using the KOD hot start polymerase kit (Merck). Oligonucleotide synthesis and DNA sequencing were done by Genomics Inc. (Taipei, Taiwan). The primer sequences are listed in the Supplementary Materials. Construction of the pET-*pylT-sfGFP-TAG2* and the pET-*pylT-sfGFP-TAG27* plasmids followed general cloning protocols. The *sfGFP-TAG2* gene was amplified by PCR from pET-*pylT-sfGFP* (Guo et al., 2014) with the primers sfGFP-TAG2-*Nde*I-F and sfGFP-*Sac*I-R2. The *sfGFP-TAG27* was generated by overlap extension PCR with two fragments (fragment 1: primer sfGFP-*Nde*I-F1 and sfGFP-TAG27-R; fragment 2: sfGFP-TAG27-F and sfGFP-*Sac*I-R2). The overlapped *sfGFP* gene products and pET-*pylT* plasmid were then double-digested with restriction enzyme *Nde*I and *Sac*I, and then T4 DNA ligase was used for DNA ligation.

In the construction of the plasmid pCDF-*N-PylRS* variants, the *PylRS-R61K/H63Y/S193R* (*N-PylRS*) gene was generated by overlap extension PCR with three fragments using the template pCDF-*PylRS*: fragment 1: PylRS-*Nco*I-F and PylRS-R61K/H63Y-R; fragment 2: PylRS-R61K/H63Y-F and PylRS-S193R-R; fragment 3: PylRS-S193R-F and PylRS-*Bam*HI-R. In preparing pCDF-*PylRS-R61K*, pCDF-*PylRS-H63Y*, and pCDF-*PylRS-S193R*, the same procedures were performed with the following primers to produce *PylRS-R61K*, *PylRS- H63Y*, and *PylRS-S193R* genes with pCDF-*PylRS* template and following the overlap extension PCR; *PylRS-R61K* gene: fragment 1, PylRS-*Nco*I-F and PylRS-R61K-R; fragment 2, PylRS-R61K-F and PylRS-*Bam*HI-R; *PylRS-H63Y* gene: fragment 1, PylRS-*Nco*I-F and PylRS-H63Y-R; fragment 2, PylRS-H63Y-F and PylRS-*Bam*HI-R; *PylRS-S193R* gene: fragment 1, PylRS-*Nco*I-F and PylRS-S193R-R; fragment 2, PylRS-S193R-F and PylRS-*Bam*HI-R. In preparing pCDF-*PylRS-ND*, the *PylRS-ND* gene fragment was generated by PCR with two primers, PylRS-*Nco*I-F and PylRS-P149-*Bam*HI-R, and pCDF-*PylRS* was used as a template. In the preparation of pCDF-*PylRS-D1*, the spacer sequence (TGAAAAAAGCGATG), including the TGA stop codon and the ATG start codon, was installed between positions P149 and A150 of the *PylRS* gene. The *PylRS-D1* gene fragment was generated by overlap extension PCR with two fragments using pCDF-*PylRS* as a template. Fragment 1 was generated by the primers PylRS-*Nco*I-F and PylRS-P149SSS-R1; fragment 2 was generated by the primers PylRS-P149SSS-F2 and PylRS-*Bam*HI-R. Different lengths of linker were installed in PylRS variants between sposition 149 and 150. In preparing pCDF-*PylRS-L1*, pCDF-*PylRS-L2*, and pCDF-*PylRS-L3* (Figure 1B), the same procedures were performed by the following primers to produce *PylRS-L1*, *PylRS-L2*, and *PylRS-L3* genes with pCDF-*PylRS* template and following overlap extension PCR: *PylRS-L1* gene, PylRS-*Nco*I-F, and Linker-1XG4S-R1, Linker-1XG4S-F2, and PylRS-*Bam*HI-R; *PylRS-L2* gene: fragment 1, PylRS-*Nco*I-F and Linker-2XG4S-R1; fragment 2, Linker-2XG4S-F2 and Linker-2XG4S-R2; fragment 3, Linker-2XG4S-F3 and PylRS-*Bam*HI-R; *PylRS-L3* gene: fragments 1 and 3 were from the *PylRS-L2* gene; fragment 2, Linker-3XG4S-F2 and Linker-3XG4S-R2.

The pCDF-*N-PylRS* plasmids with the inserted linker, pCDF-*N-PylRS-D1*, pCDF-*N-PylRS-L1*, pCDF-*N-PylRS-L2*, and pCDF-*N-PylRS-L3* (Figure 1B), were then constructed using the procedures mentioned above. pCDF-*ZRS* was derived from pEVOL-*mKRS1-pylT* (Wang Z.U. et al., 2012). The *N-PylRS* mutations were transplanted to *ZRS* by the methods mentioned earlier to construct pCDF-*N-ZRS*. The linkers were inserted into *ZRS* to generate pCDF-*N-ZRS-D1*, pCDF-*ZRS-L1*, pCDF-*ZRS-L2*, and pCDF-*ZRS-L3* plasmids. All the ligated products were transformed to *E. coli* DH5α, and the colonies were selected for DNA sequencing, respectively.

### Expression and Purification of ncAA-Encoded sfGFP

To produce ncAA-encoded sfGFP proteins, the pET-*pylT-sfGFP-TAG2* or pET-*pylT-sfGFP-TAG27* plasmid was co-transformed with different pCDF-*PylRS* variants into *E. coli* BL21 (DE3) individually. After an hour of recovery, the bacteria were spread on a plate containing ampicillin (Amp) (100 μg/ml) and streptomycin (Sm) (100 μg/ml). A single colony was chosen from the plate and cultured in 1 ml LB medium overnight. The cultured bacteria were then transferred to 50 ml fresh LB medium and incubated at 37°C until the OD_595_ reached 0.6–0.8. Protein expression was induced with the supplement of 1 mM IPTG and ncAA (except for sfGFP-**3** and sfGFP-**4** protein production, where the medium was changed to GMML medium supplemented with 2 mM ncAA) and incubated at 37°C for 12 h. The bacteria were then harvested and resuspended in lysis buffer [1X phosphate-buffered saline (PBS), pH 7.4] and sonicated. After centrifugation (60 min, 20,000 × *g*, 4°C), the supernatant was collected and incubated with 0.5 ml Ni^2+^-NTA resin (Roche) for protein purification. A total of 5 ml lysis buffer and 2.5 ml washing buffer (1X PBS, 5 mM imidazole, pH 7.4) were used to remove proteins bound non-specifically to the resin. The target protein was eluted from the resin with 2.5 ml elution buffer (1X PBS, 200 mM imidazole, pH 7.4). The buffer of the eluted fractions was changed to 1X PBS with Amicon Ultra-15 Centrifugal Filter Units (MWCO 10 kDa). Purified sfGFP was analyzed by 12% sodium dodecyl sulfate–polyacrylamide gel electrophoresis (SDS-PAGE) with instant blue staining.

### Western Blot Analysis

Whole cells were collected and lysed at 100°C with SDS loading dye for 15 min and then subjected to 12% SDS-PAGE analysis. The gels were stained with InstantBlue^TM^ Stain to visualize the target proteins with the expected molecular weight of around 28 kDa. The suppression efficiency of the amber codon in the sfGFP proteins with a C-terminal His tag was observed by western blot with an anti-6X His tag antibody. Western blots were performed using a Trans-Blot Turbo System (Bio-Rad) and an RTA transfer kit. Anti-His (SignalChem, H99-61M-100) and horseradish peroxidase (HRP)-conjugated secondary antibody (Cell Signaling Technology, 7076P2) were used for immunoblotting. After SDS-PAGE analysis, the gel was immersed in the transfer buffer and then blotted with a polyvinylidene fluoride (PVDF) membrane (25 V/1.3 A, 10 min). After the transfer process was finished, the PVDF membrane was washed thrice with phosphate-buffered saline with Tween^®^ (PBST) buffer for 5 min. Next, the membrane was blocked with 5% skimmed milk for 1 h at room temperature. Then, the membrane was washed thrice with PBST buffer for 5 min (washing step). The primary antibody (1:1,000 dilution) was added and incubated with the membrane for 1 h at room temperature following the washing step. Subsequently, the HRP-conjugated secondary antibody (1:5,000 dilution) was added and incubated with the membrane for 1 h at room temperature. The membrane was then treated by a washing step. Finally, the WesternBright ECL HRP substrate (Advansta, K-12045-D50) was mixed and spread onto the membrane to visualize the band signals using the ChemiDoc Imaging Systems (Bio-Rad) in bioluminescence detection mode.

### Electrospray Ionization-Mass Spectrometry Characterization of sfGFP

The pure protein was diluted with 50% acetonitrile and 1% formic acid. An aliquot corresponding to 1 pmol of the pure protein was injected into an ESI source (Waters LockSpray Exact Mass Ionization Source) with a syringe pump (Harvard Apparatus, MA, United States) and a flow rate of 5 μl/min was held throughout the analysis. The mass of the intact proteins was determined using Waters Synapt G2 HDMS mass spectrometer (Waters, Milford, MA, United States). The acquired spectra were deconvoluted to single-charge state using the MaxEnt1 algorithm of the MassLynx 4.1 software (Waters).

### Matrix-Assisted Laser Desorption/Ionization-Time of Flight-Tandem Mass Spectrometry Characterization of sfGFP

Details of in-gel digestion are as follows: after the staining procedure, the gel bands were excised and cut into small pieces. The gel pieces were sequentially washed with 25 mM NH_4_HCO_3_, 40% methanol solution, and 100% acetonitrile before being treated with DTT and then iodoacetamide. Washing of the gel pieces with 25 mM NH_4_HCO_3_ and 50% acetonitrile and then drying in a vacuum centrifuge provided the materials for trypsin digestion. A solution of 65 to 100 ng of sequencing-grade modified trypsin (Promega) in 25 mM NH_4_HCO_3_ and 10% acetonitrile (25–30 μl) was added and incubated with the gel pieces for 12–16 hrs at 37°C. The reaction was stopped by adding 1–2 μl of 5% formic acid. Details of the MS and data analysis are as follows: the digested samples (0.5 μl) were carefully mixed with the matrix solution (0.5 μl of 5 mg/ml DHB in 0.1% TFA/30% acetonitrile) and 0.5 μl of the mixture was deposited onto a MTP 600/384 AnchorChip (Bruker Daltonics). All mass spectrometry experiments were done using a Bruker Autoflex III MALDI TOF/TOF mass spectrometer (Bremen, Germany) equipped with a 200-Hz SmartBean Laser in positive ion mode with delayed extraction in the reflectron mode. Data acquisition was done manually with FlexControl 3.4, and data processing was performed with Flex-Analysis 3.4 (both Bruker Daltonik). Protein database searches through Mascot, using combined PMF and tandem mass spectrometry MS/MS datasets, were performed *via* Biotools 3.2 (Bruker).

### Determination of the Suppression Efficiencies of PylRS

To understand the substrate range of PylRS variants, screening of a 359 ncAAs library was performed. The plasmid pET-*pylT-sfGFP-TAG2* or pET-*pylT-sfGFP-TAG27* and pCDF-*PylRS* variants were co-transformed into *E. coli* BL21 (DE3) individually. The bacteria were spread on a plate supplemented with Amp (100 μg/ml) and Sm (100 μg/ml). The plate was incubated at 37°C overnight. Ten colonies were then inoculated and cultured in 3 ml LB medium at 37°C overnight before 500 μl of each cultured bacteria was transferred to 25 ml of fresh LB medium and incubated at 37°C until the OD_595_ reached 0.6–0.8. The cells were harvested and washed twice with M9 salts and suspended in M9 medium (M9 salts, 1% glycerol, 2 mM MgSO_4_, and 0.1 mM CaCl_2_) containing 1 mM IPTG. Aliquots (50 μl) of the suspended cells were loaded into a 384-well plate containing a different ncAAs (1 mM) in 359 wells (Supplementary Table S2). Cells were incubated in a plate reader (BioTek) at 37°C for 12 h, with continuous monitoring of the fluorescence intensity (excitation 535 nm and emission 595 nm) as well as OD_595_. Twelve wells were used as controls to measure the background signals (six wells without ncAAs and IPTG; six wells without ncAAs but containing IPTG). The fluorescence intensity of sfGFP was divided by the OD_595_ following the subtraction of the control signals (containing IPTG but no ncAAs) to generate the relative fluorescence intensity.

## Results

### Designing Active PylRS Variants With Mutations at or Near the N-Terminal Domain and Linker Engineering

**FIGURE 2:**
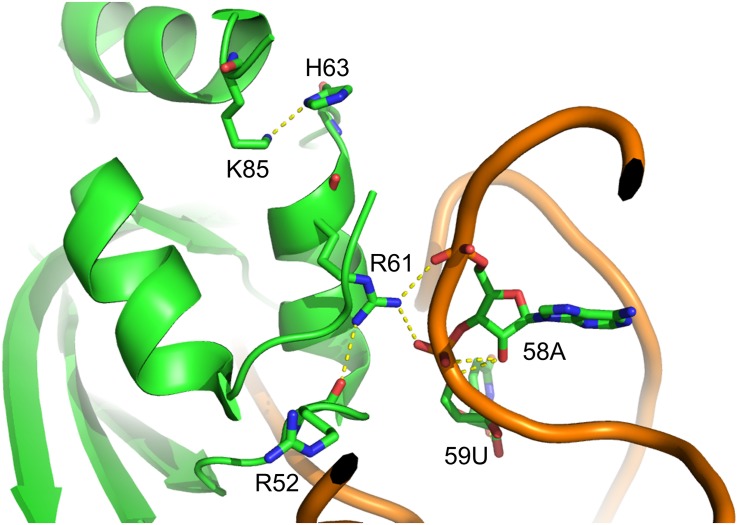
Structure of *Mm*PylRS NTD complex with tRNA^Pyl^. R61 and H63 located in the PylRS NTD; the yellow dashed lines represent potential hydrogen-bonding interactions. Three such interactions are illustrated between the side chain of R61 and R52 within PylRS NTD and the phosphodiester backbone of 58A and 59U in tRNA^Pyl^. One hydrogen-bonding interaction was found between the side chain of H63 and K85 within the PylRS NTD. The structure is based on the PDB entry 5UD5.

**FIGURE 3:**
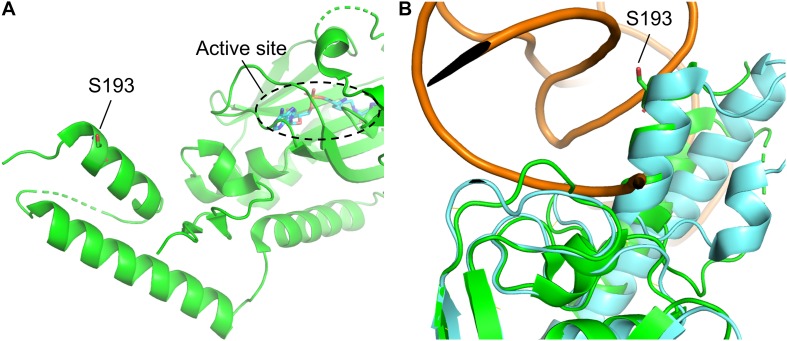
Structure of *Mm*PylRS CTD and its superimposition with the *Dh*PylRS CTD/tRNA^Pyl^ complex. **(A)** S193 is located in the tRNA binding domain of *Mm*PylRS CTD (green). **(B)** The superimposed structure of *Mm*PylRS CTD (green) and *Dh*PylRS CTD⋅*Dh*tRNA^Pyl^ complex (cyan). The structures are based on the PDB entry 2Q7H and 2ZNI.

To explore novel substrate ranges, this work aimed to generate novel PylRS variants without active site mutations for studying remote effects in altering the interaction between the tRNA and the PylRS NTD or CTD. In a previous study, the evolved PylRS, HarRS, had R61K, H63Y, S193R, N203T, L305H, L309W, N346D, C348S, L367M, Y384F, K429M, K431M, D433G, and G444E mutations. These mutations of HarRS have been found to enhance the activity and the selectivity in charging homoarginine (Mukai et al., 2015). To understand how the mutations on NTD affect the suppression efficiency of PylRS, the first three mutation sites in HarRS are transplanted to PylRS to generate N-PylRS (R61K/H63Y/S193R) (Figure 1). The R62K and H63Y mutations are in the NTD and at the interface between the PylRS-NTD and the tRNA T loop region (Figure 2). The S193R mutation is in the CTD and is located at the interface with the tRNA D-loop region (Figure 3) based on an overlapped model of the *Dh*PylRS CTD/tRNA^Pyl^ co-crystal and the *Mm*PylRS CTD crystal structures. In phage-assisted non-continuous evolution (PANCE) approach for evolving chPylRS, some mutants were found to be active in charging BocK with two separated genes by inserted TGA stop codon and following ATG start codon between NTD and CTD (Suzuki et al., 2017). The chPylRS was generated by fusing *Mb*PylRS NTD (1–149 residues) with the *Mm*PylRS C-terminus (185–454 residues). In this work, wild-type *Mm*PylRS (wt-PylRS) was used to translate the inserted mutation between P149 and A150 to form the PylRS-D1 construct (Figure 1B) in generating *Mm*PylRS NTD (1–149 residues) and *Mm*PylRS CTD (150–454 residues) proteins. The truncated *Mm*PylRS NTD (1–149 residues), namely, PylRS-ND, was also generated for comparison.

**FIGURE 4:**
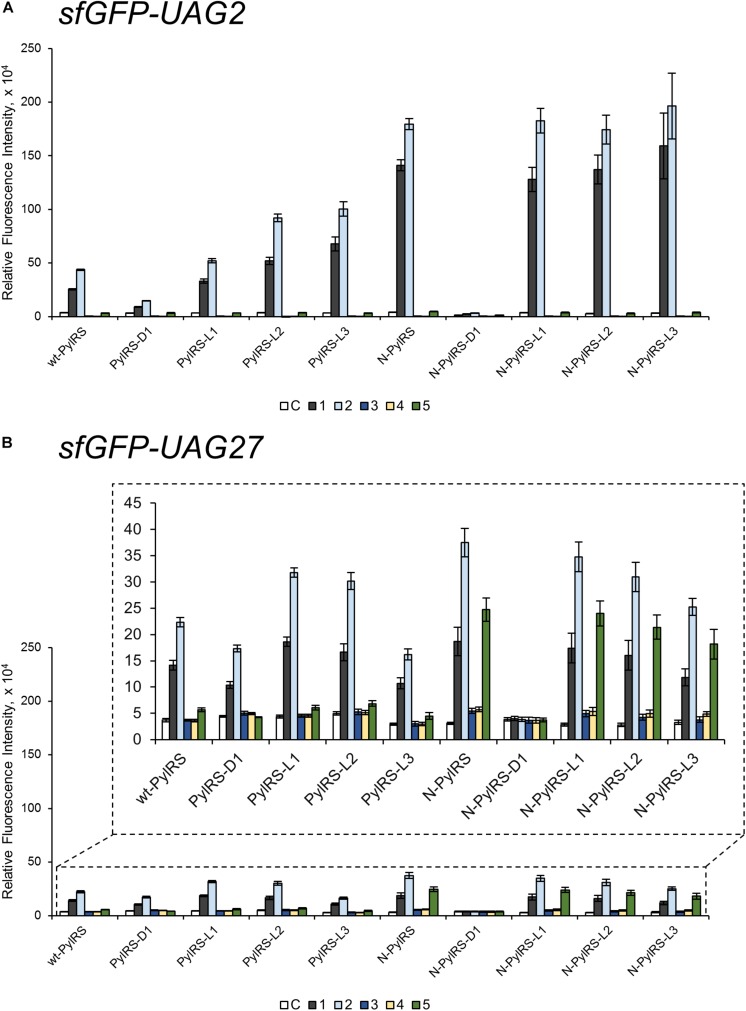
The *sfGFP-UAG2* and *sfGFP-UAG27* gene suppression efficiencies of PylRS enzyme variants. Incorporation efficiencies of PylRS variants (Figure 1B) as measured by fluorescence intensities of sfGFP with an amber mutation at position 2 **(A)** and 27 **(B)**. The proteins were expressed in 1 mM ncAA and IPTG in GMML medium at 37°C for 12 h. The cells were excited at 485 nm and the fluorescence intensities were detected at 535 nm. The cell density was monitored by absorbance at 595 nm. “C” indicates the Control experiments of cells with the supplement of 1 mM IPTG; 1–5 denote the supplement of 1 mM IPTG and ncAA **1**–**5** (Scheme 1). The background signals from cells without adding IPTG were subtracted from each group. The error bars represent the standard deviation of sfGFP production from four repeated experiments.

To probe the crosstalk between the PylRS NTD and the CTD in charging amino acid substrates, three flexible loops of different lengths were inserted between these domains in PylRS-D1. The linkers were the hexapeptide SGGGGS (PylRS-L1), the tridecapeptide S(GGGGS)_2_ (PylRS-L2), and the non-adecapeptide S(GGGGS)_3_ (PylRS-L3) (Figure 1B). In addition to these three mutants, we also compared wt-PylRS and PylRS-D1; these five PylRS variants were subjected to test substrate range with the supplement of co-transformed *E. coli* carrying *MmPylRS/tRNA^Pyl^* gene cassettes and reporter gene *sfGFP-TAG2* or *sfGFP-TAG27*, respectively. The fluorescence intensities of the *sfGFP-UAG2* and *sfGFP-UAG27* gene products indicated the read-through of the amber codon in response to the ncAA. Generally, the *sfGFP-UAG2* suppression test has 4.7 to 1.6 times higher signal than *sfGFP-UAG27* suppression test in charging ncAA **1** and **2** (Scheme 1), but the reverse results are seen with ncAA **3**–**5**. After producing the *sfGFP-UAG2* and *sfGFP-UAG27* gene products, 359 ncAAs (see Supplementary Table S1 for structures) were tested as substrates. The screening results (Supplementary Figures S15–S24) show various intensities of signals in charging BocK (**1**) and AlloK (**2**) (Scheme 1), which are considered as good substrates for wt-PylRS recognition (Yanagisawa et al., 2008). In *sfGFP-UAG2* gene production, PylRS-D1 preserved 34% of activity in charging AlloK (**2**) compared to wt-PylRS. The PylRS variants with linkers generate better activities (Figure 4A). All three PylRS variants (PylRS-L1, PylRS-L2, and PylRS-L3) were rescued by inserted linkers and showed enhanced activity at 120–230% compared to the activity of wt-PylRS. In *sfGFP-UAG27* gene production (Figure 4B), however, the activity of all five PylRS variants maintained a similar pattern with less charging of ncAA **1** and **2**. Small signals in charging 3MeH (**5**) (Scheme 1) were found in wt-PylRS, PylRS-L1, PylRS-L2, and PylRS-3 in this amber suppression test.

The effects of PylRS-R61K, PylRS-H63Y, and PylRS-S193R variants were determined and found to improve the suppression efficiency against ncAA **1**–**2** in *sfGFP-UAG2* production compared to wt-PylRS, whereas no fluorescence signals were observed in PylRS-ND (Supplementary Figure S47). Combining the three beneficial mutations, N-PylRS harboring R61K/H63Y/S193R mutations was evaluated in its substrate range by *sfGFP-UAG2* and *sfGFP-UAG27* gene production (Supplementary Figures S25, S26). Thus, we decided to investigate N-PylRS and its four variants, N-PylRS-D1 and N-PylRS-L1–L3 (Supplementary Figures S27–S34). Noticeably, N-PylRS showed nearly 5.6 and 4.1 times higher fluorescent signals in charging BocK (**1**) and AlloK (**2**) than wt-PylRS in the *sfGFP-UAG2* gene suppression yield (Figure 4A). In the *sfGFP-UAG27* gene suppression study, N-PylRS was found to recognize ncAA **1**–**5**. While AlloK (**2**) is still the best substrate, 3MeH (**5**) has a higher signal than BocK (**1**) (Figure 4B). In addition, N-PylRS was capable of incorporating *S*-benzyl cysteine analogs MbzlC (**3**) and MeObzlC (**4**) in low suppression efficiencies in *sfGFP-UAG27* production. In contrast to PylRS-D1, the substrate specificity profiles of N-PylRS-D1 have revealed abolished fluorescence intensities in charging ncAA **1**–**5** in *sfGFP-UAG2* and *sfGFP-UAG27* suppression tests. The N-PylRS-L1–L3 variants showed reinstalled signals in *sfGFP-UAG2* suppression with similar activities in charging BocK (**1**) and AlloK (**2**). The *sfGFP-UAG27* gene suppression test in N-PylRS-L1–L3 had a gradual decrease in activity along with increasing linker length in charging ncAA **1**–**5**.

### Substrate Range Study of ZRS and Its Variants

**FIGURE 5:**
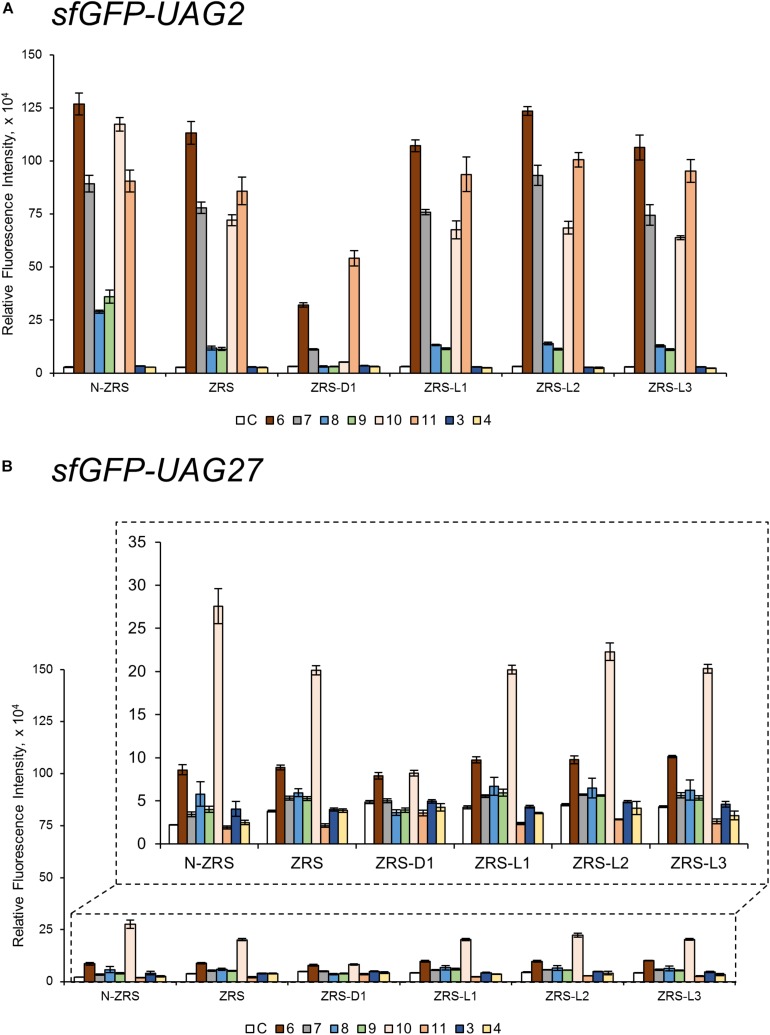
The *sfGFP-UAG2* and *sfGFP-UAG27* gene suppression efficiencies of ZRS enzyme variants. Incorporation efficiencies of ZRS variants (Figure 1B) as measured by the fluorescence intensities of sfGFP with amber mutation at position 2 **(A)** and 27 **(B)**. The proteins were expressed in 1 mM ncAA and IPTG in GMML medium at 37°C for 12 h. The cells were excited at 485 nm and the fluorescence intensities were detected at 535 nm. The cell density was monitored by absorbance at 595 nm. “C” indicate the Control experiments of cells with the supplement of 1 mM IPTG; **3**, **4**, **6**–**11** denote the supplement of 1 mM IPTG and ncAA **3**, **4**, **6**–**11** (Scheme 1). Error bars represent the standard deviation of sfGFP production from four repeated experiments.

ZRS was evolved from *Mm*PylRS with Y306M/L309A/C348T/T364K/Y384F mutations at the active site (Wang et al., 2010). To validate the influence on the activity of ZRS by the R61K/H63Y/S193R mutations, as well as the introduction of the linker, these variations in the wt-PylRS study were transplanted and tested on ZRS, which was initially found to incorporate CbzK (**6**) and CbzK analogs (**7**, **10**–**11**) (Scheme 1) (Wang Z.U. et al., 2012). Thus, ZRS variants, N-ZRS, ZRS-D1, ZRS-L1, ZRS-L2, and ZRS-L3, were constructed for evaluating their effects with active site mutation. The screening results of the substrate range in *sfGFP-UAG2* and *sfGFP-UAG27* suppression for the ZRS and the five variants are shown in Supplementary Figures S35–S46. NcAA **3**–**11** (Scheme 1) had a positive response and are illustrated in Figure 5.

CbzK (**6**) was efficiently incorporated into sfGFP in response to the amber codon as reported for ZRS. The CbzK analogs, ClCbzK (**7**), SeCbzK (**10**), and CbzKOH (**11**) (Scheme 1), also showed similar intensity in the *sfGFP-UAG2* suppression study. Two D-form CbzK analogs, DCbzK (**8**) and DClCbzK (**9**), were positive but had lower signals (Figure 5A). *sfGFP-UAG27* suppression in ncAAs library screening of ZRS showed 12.8 times lower signal in charging CbzK compared to the *sfGFP-UAG2* suppression study but was only 1.6 times lower in charging SeCbzK. The substrate range of N-ZRS remained the same as that of ZRS; nevertheless, the substrate specificity profiles of N-ZRS in the *sfGFP-UAG2* suppression study showed a higher fluorescence intensity as compared to ZRS, especially in charging D-ncAA **8** and **9**. Unexpectedly, *S*-benzyl cysteine analogs ncAA **3** and **4** were also incorporated into sfGFP with low efficiencies by N-ZRS in the *sfGFP-UAG27* suppression study. The ZRS-D1 screening results showed a significant decrease in activity in the *sfGFP-UAG2* and *sfGFP-UAG27* suppression studies compared to ZRS and other ZRS variants. The ZRS-D1 activity with CbzKOH (**11**), however, was the best in the *sfGFP-UAG2* screening and had diminished activity in the *sfGFP-UAG27* screening (Figure 5). The addition of a linker did not raise the suppression efficiencies of ZRS albeit a slight increase in the fluorescence intensities of ZRS-L2 can be observed with ncAA **6** and **10** in response to *sfGFP-UAG2* and *sfGFP-UAG27* (Figure 5).

### Western Blotting, Electrospray Ionization-Mass Spectrometry, and Tandem Mass Spectrometry Characterization of ncAA-Encoded sfGFP

**FIGURE 6:**
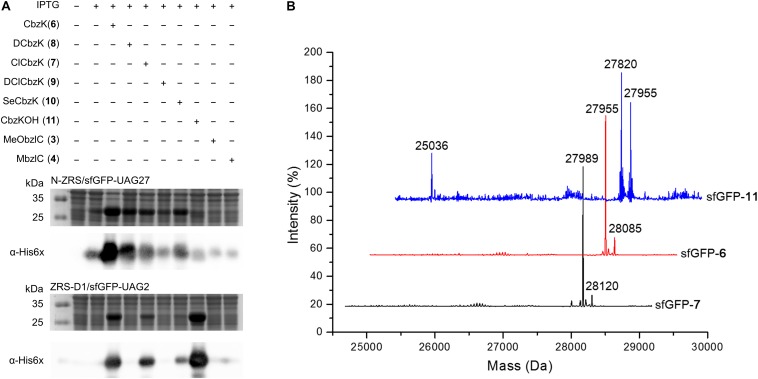
sfGFP production by ZRS variants and mass characterization. **(A)** Amber suppression of the *sfGFP-UAG27* gene (N-ZRS⋅tRNA^Pyl^ pair) and the *sfGFP-UAG2* gene (ZRS-D1⋅tRNA^Pyl^ pair) product with ncAA **3**–**4** and **6**–**11**. The sfGFP proteins were produced in *E. coli* BL21 (DE3) coding N-ZRS⋅tRNA^Pyl^ or ZRS-D1⋅tRNA^Pyl^ pair with the supplement of 1 mM IPTG and ncAAs in GMML medium at 37°C for 12 h. The whole-cell lysate was analyzed by SDS-PAGE and western blotting by anti-His tag antibody indicated as α-His6X. **(B)** ESI-MS determination of sfGFP-UAG27 proteins with ncAA **6**, **7**, and **11**. Full-length sfGFP-**6** and sfGFP-**7** proteins were produced by N-ZRS⋅tRNA^Pyl^ pair in *E. coli* BL21 (DE3) with the supplement of 1 mM IPTG and ncAA **6** or **7** in LB medium at 37°C for 12 h. Full-length sfGFP-**11** proteins were produced with the same condition but with 1 mM ncAA **11** and in GMML minimal medium. The calculated molecular masses of sfGFP-**7** are 28,121 and 27,989 Da (–Met); the observed molecular masses are 28,120 and 27,989 Da (–Met). The calculated molecular masses of sfGFP-**6** are 28,085 and 27,954 Da (–Met); the observed molecular masses are 28,085 and 27,955 Da (–Met). The calculated molecular masses of sfGFP-**11** are 28,086, 27,955 (–Met), and 25,320 Da (truncated sfGFP at 27 position); the observed molecular masses are 27,955 (–Met) and 27,820 Da (without Cbz group at 27 position and N-terminal Met residues) and 25,036 Da. The detailed electrospray and deconvoluted mass spectra are shown in Supplementary Figures S4–S6. ESI-MS determination of sfGFP-UAG2 protein. sfGFP-11* with ncAA **11** (ZRS-D1⋅tRNA^Pyl^ pair) is shown in Supplementary Figure S7.

Some amber screening results show reverse or different intensity patterns, and western blotting analysis was used to confirm sfGFP protein yield to support the sfGFP fluorescence intensity results. N-ZRS and ZRS-D1 were chosen for analyzing sfGFP-UAG27 and sfGFP-UAG2 protein productions (Figure 6A). An anti-His tag antibody was used to detect the C-terminal-His tag present in full-length sfGFP, which indicates the amount of read-through at the amber stop codon. Western blotting analysis of N-ZRS/sfGFP-UAG27 indicates strong amber read-through for ncAA **6**–**8** and **10** and weaker response for ncAA **9** and **11** in SDS-PAGE and anti-His channel. Clearly, sfGFP-**11** showed an additional band at lower molecular weight, near the 25-kDa protein marker in SDS-PAGE, which was also detected by western blotting (Figure 6A). These results are in partial agreement with the sfGFP fluorescent screening. They match for ncAA **3**, **4**, **9**, and **10**, not for ncAA **6**–**8** and **11** (Figure 5B). A ZRS-D1/sfGFP-UAG2 analysis (Figure 6A), however, matches well with sfGFP fluorescent screening results (Figure 5A), confirming that ZRS-D1 charges CbzKOH to generate acylated tRNA with high activity. The purified sfGFP-**11**^∗^ protein produced by the ZRS-D1⋅tRNA^Pyl^ pair generated two additional major mass peaks because of ester bond cleavage and Cbz group deprotection: 27,773 Da (without the N-terminal Met) and 27,639 Da (without the N-terminal Met and Cbz group) (Table 1 and Supplementary Figure S7). Purified sfGFP-**6**, sfGFP-**7**, and sfGFP-**11**, which were produced by N-ZRS⋅tRNA^Pyl^ pair, were analyzed by electrospray ionization-mass spectrometry (ESI-MS) (Table 1 and Figure 6B, Supplementary Figures S4–S6). The experimental mass of sfGFP-**6** (28,086 and 27,955 Da) and sfGFP-**7** (28,120, and 27,988 Da) matched well to the calculated molecular weight of sfGFP-**6** (28,085 and 27,955 Da) and sfGFP-**7** (28,120 and 27,989 Da). A matrix-assisted laser desorption/ionization-time of flight-tandem mass spectrometry (MALDI-TOF-MS/MS) analysis of peptide ^27^XSV^30^R fragments which encoded ncAA **6** and **7** further provides the correct molecular mass and sufficient b and y fragments to indicate ncAA purity at position 27 of the sfGFP (Table 1 and Supplementary Figures S10, S11). In characterizing the mass of sfGFP-**11**, the replacement of the amino group with a hydroxyl group in the main chain results in additional mass peaks. Three mass peaks, at 27,955, 27,820, and 25,036 Da, were observed in the sfGFP-**11** mass spectrum. The calculated mass of full-length and truncated sfGFP-**11** is 27,955 (-Met) and 25,320 Da, respectively. The full-length sfGFP-**11** mass agrees with the calculated mass, and the peak at 27,820 Da matches the expected mass of sfGFP-**11** with Cbz group deprotection. The mass of sfGFP truncated at 28 position, 25,036 Da, does not fully match the calculated mass 25,057 Da and instead shows a loss of 21 Da, which indicates CbzKOH deletion at 27 position of the truncated sfGFP-**11** (Table 1 and Figure 6B, Supplementary Figure S6).

**TABLE 1:** Electrospray ionization-mass spectrometry (ESI-MS) analysis of sfGFP proteins.

Protein^1^	PylRS mutants	Calculated mass (Da)	Actual mass (Da)	ESI-MS^4^	MALDI-TOF-MS/MS^5^
sfGFP-**3**	N-PylRS	28,030, 27,899 (-Met)^2^	28,030, 27,899	S1	8A
sfGFP-**4**		28,046, 27,915 (-Met)	28,046, 27,915	S2	S14
sfGFP-**5**		27,974, 27,843 (-Met)	27,974, 27,843	S3	8B
sfGFP-**6**	N-ZRS	28,086, 27,955 (-Met)	28,085, 27,955	S4	S10
sfGFP-**7**		28,120, 27,988 (-Met)	28,120, 27,989	S5	S11
sfGFP-**11**		27,955 (-Met), 27,820^6^, 25,057^7^	27,955, 27,820, 25,036	S6	–
sfGFP-**3**		27,899 (-Met)	27,899, 27,878^3^	S8	S12
sfGFP-**4**		27,915 (-Met)	27,916, 27,878^3^	S9	S13
sfGFP-**11**^∗^	ZRS-D1	27,904, 27,773 (-Met), 27,639^8^	27904, 27,773, 27,639	S7	–

*The sfGFP protein was produced from *sfGFP-UAG2* gene. sfGFP-**3**, sfGFP-**4**, sfGFP-**11**, and sfGFP-11* were produced in GMML medium; sfGFP-**5**, sfGFP-**6**, and sfGFP-**7** were prepared in LB medium. ^*1*^The sfGFP proteins were produced from *sfGFP-UAG27* gene. ^*2*^(–Met) indicates full-length sfGFP protein without N-terminus methionine residues. ^*3*^Actual mass of 27,878 Da is full-length sfGFP with tryptophan incorporation at F27 position without N-terminus methionine (calculated mass: 27,878 Da). ^*4*^Full-length sfGFP electrospray and deconvoluted mass spectrometries. ^*5 27*^XSV^*30*^R peptide fragment was selected for MALDI-TOF-MS/MS analysis. ^*6*^27,820 Da is the calculated molecular weight of sfGFP without Cbz group at 27 position and N-terminal Met residues. ^*7*^25,057 Da is the calculated mass of truncated sfGFP at 28 position. ^*8*^27,639 Da is the calculated mass of sfGFP without N-terminal Met and Cbz group on the CbzKOH side chain.

**FIGURE 7:**
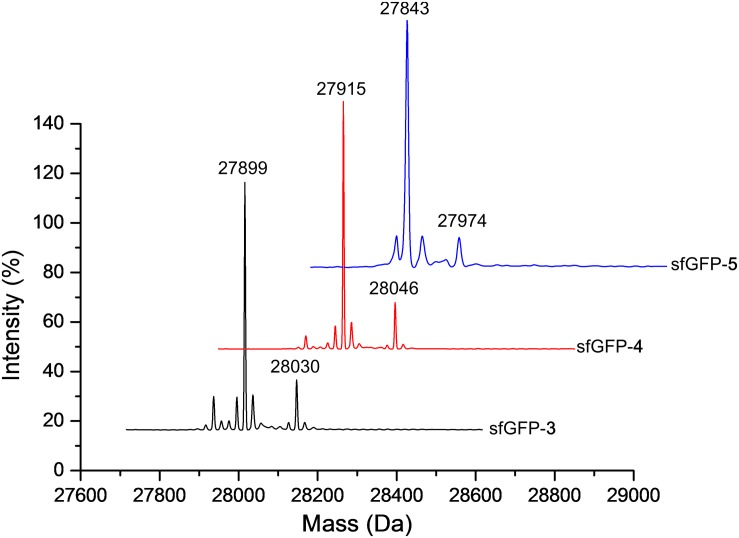
Deconvoluted ESI-MS spectra of sfGFP containing ncAAs at position 27. Full-length sfGFP-**3**, sfGFP-**4**, and sfGFP-**5** were expressed using the N-PylRS⋅tRNA^Pyl^ pair in the presence of ncAA **3**, **4** (2 mM) or **5** (1 mM), respectively, in GMML medium (sfGFP-**3** and sfGFP-**4)** or LB medium (sfGFP-**5**). The purified protein samples were subjected to ESI-MS for identification of the incorporation of defined ncAA. Deconvoluted ESI-MS of ncAA **3**–**5** incorporated sfGFP at position 27 are shown above. sfGFP-X in which X represents one of the ncAA **3**–**5** was incorporated into sfGFP at position 27. The calculated molecular masses of sfGFP-**3** are 28,030 and 27,899 Da (-Met); the observed molecular masses are 28,030 and 27,899 Da (-Met). The calculated molecular masses of sfGFP-**4** are 28,046 and 27,915 Da (-Met); the observed molecular masses are 28,046 and 27,915 Da (-Met). The calculated molecular masses of sfGFP-**5** are 27,974 and 27,843 Da (-Met); the observed molecular masses are 27,974 and 27,843 Da (-Met). The detailed electrospray and deconvoluted mass spectra are shown in Supplementary Figures S1–S3.

**FIGURE 8:**
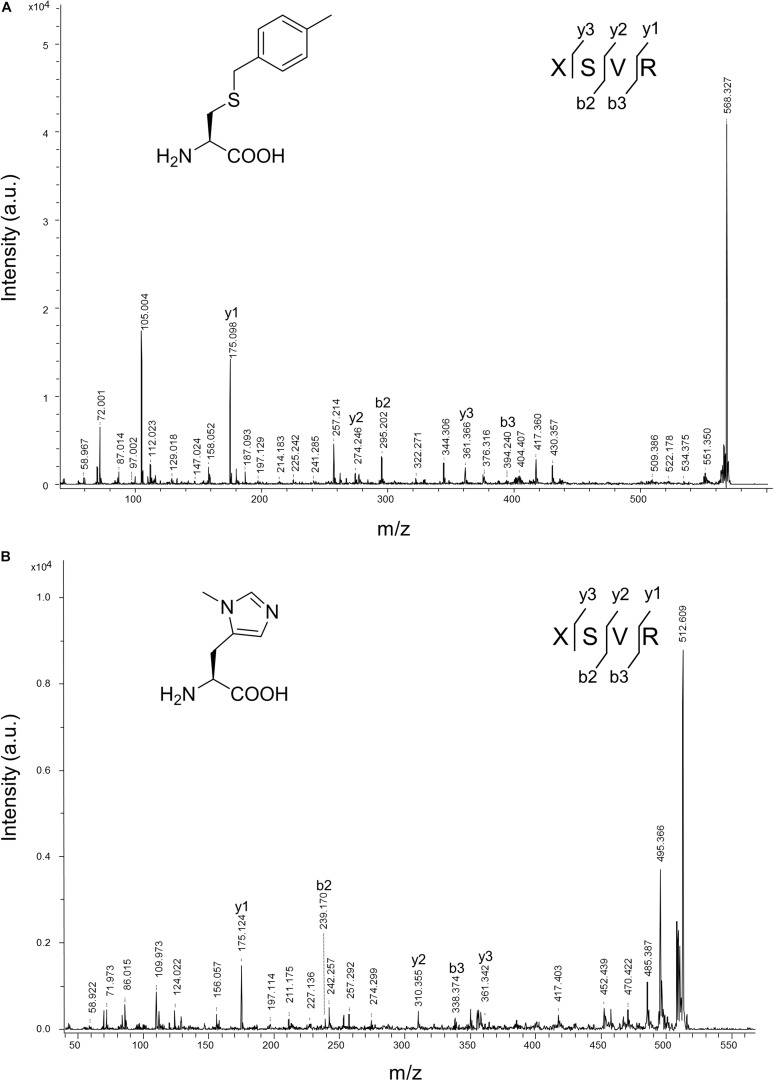
MALDI-TOF-MS/MS analysis of sfGFP-3 and sfGFP-5. The sfGFP-**3** and sfGFP-**5** proteins were in-gel digested with trypsin and subjected to MALDI-TOF-MS/MS analysis. **(A)** The tandem mass spectrum of the X(**3**)SVR; X denotes a fragment from sfGFP-**3**, with ncAA **3** incorporated at position 27. The calculated molecular mass of the X(**3**)SVR peptide fragment is 567.284 Da and the actual molecular mass is 568.327 Da. Full-length sfGFP-**3** was expressed using N-PylRS in the presence of 2 mM ncAA **3** in GMML medium. **(B)** The tandem mass spectrum of the X(**5**)SVR; X denotes a fragment from sfGFP-**5** with ncAA **5** incorporated at position 27. The calculated molecular mass of the X(**5**)SVR peptide fragment is 511.287 Da and the actual molecular mass is 512.609 Da. Full-length sfGFP-**5** was produced using N-PylRS⋅tRNA^Pyl^ pair in the presence of 1 mM ncAA **5** in LB medium.

The purified sfGFP-**3** and sfGFP-**4** proteins with ncAA **3** and **4** encoded at 27 position that were produced by the N-ZRS⋅tRNA^Pyl^ pair in *E. coli* were characterized, and they matched the calculated mass (Table 1 and Supplementary Figures S8, S9). The mass spectra of sfGFP-**3** and sfGFP-**4** show a molecular peak indicating tryptophan incorporation, 27,878 Da. A MALDI-TOF-MS/MS analysis of sfGFP-**3** and sfGFP-**4** also confirmed the presence of ncAA **3** and **4** at position 27 of sfGFP. To confirm the ncAAs screening results mentioned in the N-PylRS⋅tRNA^Pyl^ pair study, sfGFP-**3**, sfGFP-**4**, and sfGFP-**5** were expressed and purified, respectively, and then subjected to ESI-MS analysis (Table 1 and Supplementary Figures S1–S3). The calculated molecular weight of sfGFP-**3**, sfGFP-**4**, and sfGFP-**5**, generated by N-PylRS, matched the observed mass in the full-length sfGFP (Figure 7). The incorporation of ncAA **3**, **4**, and **5** into sfGFP at position 27 was shown by MALDI-TOF-MS/MS (Figure 8 and Supplementary Figures S12–S14). This multiple evidence indicates that MbzlC (**3**), MeObzlC (**4**), and 3MeH (**5**) are incorporated into proteins by non-active, mutated N-PylRS⋅tRNA^Pyl^ pair site-specifically.

## Discussion

In this study, we showed that the R61K/H63Y/S193R mutations, which are located beyond the catalytic site, increase the suppression efficiencies of PylRS and ZRS toward their preferred substrates. The substrate range of N-PylRS allowed the incorporation of ncAA **3**–**5**, two S-benzyl cysteine analogs and a histidine analog, with high efficiency as compared to wt-PylRS. This suggests that the remote mutations tune the interaction between PylRS and tRNA^Pyl^, which leads to the successful incorporation of ncAA **3**–**5** despite the active site engineering of PylRS (Xiao et al., 2014; Sharma et al., 2016). A similar tuning effect has been observed in PylRS N-terminal mutations by global-directed evolution and PANCE, which results in enhancement of enzyme efficiencies (Sharma et al., 2018). We chose R61 and H63 of PylRS as they are clustered on the loop adjacent to the extremity of the α-helix. Specifically, the guanidinium side-chain of R61 has a polar interaction with the phosphodiester backbone near 58A of tRNA^Pyl^ as well as the R52 residue within the NTD. In addition, the imidazole side-chain of H63 hydrogen bonds with the amino group on the side chain of K85 (Figure 2). The R61K and H63Y mutations may disrupt these interactions. The S193 is located on the tRNA binding domain of the PylRS CTD. Although S193 is relatively far from the NTD, it does not interact with residues alongside the catalytic site. We envision that the S193R mutation moderately tunes the binding between PylRS and tRNA^Pyl^ (Figure 3). By placing the R61K/H63Y/S193R mutations on wt-PylRS, the substrate range was shifted to introduce ncAA **3**–**5**. We postulate that the catalytic core of wt-PylRS expands locally to harbor ncAA with shorter bulky side-chains owing to the sophisticated regulation with the binding of tRNA^Pyl^. The ZRS shows its best incorporation efficiencies toward ncAA **6**, and it was also found to incorporate ncAA **7**, **3**, and **4** into sfGFP based on the screening results. Unanticipatedly, the substrate range of N-ZRS remained the same as that of ZRS. The fine-tuning effect of the mutations seen with wt-PylRS is not seen with ZRS, which might result from its tightly regulated substrate range of the evolved catalytic pocket. This result also implies the current directed-evolution approach at the PylRS active site which leads to a stabilized local arrangement that prevents it from the tuning effects of N-terminal mutations. This also explains no significant activity enhancement in introducing a linker to N-PylRS and ZRS. Although no significant improvement of an introduced linker was found, the repeated peptide sequence, (GGGGS)_1__–__3_, could be evolved to explore additional interaction with tRNA^Pyl^ for altering substrate range and activity enhancement. Importantly, the D-form ncAA **8** and **9** gave small but positive signals in ZRS and a yield enhancement in N-ZRS based on fluorescence screening and western blotting analysis. ZRS-D1 with separated NTD and CTD seems tightening up the chiral specificity by releasing the structural remote effects, which results in a losing activity in charging the D-form ncAAs **8** and **9** (Figures 5, 6A).

Two different sfGFP proteins, namely, sfGFP-UAG2 and sfGFP-UAG27, were used in this study to compare the incorporation efficiencies of the PylRS variants. Our studies have proven that the former fluorescence intensity of lysine derivatives was considerably stronger than the latter one. This indicates that the serine 2 of sfGFP located on the flexible loop might be suitable for the long and polar side-chains of ncAAs. On the contrary, the latter substrate range of amino acids with bulky and aromatic side-chain derivatives were easier to be observed than the former one. However, western blotting analysis of N-ZRS in producing the sfGFP-UAG27 proteins by installing ncAA **6**–**9** (Figure 6A) shows reverse intensity in fluorescent screening. This suggests that the sfGFP variants containing these ncAAs have lost their folded structure, resulting in fluorescent quenching by penetrating water molecules or protein precipitation. Surprisingly, sfGFP-**10** has a higher fluorescent signal than would be expected due to the western-blotting results. This may be due to a smaller bond angle at the selenium atom that causes a bended side-chain and stabilizes the sfGFP β-barrel structure. The characterization of sfGFP-**11** and sfGFP-**11**^∗^, which are produced by N-ZRS and ZRS-D1, also indicate ester bond hydrolysis and partial Cbz group deprotection.

Various PylRS have been discovered in nature to catalyze the acylation reactions between pyrrolysine and tRNA^Pyl^. Previous studies have shown that PylRS can be expressed in the form of either a single polypeptide (*Mm*PylRS or *Mb*PylRS) or separated polypeptides (*Dh*PylRS) (Jiang and Krzycki, 2012). ΔNPylRS is a group of PylRS which lacks the NTD but which shows decent suppression efficiencies *in vivo* (Willis and Chin, 2018). Our findings demonstrate that the separated polypeptide of *Mm*PylRS, PylRS-D1, still retains the lower catalytic activity, but this was not the case for N-PylRS-D1. Noticeably, the substrate specificity profiles of N-PylRS-D1 showed a decrease in activity as compared to PylRS-D1. The abolished activity of N-PylRS-D1 implies that the R61K/H63Y/S193R mutations need a linkage between the NTD and the CTD to transfer the remote effect in improving enzyme activity. In addition, our results point out the indispensability of the unstructured flexible loop connecting the NTD and the CTD of PylRS. The separately expressed NTD and CTD of *Mm*PylRS are believed to be functionally reassembled in cells according to the substrate specificity profiles of PylRS-D1, showing the reasonable decrease in activity toward the same substrates in contrast to wt-PylRS. Recombining the linker between the junctions has conspicuously restored the activity of N-PylRS-D1. This suggests that the tuning effect of R61K/H63Y/S193R between PylRS and tRNA^Pyl^ could also mutually regulate the interplay between the NTD and the CTD and that the truncation of the flexible loop might perturb their binding mode, leading to a reduction in activity. Increasing the length of a linker leads to an upward trend in the suppression efficiencies of wt-PylRS, whereas this effect is not observed with ZRS. These findings lead us to believe that the identity of the residues in the linker affects PylRS activity *via* tuning flexibility interactions involving the NTD and the CTD or providing beneficial interactions between linker residues and tRNA.

## Conclusion

Introduction of R61K/H63Y/S193R mutations to wt-PylRS has altered its substrate range for the incorporation of ncAA **3**–**5**, which, we propose, arises from the refinement of the dynamics between PylRS and tRNA^Pyl^. The addition of a linker provides extra flexibility to potentially regulate the interaction between NTD and CTD of PylRS and changes the interface between PylRS and tRNA^Pyl^, in turn enhancing the suppression efficiencies. Our findings not only indicate the sophisticated interaction between NTD and CTD of PylRS and tRNA^Pyl^ but also reveal the importance of N-terminus engineering in exploring the novel substrate range and improving the suppression efficiency.

## Data Availability Statement

All datasets generated for this study are included in the article/Supplementary Material.

## Author Contributions

Y-SW: conceptualization, resources, supervision, project administration, and funding acquisition. H-KJ, J-CT, K-WC, H-WT, K-PC, Y-KL, and Y-SW: methodology. Y-SW and H-KJ: validation. H-KJ and M-NL: formal analysis. H-KJ, M-NL, and Y-SW: data curation. H-KJ: writing – original draft preparation. Y-SW, Y-KL, and H-KJ: writing – review and editing.

## Conflict of Interest

The authors declare that the research was conducted in the absence of any commercial or financial relationships that could be construed as a potential conflict of interest.
