# Supplementary material for: Linker and N-Terminal Domain Engineering of Pyrrolysyl-tRNA Synthetase for Substrate Range Shifting and Activity Enhancement
Source: Front Bioeng Biotechnol. 2020 Apr 7;8:235. doi: 10.3389/fbioe.2020.00235 (PMC7156790; doi:10.3389/fbioe.2020.00235)
Supplement: Supplementary file 1 [file Data_Sheet_1.pdf]

Linker and N-terminal domain engineering of pyrrolysyl-tRNA synthetase for substrate range shifting and activity enhancement

Han-Kai Jiang[1,2,3], Man-Nee Lee[1], Jo-Chu Tsou[1], Kuan-Wen Chang[1], Hsueh-Wei Tseng[1], Kuang-Po Chen[4], Yaw-Kuen Li[4] and Yane-Shih Wang[1,2,5*]

[1] Institute of Biological Chemistry, Academia Sinica, Taipei, Taiwan, [2] Chemical Biology and Molecular Biophysics Program, Taiwan International Graduate Program, Academia Sinica, Taipei, Taiwan, [3] Department of Chemistry, National Tsing Hua University, Hsinchu, Taiwan, [4] Department of Applied Chemistry, National Chiao Tung University, Hsinchu, Taiwan, [5] Institute of Biochemical Sciences, National Taiwan University, Taipei, Taiwan

[*] **Correspondence:**
Yane-Shih Wang
yaneshihwang@gate.sinica.edu.tw

SUPPLEMENTARY MATERIALS

Table of contents

1. DNA and Protein Sequences 3
2. Primers list 4
3. Supplementary Figures 5
4. Table S1. Chemical Names and Structures in ncAAs Library 34
5. Table S2. Arrangement of ncAAs Library 64

1. DNA and Protein Sequences

MmPylRS:
atggataaaaaaccactaaacactctgatatctgcaaccgggctctggatgtccaggaccggaacaattcataaaataaaacaccacgaagt
ctctcgaagcaaaatctatattgaaatggcatgcggagaccaccttgttgtaaacaactccaggagcagcaggactgcaagagcgctcaggc
accacaaatacaggaagacctgcaaacgctgcagggtttcggatgaggatctcaataagttcctcacaaaggcaaacgaagaccagacaa
gcgtaaaagtcaaggtcgtttctgcccctaccagaacgaaaaaggcaatgccaaaatccgttgcgagagccccgaaacctcttgagaatac
agaagcggcacaggctcaaccttctggatctaaattttcacctgcgataccggtttccacccaagagtcagtttctgtcccggcatctgtttcaac
atcaatatcaagcatttctacaggagcaactgcatccgcactggtaaaagggaatacgaaccccattacatccatgtctgcccctgttcaggca
agtgcccccgcacttacgaagagccagactgacaggcttgaagtcctgttaaacccaaaagatgagatttccctgaattccggcaagcctttc
agggagcttgagtccgaattgctctctcgcagaaaaaaagacctgcagcagatctacgcggaagaaagggagaattatctggggaaactcg
agcgtgaaattaccaggttctttgtggacaggggttttctggaaataaaatccccgatcctgatccctcttgagtatatcgaaaggatgggcattg
ataatgataccgaactttcaaaacagatcttcagggttgacaagaacttctgcctgagacccatgcttgctccaaacctttacaactacctgcgc
aagcttgacagggccctgcctgatccaataaaaatttttgaaataggcccatgctacagaaaagagtccgacggcaaagaacacctcgaag
agtttaccatgctgaacttctgccagatgggatcgggatgcacacgggaaaatcttgaaagcataattacggacttcctgaaccacctgggaat
tgatttcaagatcgtaggcgattcctgcatggtctatggggataccettgatgtaatgcacggagacctggaactttcctctgcagtagtcggacc
cataccgcttgaccgggaatggggtattgataaaccctggataggggcaggtttcgggctcgaacgccttctaaaggttaaacacgactttaaa
aatatcaagagagctgcaaggtccgagtcttactataacgggatttctaccaacctgtaa

tRNA$_{CUA}^{Pyl}$:
*ggaaacctgatcatgtagatcgaatggact**cta**aatccgttcagccgggttagattcccggggtttccgcca*

The anticodon CTA is labeled in bold type.

sfGFP:
atgagcaagggcgaagaactgtttacgggcgtggtgccgattctggtggaactggatggtgatgtcaatggtcacaaattcagcgtgcgcggc
gaaggtgaaggcgatgcaaccaatggtaaactgacgctgaagtttatttgcaccacgggtaaactgccggttccgtggccgaccctggtcacc
acgctgacgtatggtgttcagtgtttcagtcgttacccggatcacatgaaacgccacgactttttcaagtccgcgatgccggaaggttatgtccaa
gaacgtaccatctcatttaaagatgacggcacctacaaaacgcgcgccgaagtgaaattcgaaggtgatacgctggttaaccgtattgaactg
aaaggcatcgattttaaggaagacggtaatattctgggccataaactggaatataacttcaattcgcacaacgtgtacatcaccgcagataag
cagaagaacggtatcaaggctaacttcaagatccgccataatgtggaagatggcagcgttcaactggccgaccactatcagcaaaacaccc
cgattggtgatggcccggtcctgctgccggacaatcattacctgagcacgcagtctgtgctgagtaaagatccgaacgaaaagcgtgaccac
atggtcctgctggaattcgtgaccgcggccggcatcacgcacggtatggacgaactgtataaaggctcagagctccatcaccatcaccatcac
taa

*Mm*PylRS:
MDKKPLNTLISATGLWMSRTGTIHKIKHHEVSRSKIYIEMACGDHLVVNNSRSSRTARAL**R**H**H**
KYRKTCKRCRVSDEDLNKFLTKANEDQTSVKVKVVSAPTRTKKAMPKSVARAPKPLENTEA
AQAQPSGSKFSPAIPVSTQESVSVPASVSTSISSISTGATASALVKGNTNPITSMSAPVQASAPAL
TK**S**QTDRLEVLLNPKDEISLNSGKPFRELESELLSRRKKDLQQIYAEERENYLGKLEREITRFF
VDRGFLEIKSPILIPLEYIERMGIDNDTELSKQIFRVDKNFCLRPMLAPNLYNYLRKLDRALPDP
IKIFEIGPCYRKESDGKEHLEEFTMLNFCQMGSGCTRENLESIITDFLNHLGIDFKIVGDSCMV
YGDTLDVMHGDLELSSAVVGPIPLDREWGIDKPWIGAGFGLERLLKVKHDFKNIKRAARSES
YYNGISTNL

R61/H63/S193 are labeled in bold type; P149 and A150, the junction for insertion of linker, are labelled in red.

sfGFP:
MXKGEELFTGVVPILVELDGDVNGHKXSVRGEGEGDATNGKLTLKFICTTGKLPVPWPTLVT
TLTYGVQCFSRYPDHMKRHDFFKSAMPEGYVQERTISFKDDGTYKTRAEVKFEGDTLVNRIE
LKGIDFKEDGNILGHKLEYNFNSHNVYITADKQKNGIKANFKIRHNVEDGSVQLADHYQQNT
PIGDGPVLLPDNHYLSTQSVLSKDPNEKRDHMVLLEFVTAAGITHGMDELYKGSELHHHHHH

X represents the positions for ncAA incorporation at S2 or F27 position with amber mutations.

2. Primers list

Primers	Primer sequence (5′ to 3′)
sfGFP-UAG2-NdeI-F	GATATACATATGTAGAAGGGCGAAGAACTGTTTACGGGCG
sfGFP-NdeI-F1	GAGATATACATATGAGCAAGGGCGAAG
sfGFP-UAG27-F	GGTCACAAATAGAGCGTGCGCGGCGAAGGTG
sfGFP-UAG27-R	CACCTTCGCCGCGCACGCTCTATTTGTGACC
sfGFP-SacI-R2	GATGGTGATGGAGCTCTGAGCCTTTATAC
PylRS-NcoI-F	GTATTAACCATGGATAAAAAACCACTAAAC
PylRS-EcoRI-R	GGCTTGCCGGAATTCAGGGAAATCTCATCTTTTG
PylRS-BamHI-R	GGTCGACGGATCCTTACAGGTTGGTAGAAATCCCGTTATAG
PylRS-EcoRI-F	GATTTCCCTGAATTCCGGCAAGCCTTTCAGGGAGC
PylRS-R61K/H63Y-F	GCAGGACTGCAAGAGCGCTCAAACACTATAAATACAGGAAG
PylRS-R61K/H63Y-R	TTTGCAGGTCTTCCTGTATTTATAGTGTTTGAGCGCTCTTG
PylRS-R61K-F	CAAGAGCGCTCAAACACCACAAATAC
PylRS-R61K-R	GTATTTGTGGTGTTTGAGCGCTCTTG
PylRS-H63Y-F	GCTCAGGCACTATAAATACAGGAAG
PylRS-H63Y-R	CTTCCTGTATTTATAGTGCCTGAGC
PylRS-S193R-F	CAGGCAAGTGCCCCCGCACTTACGAAGCGTCAGACTGACAG
PylRS-S193R-R	GACTTCAAGCCTGTCAGTCTGACGCTTCGTAAGTGCG
PylRS-P149SSS-R1	GTTGAAACAGATGCCATCGCTTTTTTCACGGGACAGAAAC
PylRS-P149SSS-F2	GTTTCTGTCCCGTGAAAAAAGCGATGGCATCTGTTTCAAC
PylRS-P149-TAA-BamHI-R	GGTCGACGGATCCTTACGGGACAGAAACTGACTC
Linker-1XG4S-F2	GAGTGGTGGTGGTGGTAGCGCATCTGTTTCAACATCAATATC
Linker-1XG4S-R1	GCTACCACCACCACCACTCGGGACAGAAACTGACTCTTGGGTG
Linker-2XG4S-F2	CAAGAGTCAGTTTCTGTCCCGAGTGGTGGTGGTGGTAGCGGAGG
Linker-2XG4S-F3	CAACATCAATATCAAGCATTTCTACAGGAGCAAC
Linker-2XG4S-R1	GACAGAAACTGACTCTTGGGTGGAAACCGGTATC
Linker-2XG4S-R2	CTTGATATTGATGTTGAAACAGATGCGCTTCCTCCCCCTCCGCTACCAC
Linker-3XG4S-F2	GTCAGTTTCTGTCCCGAGTGGTGGTGGTGGTAGCGGAGGGGGAGGAAG
Linker-3XG4S-F3	CAACATCAATATCAAGCATTTCTACAGGAGCAAC
Linker-3XG4S-R1	GACAGAAACTGACTCTTGGGTGGAAACCGGTATC
Linker-3XG4S-R2	GATATTGATGTTGAAACAGATGCGCTTCCTCCCCCTCCGCTTCCTCCCCCTC

3. Supplementary Figures

(A)

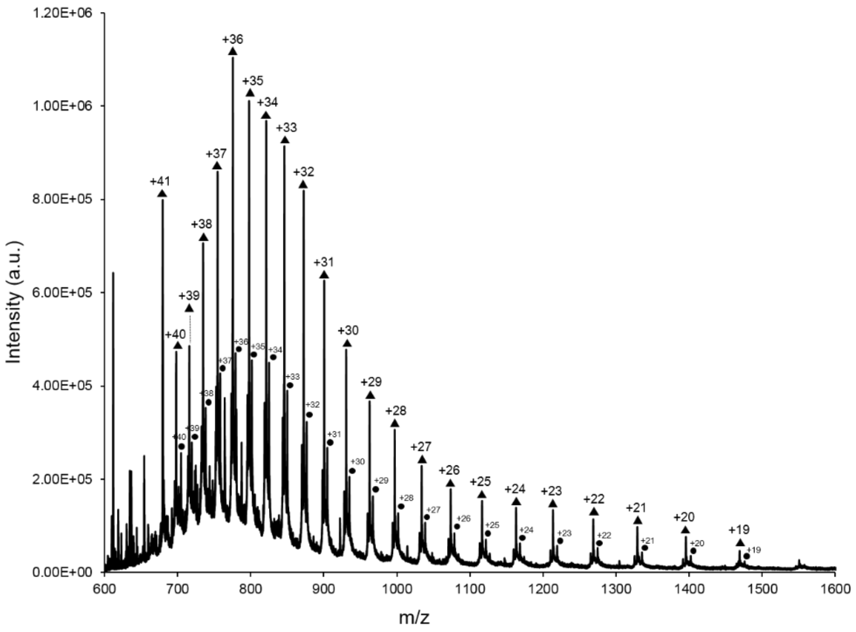

(B)

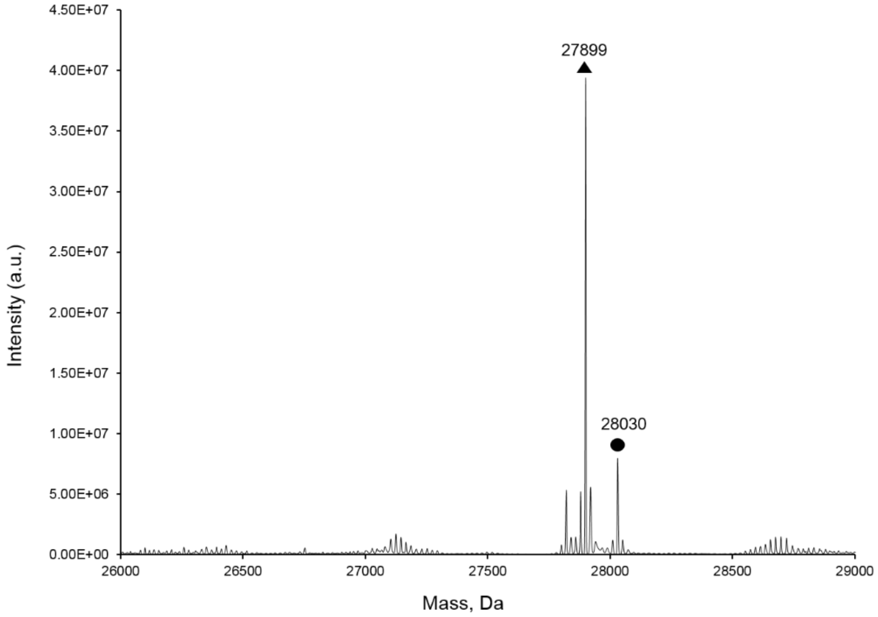

Figure S1. Molecular mass determination of sfGFP-3.

Full-length sfGFP-UAG27-**3** was expressed using N-PylRS•tRNA$^{Pyl}_{CUA}$ pair in *E. coli* BL21 (DE3) with the supplement of 2 mM **3** in GMML medium. (A) ESI-MS spectrum of sfGFP-UAG27-**3**. (B) The deconvoluted ESI-MS spectrum of sfGFP-UAG27-**3**. The calculated molecular masses are 28,030 Da and 27,899 Da (–Met); observed molecular masses are 28,030 Da and 27,899 Da (–Met).

(A)

(B)

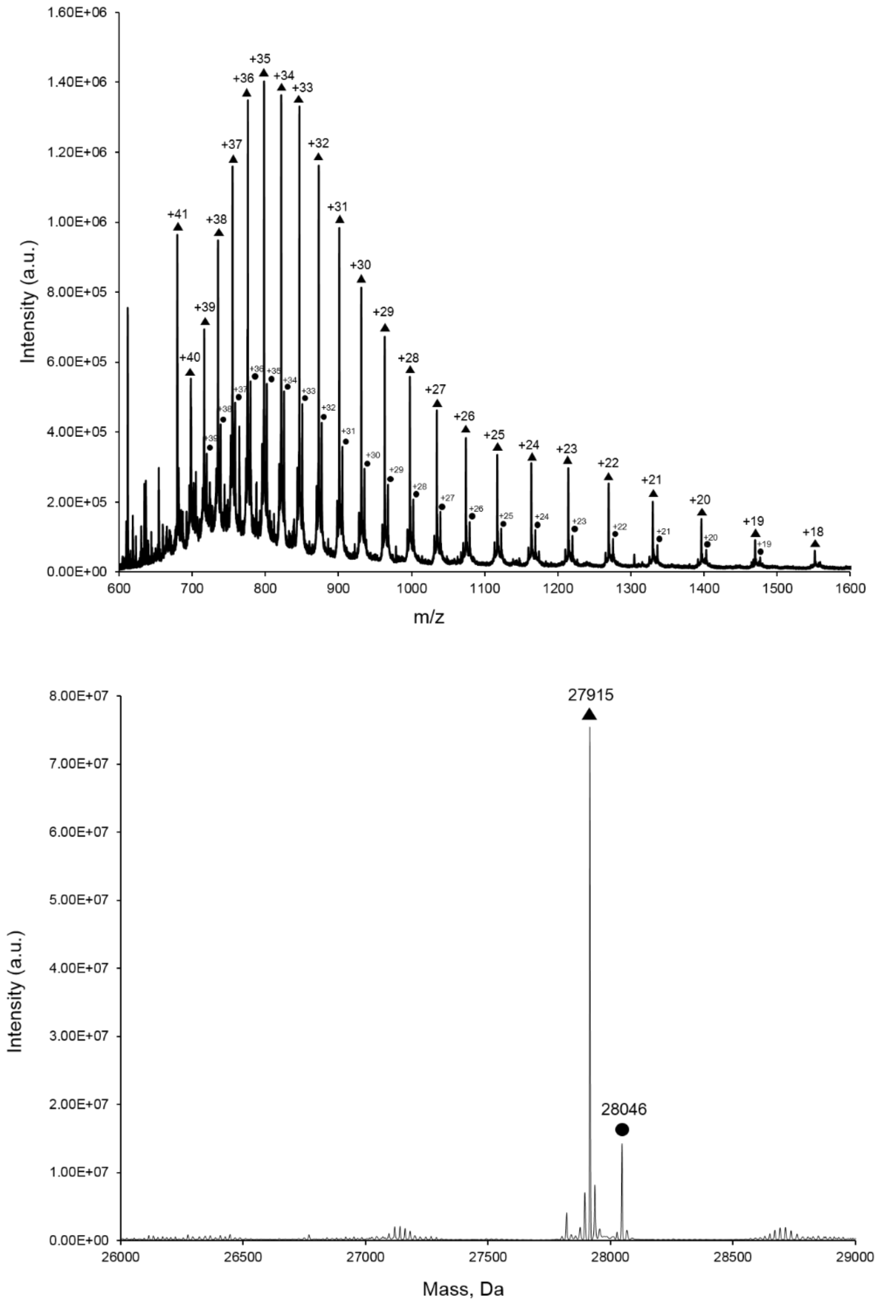

Figure S2. Molecular mass determination of sfGFP-4.

Full-length sfGFP-UAG27-**4** was expressed using N-PylRS•tRNA$^{Pyl}_{CUA}$ pair in *E. coli* BL21 (DE3) with the supplement of 2 mM **4** in GMML medium. (A) ESI-MS spectrum of sfGFP-UAG27-**4**. (B) The deconvoluted ESI-MS spectrum of sfGFP-UAG27-**4**. The calculated molecular masses are 28,046 Da and 27,915 Da (–Met); observed molecular masses are 28,046 Da and 27,915 Da (–Met).

(A)

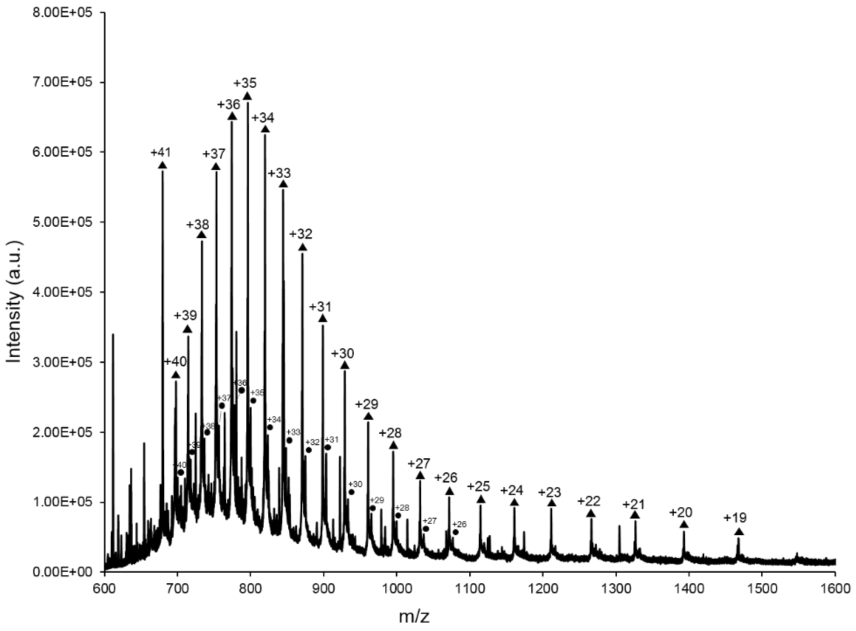

(B)

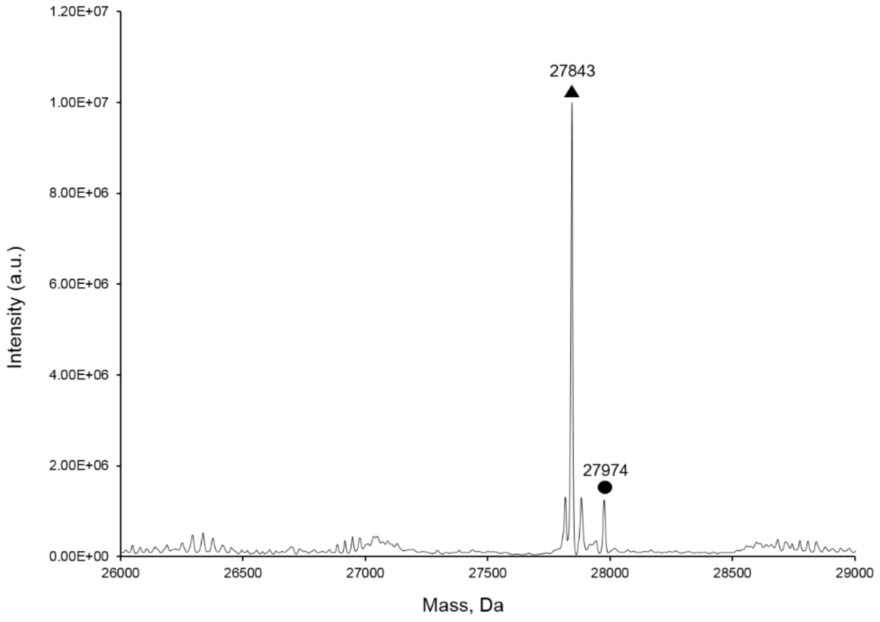

Figure S3. Molecular mass determination of sfGFP-5.

Full-length sfGFP-UAG27-**5** was expressed using N-PylRS•$tRNA^{Pyl}_{CUA}$ pair in *E. coli* BL21 (DE3) with the supplement of 1 mM **5** in LB medium. (A) ESI-MS spectrum of sfGFP-UAG27-**5**. (B) The deconvoluted ESI-MS spectrum of sfGFP-UAG27-**5**. The calculated molecular masses are 27,974 Da and 27,843 Da (–Met); observed molecular masses are 27,974 Da and 27,843 Da (–Met).

(A)

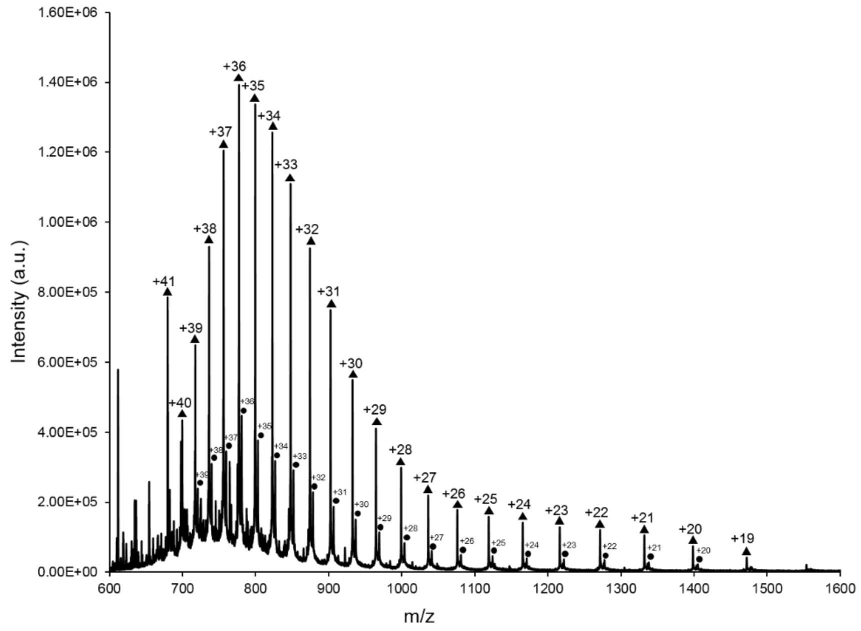

(B)

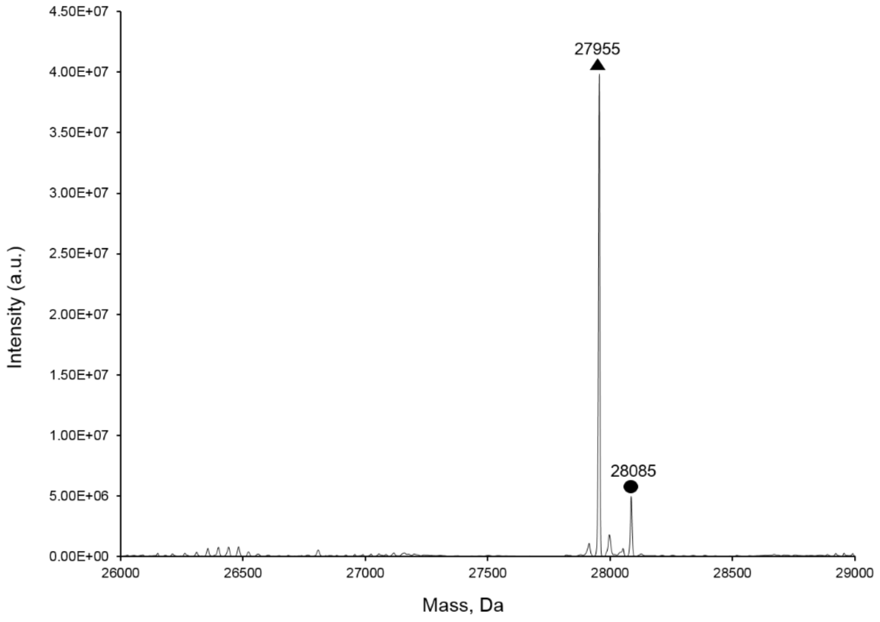

Figure S4. Molecular mass determination of sfGFP-6.

Full-length sfGFP-UAG27-**6** was expressed using N-ZRS•$\text{tRNA}^{\text{Pyl}}_{\text{CUA}}$ pair in *E. coli* BL21 (DE3) with the supplement of 1 mM **6** in LB medium. (A) ESI-MS spectrum of sfGFP-UAG27-**6**. (B) The deconvoluted ESI-MS spectrum of sfGFP-UAG27-**6**. The calculated molecular masses are 28,085 Da and 27,954 Da (–Met); observed molecular masses are 28,085 Da and 27,955 Da (–Met).

(A)

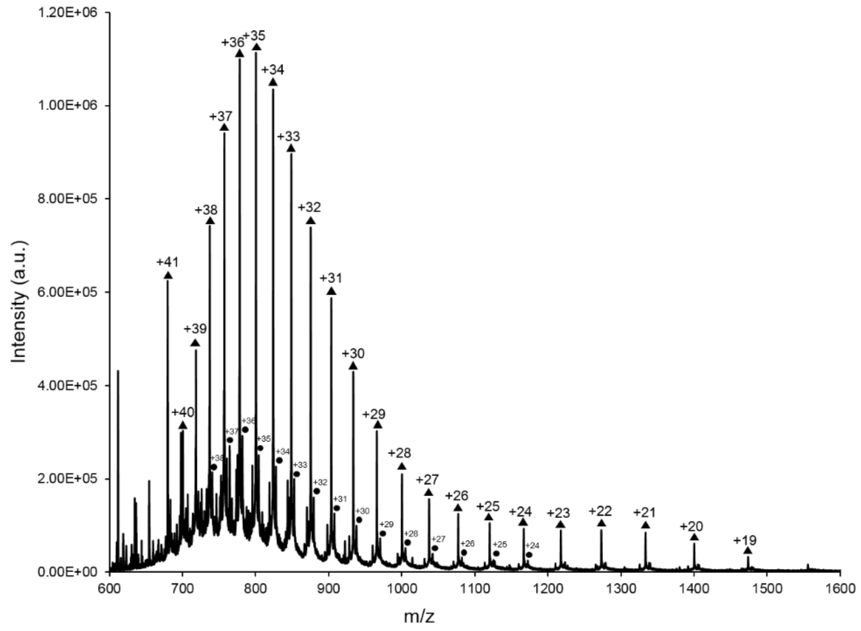

(B)

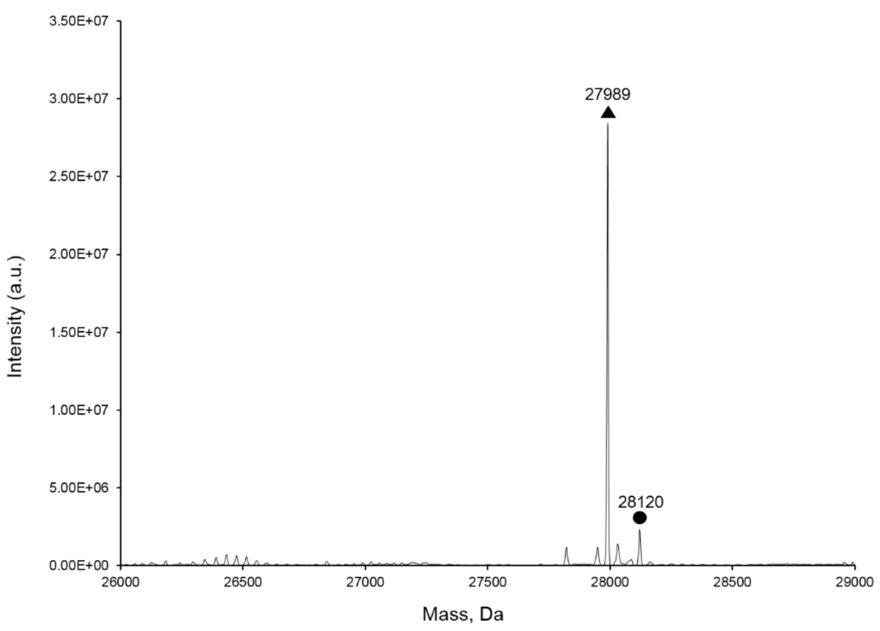

Figure S5. Molecular mass determination of sfGFP-7.

Full-length sfGFP-UAG27-**7** was expressed using N-ZRS•tRNA$^{Pyl}_{CUA}$ pair in *E. coli* BL21 (DE3) with the supplement of 1 mM **7** in LB medium. (A) ESI-MS spectrum of sfGFP-UAG27-**7**. (B) The deconvoluted ESI-MS spectrum of sfGFP-UAG27-**7**. The calculated molecular masses are 28,120 Da and 27,988 Da (–Met); observed molecular masses are 28,120 Da and 27,989 Da (–Met).

(A)

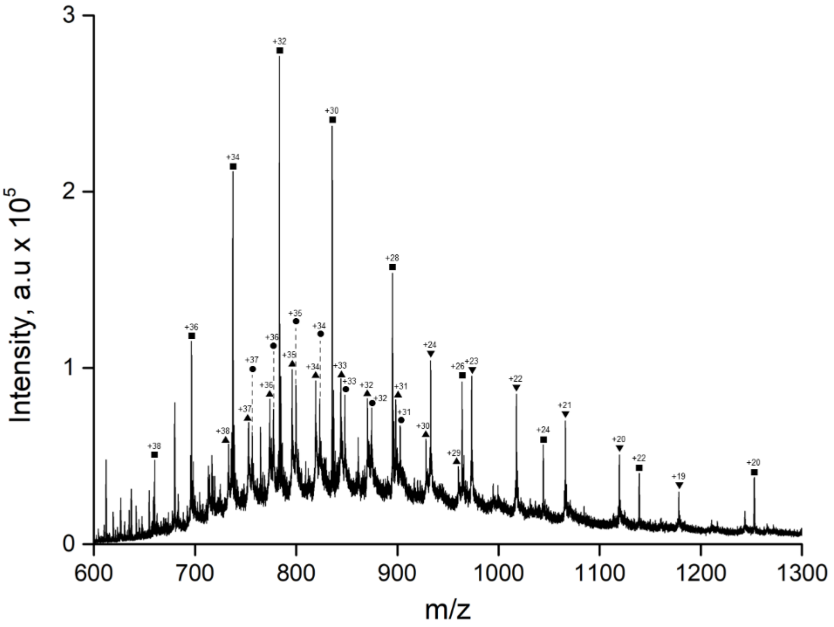

(B)

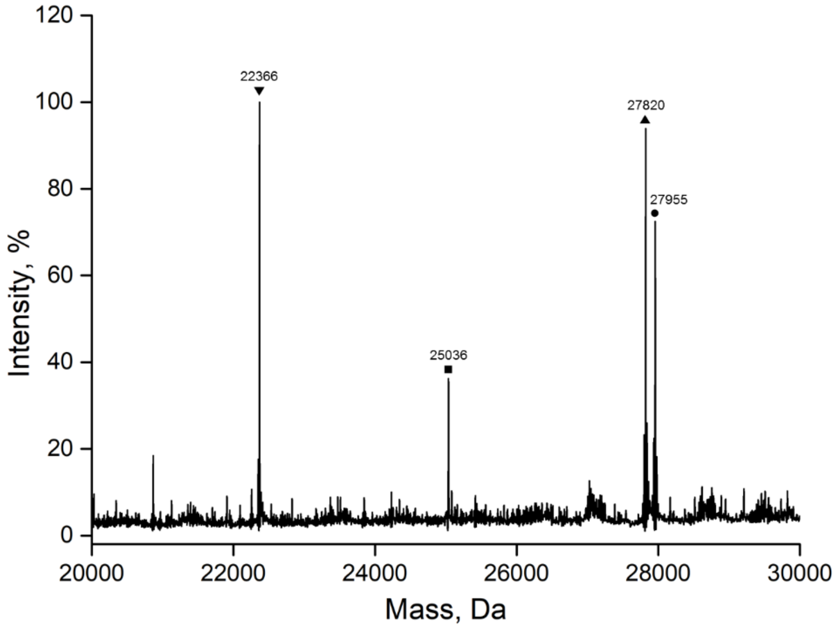

Figure S6. Molecular mass determination of sfGFP-11.

Full-length sfGFP-UAG27-**11** was expressed using N-ZRS•tRNA$^{Pyl}_{CUA}$ pair in *E. coli* BL21 (DE3) with the supplement of 1 mM **11** in GMML medium. (A) ESI-MS spectrum of sfGFP-UAG27-**11**. (B) The deconvoluted ESI-MS spectrum of sfGFP-UAG27-**11**. The calculated molecular masses of sfGFP-UAG27-**11** are 28,086 Da, 27,955 Da (–Met), 25,319 Da (truncated sfGFP at position 27), and 25,057 Da (truncated sfGFP at 27 position without N-terminal CbzKOH); observed molecular masses are 27,955 Da (–Met) and 27,820 Da (without Cbz group at position 27 and N-terminal Met residue), 25,036 Da, and 22,366 Da.

(A)

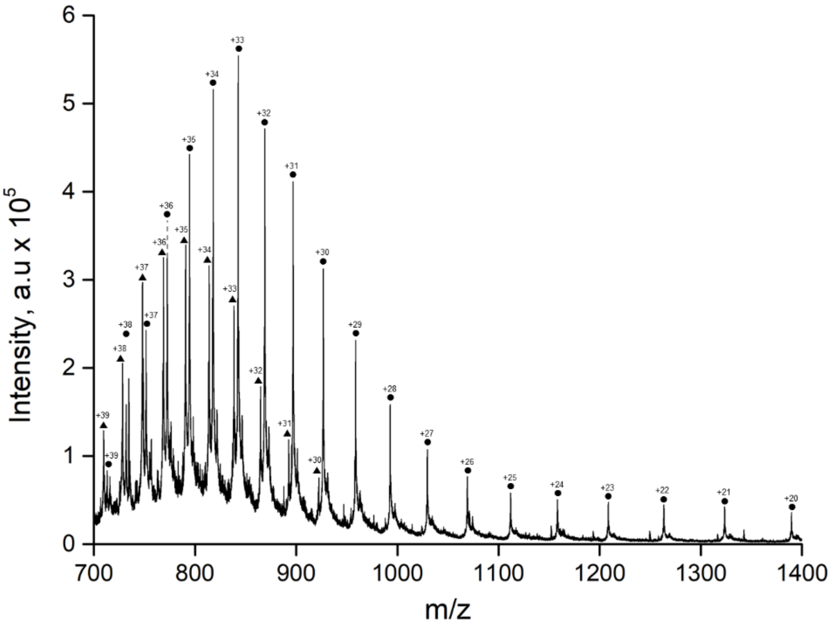

(B)

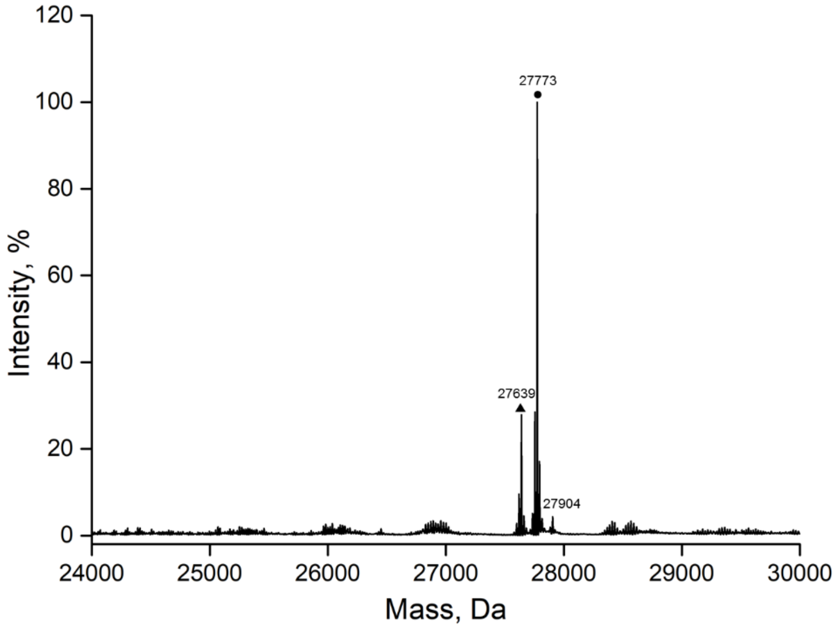

Figure S7. Molecular mass determination of sfGFP-11*.

Full-length sfGFP-UAG2-**11** was expressed using ZRS-D1•$tRNA^{Pyl}_{CUA}$ pair in *E. coli* BL21 (DE3) with the supplement of 1 mM **11** in GMML medium. (A) ESI-MS spectrum of sfGFP-UAG2-**11**. (B) The deconvoluted ESI-MS spectrum of sfGFP-UAG2-**11**. The calculated molecular masses of sfGFP-UAG2-**11** are 27,904 Da, 27,773 Da (–Met), and 27,639 Da (without Cbz group at 2 position and N-terminal Met residue); observed molecular masses are 27,904 Da, 27,773 Da (–Met) and 27,639 Da.

(A)

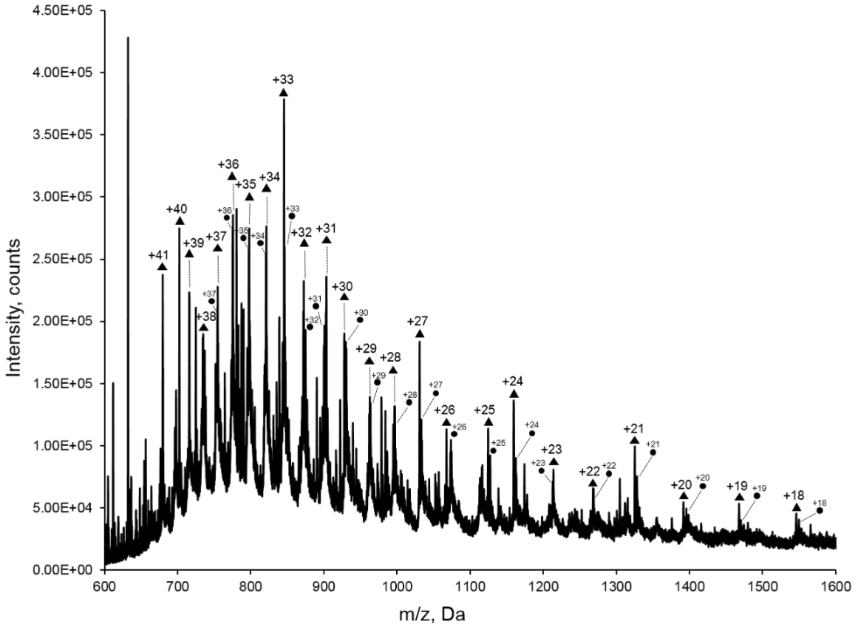

(B)

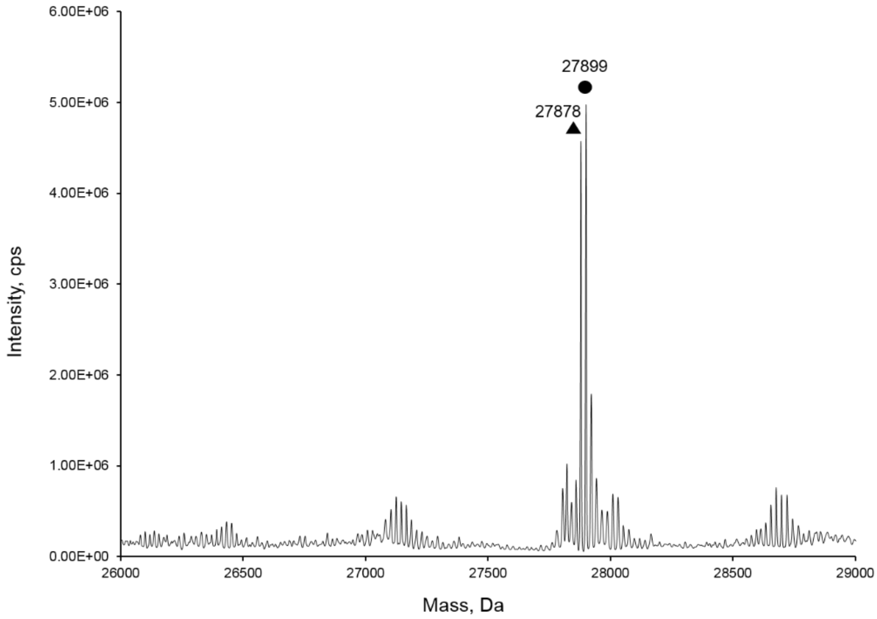

Figure S8. Molecular mass determination of sfGFP-3.

Full-length sfGFP-UAG27-**3** was expressed using N-ZRS•$tRNA^{Pyl}_{CUA}$ pair in *E. coli* BL21 (DE3) with the supplement of 2 mM **3** in GMML medium. (A) ESI-MS spectrum of sfGFP-UAG27-**3**. (B) The deconvoluted ESI-MS spectrum of sfGFP-UAG27-**3**. The calculated molecular masses are 28,030 Da and 27,899 Da (–Met); observed molecular masses are 27,878 Da and 27,899 Da (–Met). The calculated molecular mass of sfGFP-F27W (–Met) is 27,878 Da.

(A)

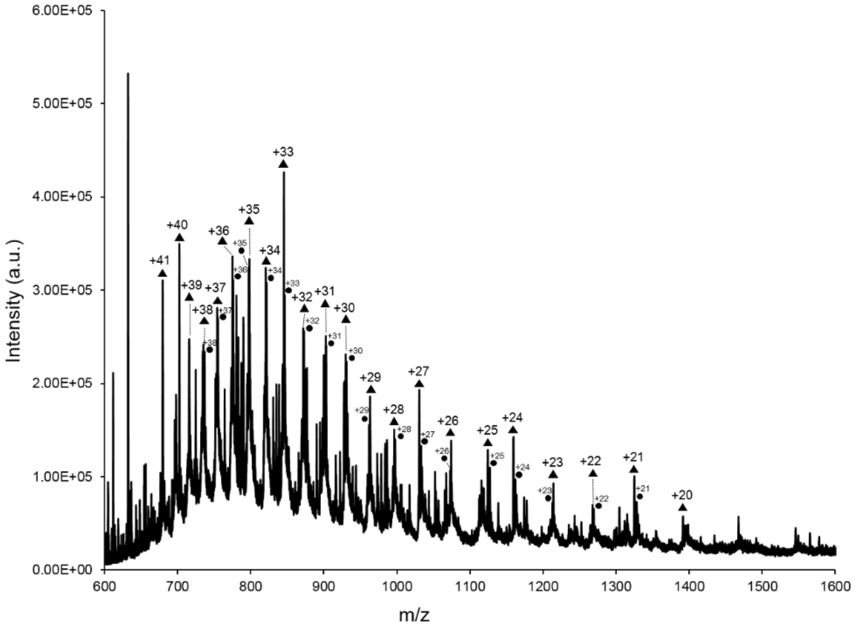

(B)

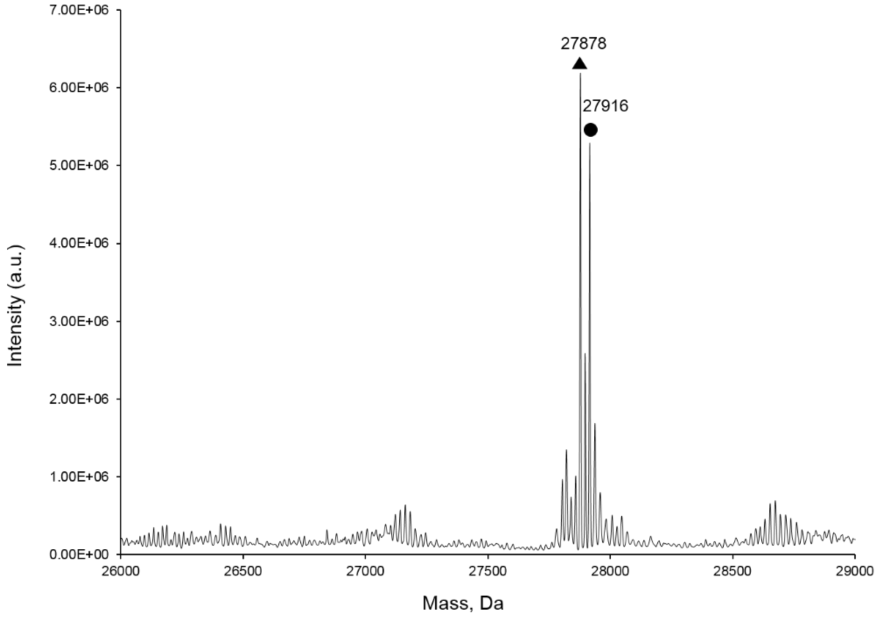

Figure S9. Molecular mass determination of sfGFP-4.

Full-length sfGFP-UAG27-**4** was expressed using N-ZRS•$tRNA^{Pyl}_{CUA}$ pair in *E. coli* BL21 (DE3) with the supplement of 2 mM **4** in GMML medium. (A) ESI-MS spectrum of sfGFP-UAG27-**4**. (B) The deconvoluted ESI-MS spectrum of sfGFP-UAG27-**4**. The calculated molecular masses are 28,046 Da and 27,915 Da (–Met); observed molecular masses are 27,878 Da and 27,916 Da (–Met). The calculated molecular mass of sfGFP-F27W (–Met) is 27,878 Da.

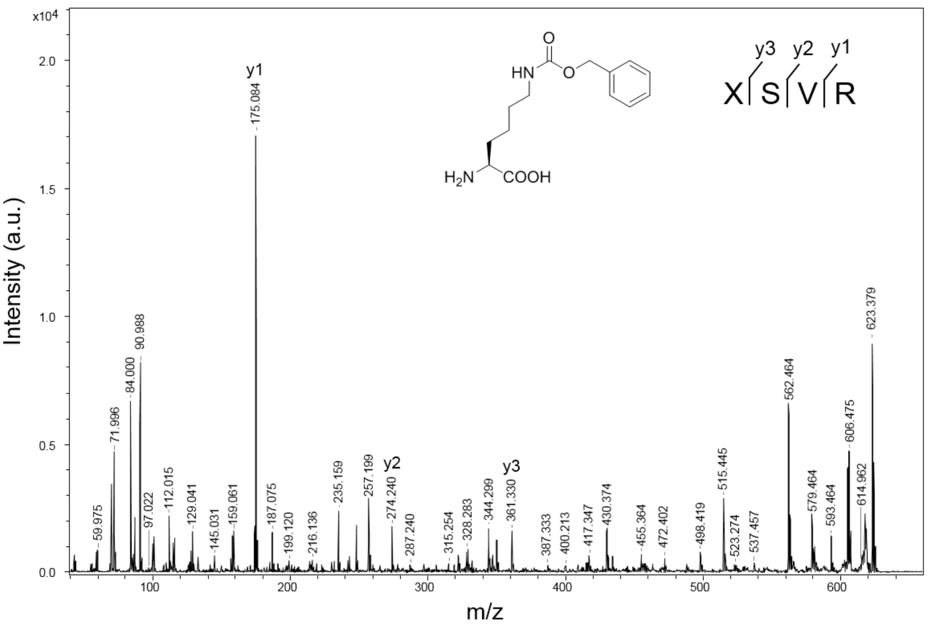

Figure S10. MALDI-TOF-MS/MS analysis of sfGFP-6 at position 27.
Full-length sfGFP-**6** was expressed using N-ZRS in the presence of 1 mM **6** in LB medium. The MS/MS spectrum of the XSVR; X denotes ncAA **6** incorporated at position 27 fragment from sfGFP-**6**. The protein was in-gel digested by trypsin. The calculated molecular mass of X(**6**)SVR peptide fragment is 622.344 Da and found molecular mass is 623.379 Da.

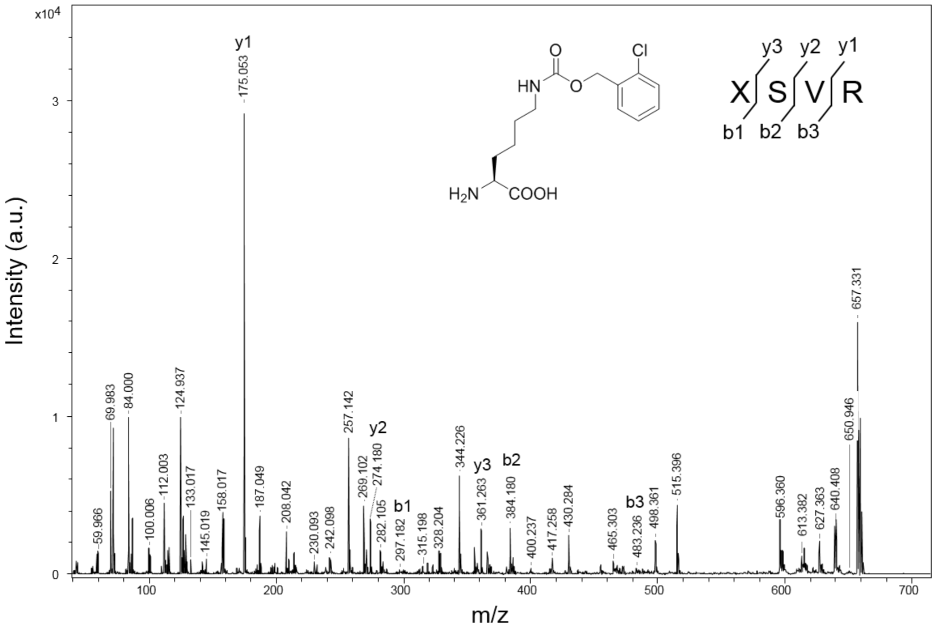

Figure S11. MALDI-TOF-MS/MS analysis of sfGFP-7 at position 27.
Full-length sfGFP-**7** was expressed using N-ZRS in the presence of 1 mM **7** in LB medium. The MS/MS spectrum of the XSVR; X denotes ncAA **7** incorporated at position 27 fragment from sfGFP-**7**. The protein was in-gel digested by trypsin. The calculated molecular mass of X(**7**)SVR peptide fragment is 656.305 Da and found molecular mass is 657.331 Da.

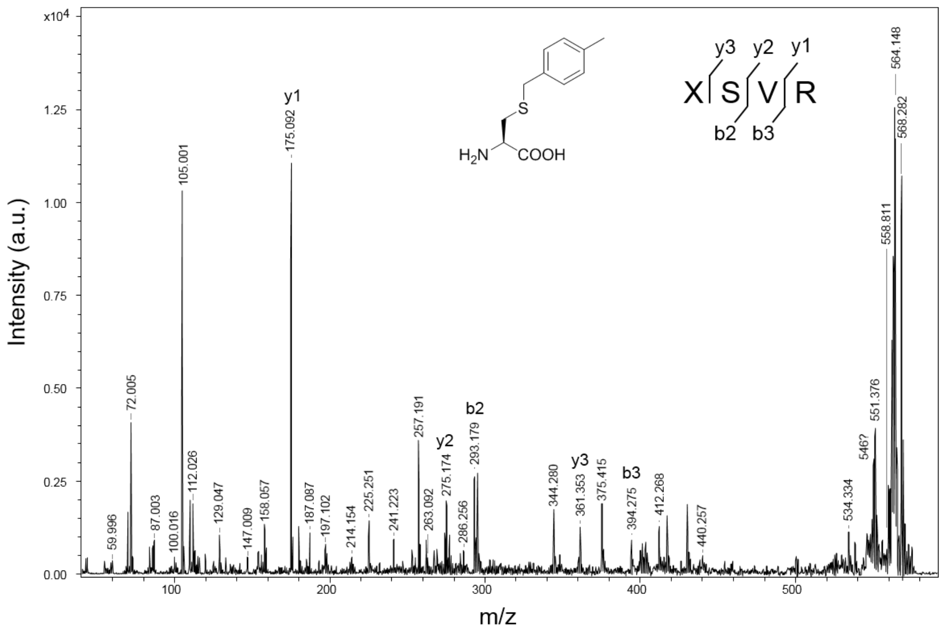

Figure S12. MALDI-TOF-MS/MS analysis of sfGFP-3 at position 27.
Full-length sfGFP-**3** was expressed using N-ZRS in the presence of 2 mM **3** in GMML medium. The MS/MS spectrum of the XSVR; X denotes ncAA **3** incorporated at position 27 fragment from sfGFP-**3**. The protein was in-gel digested by trypsin. The calculated molecular mass of X(**3**)SVR peptide fragment is 567.284 Da and found molecular mass is 568.282 Da.

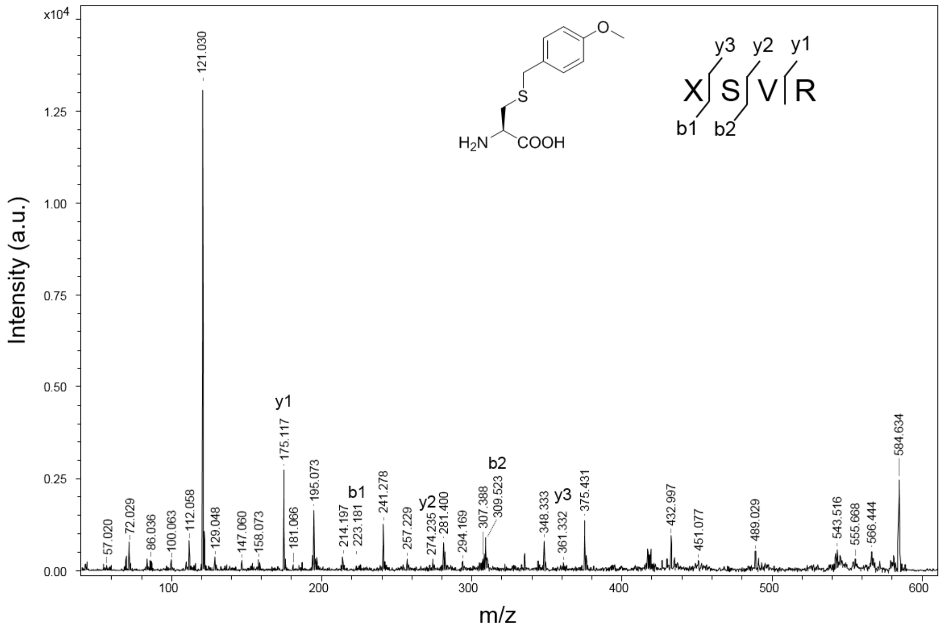

Figure S13. MALDI-TOF-MS/MS analysis of sfGFP-4 at position 27.
Full-length sfGFP-**4** was expressed using N-ZRS in the presence of 2 mM **4** in GMML medium. The MS/MS spectrum of the XSVR; X denotes ncAA **4** incorporated at position 27 fragment from sfGFP-**4**. The protein was in-gel digested by trypsin. The calculated molecular mass of X(**4**)SVR peptide fragment is 583.279 Da and found molecular mass is 584.634 Da.

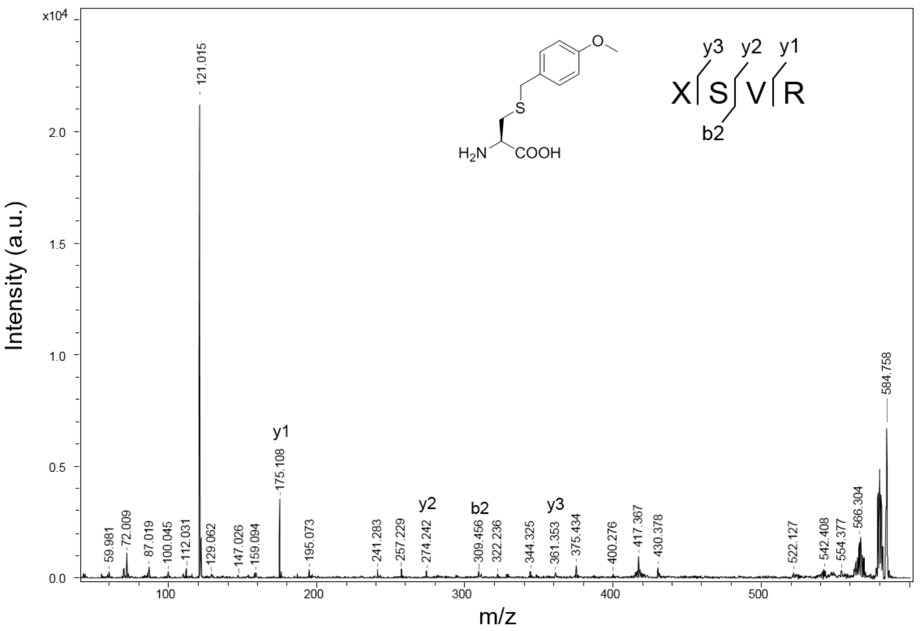

Figure S14. MALDI-TOF-MS/MS analysis of sfGFP-4 at position 27.
Full-length sfGFP-**4** was expressed using N-PylRS in the presence of 2 mM **4** in GMML medium. The MS/MS spectrum of the XSVR; X denotes ncAA **4** incorporated at position 27 fragment from sfGFP-**4**. The protein was in-gel digested by trypsin. The calculated molecular mass of X(**4**)SVR peptide fragment is 583.279 Da and found molecular mass is 584.758 Da.

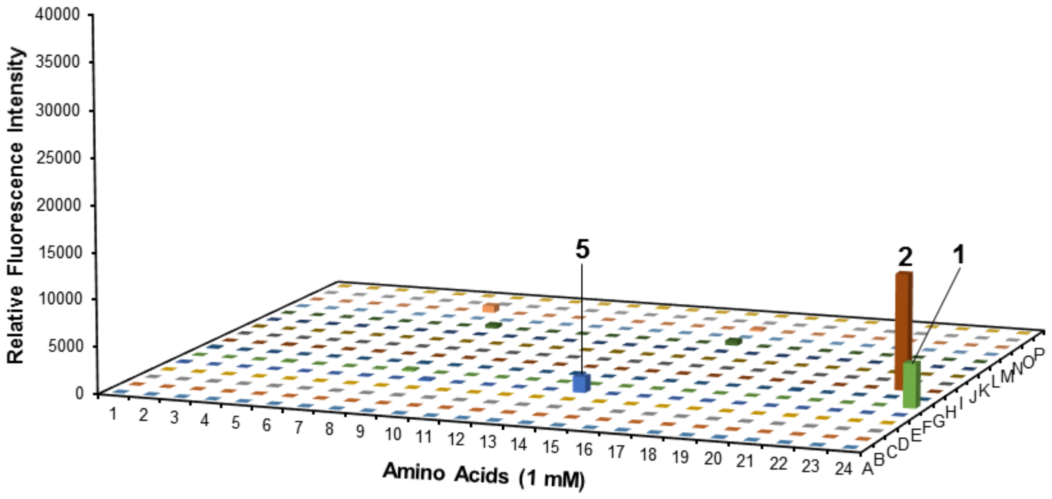

Figure S15. Substrate range of wt-PylRS•tRNA$^{Pyl}_{CUA}$ pair in producing sfGFP-UAG27 proteins. *sfGFP-UAG27* gene read-through in ncAAs library was measured by fluorescence intensity. Twelve wells (A1-2, B1-2, and C1-2: without ncAAs and IPTG; D1-2, E1-2, and F1-2: without ncAAs but containing IPTG) were used as controls to measure background signals. Substrate specificity profiles of wt-PylRS•tRNA$^{Pyl}_{CUA}$ pair were observed by *sfGFP-UAG27* gene suppression. The experiments were performed in the presence of 1 mM ncAAs and IPTG in GMML medium at 37℃ for 12 h. *E. coli* BL21 (DE3) was used in the experiment.

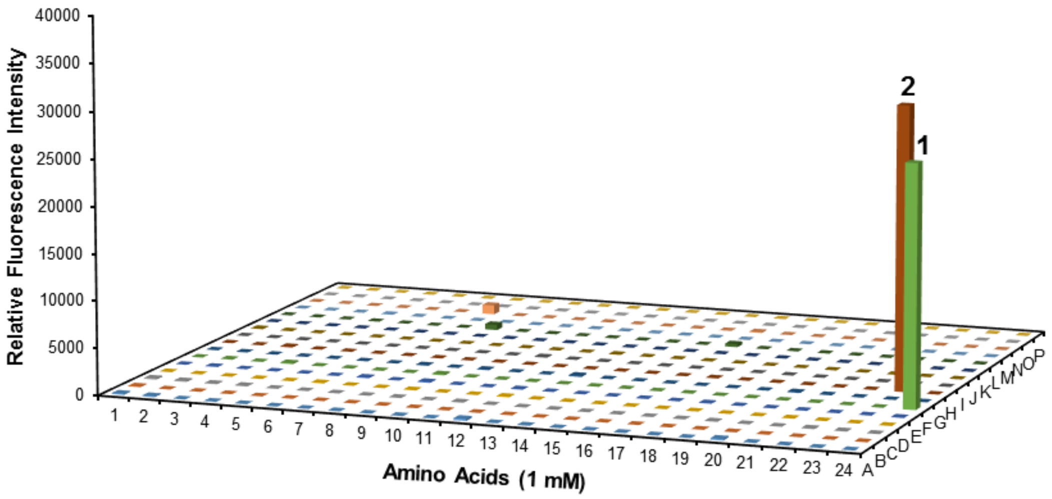

Figure S16. Substrate range of wt-PylRS•tRNA$^{Pyl}_{CUA}$ pair in producing sfGFP-UAG2 proteins. *sfGFP-UAG2* gene read-through in ncAAs library was measured by fluorescence intensity. Twelve wells (A1-2, B1-2, and C1-2: without ncAAs and IPTG; D1-2, E1-2, and F1-2: without ncAAs but containing IPTG) were used as controls to measure background signals. Substrate specificity profiles of wt-PylRS•tRNA$^{Pyl}_{CUA}$ pair were observed by *sfGFP-UAG2* gene suppression. The experiments were performed in the presence of 1 mM ncAAs and IPTG in GMML medium at 37℃ for 12 h. *E. coli* BL21 (DE3) was used in the experiment.

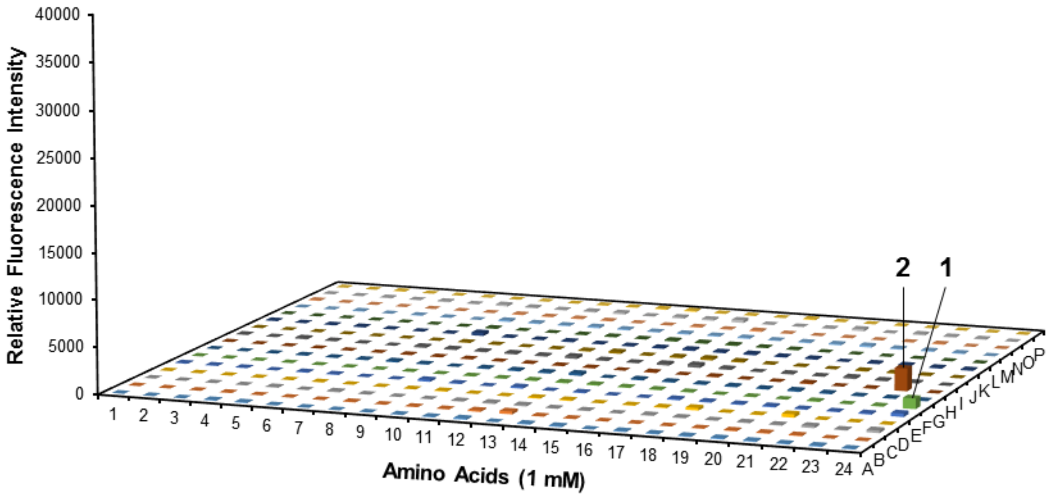

Figure S17. Substrate range of PylRS-D1•tRNA$^{\text{Pyl}}_{\text{CUA}}$ pair in producing sfGFP-UAG27 proteins. *sfGFP-UAG27* gene read-through in ncAAs library was measured by fluorescence intensity. Twelve wells (A1-2, B1-2, and C1-2: without ncAAs and IPTG; D1-2, E1-2, and F1-2: without ncAAs but containing IPTG) were used as controls to measure background signals. Substrate specificity profiles of PylRS-D1•tRNA$^{\text{Pyl}}_{\text{CUA}}$ pair were observed by *sfGFP-UAG27* gene suppression. The experiments were performed in the presence of 1 mM ncAAs and IPTG in GMML medium at 37℃ for 12 h. *E. coli* BL21 (DE3) was used in the experiment.

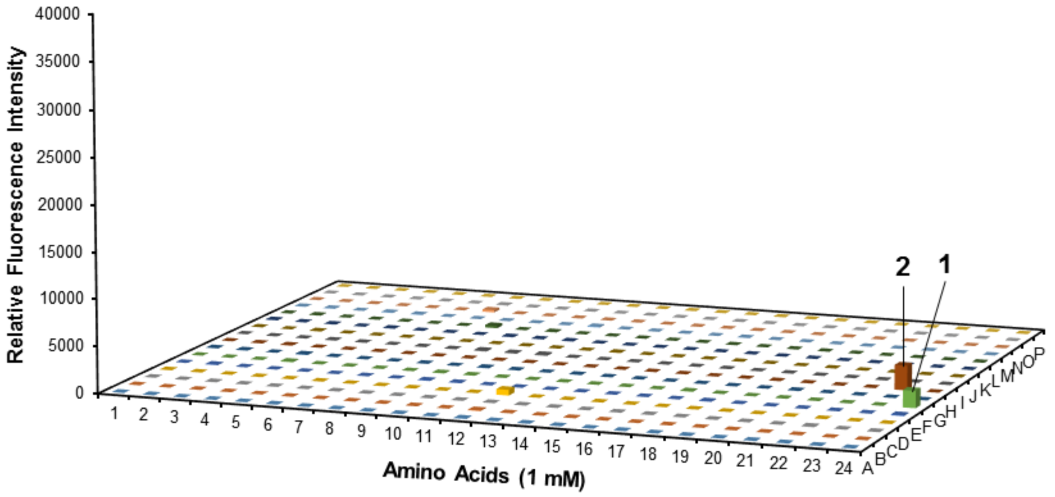

Figure S18. Substrate range of PylRS-D1•tRNA$^{\text{Pyl}}_{\text{CUA}}$ pair in producing sfGFP-UAG2 proteins. *sfGFP-UAG2* gene read-through in ncAAs library was measured by fluorescence intensity. Twelve wells (A1-2, B1-2, and C1-2: without ncAAs and IPTG; D1-2, E1-2, and F1-2: without ncAAs but containing IPTG) were used as controls to measure background signals. Substrate specificity profiles of PylRS-D1•tRNA$^{\text{Pyl}}_{\text{CUA}}$ pair were observed by *sfGFP-UAG2* gene suppression. The experiments were performed in the presence of 1 mM ncAAs and IPTG in GMML medium at 37℃ for 12 h. *E. coli* BL21 (DE3) was used in the experiment.

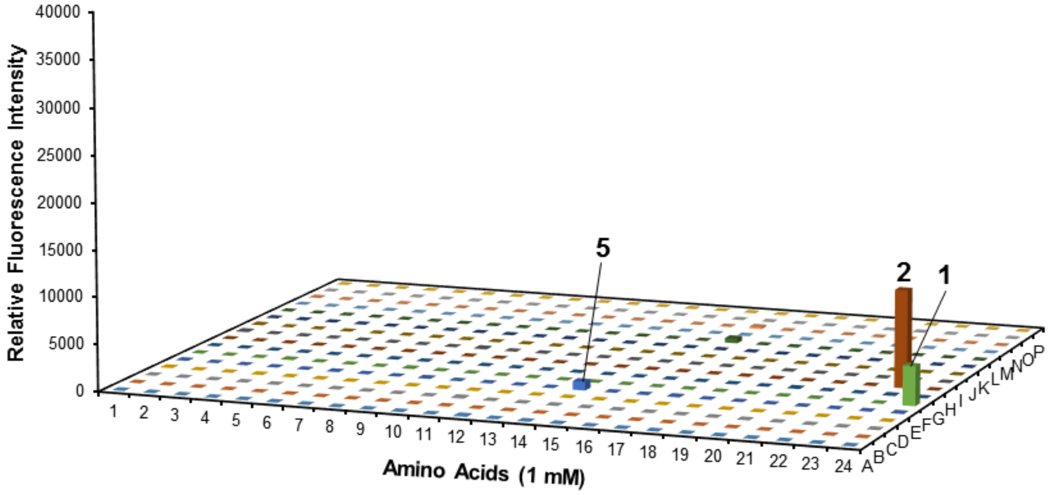

Figure S19. Substrate range of PylRS-L1•$tRNA^{Pyl}_{CUA}$ pair in producing sfGFP-UAG27 proteins. *sfGFP-UAG27* gene read-through in ncAAs library was measured by fluorescence intensity. Twelve wells (A1-2, B1-2, and C1-2: without ncAAs and IPTG; D1-2, E1-2, and F1-2: without ncAAs but containing IPTG) were used as controls to measure background signals. Substrate specificity profiles of PylRS-L1•$tRNA^{Pyl}_{CUA}$ pair were observed by *sfGFP-UAG27* gene suppression. The experiments were performed in the presence of 1 mM ncAAs and IPTG in GMML medium at 37℃ for 12 h. *E. coli* BL21 (DE3) was used in the experiment.

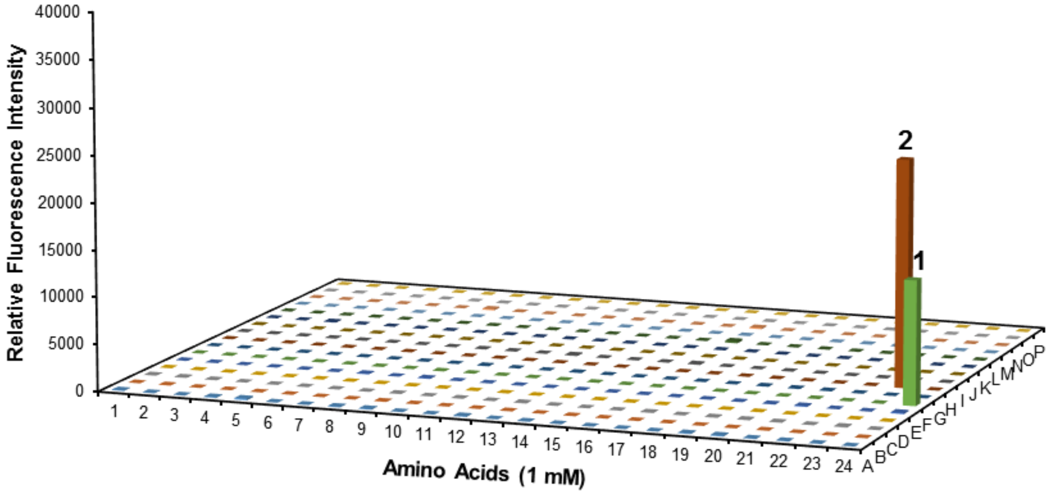

Figure S20. Substrate range of PylRS-L1•$tRNA^{Pyl}_{CUA}$ pair in producing sfGFP-UAG2 proteins. *sfGFP-UAG2* gene read-through in ncAAs library was measured by fluorescence intensity. Twelve wells (A1-2, B1-2, and C1-2: without ncAAs and IPTG; D1-2, E1-2, and F1-2: without ncAAs but containing IPTG) were used as controls to measure background signals. Substrate specificity profiles of PylRS-L1•$tRNA^{Pyl}_{CUA}$ pair were observed by *sfGFP-UAG2* gene suppression. The experiments were performed in the presence of 1 mM ncAAs and IPTG in GMML medium at 37℃ for 12 h. *E. coli* BL21 (DE3) was used in the experiment.

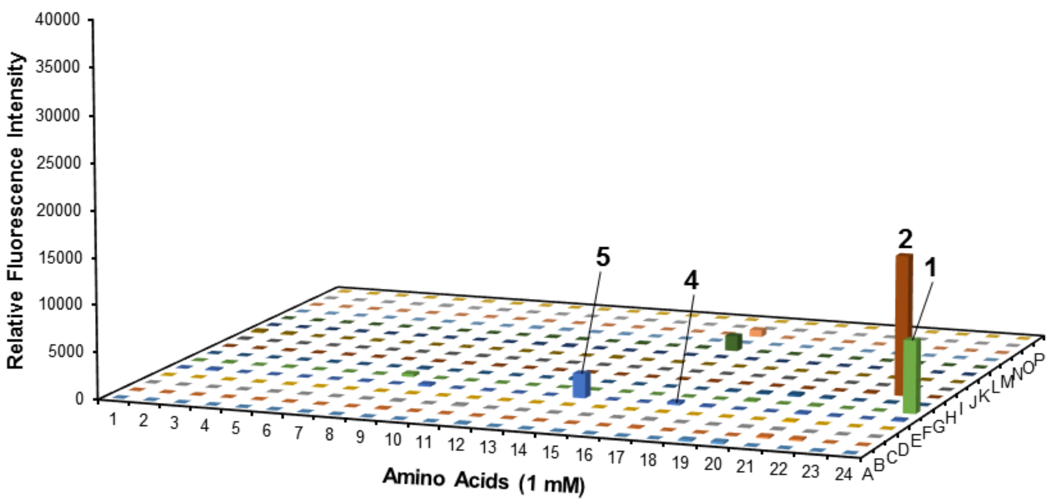

Figure S21. Substrate range of PylRS-L2•tRNA$^{Pyl}_{CUA}$ pair in producing sfGFP-UAG27 proteins. *sfGFP-UAG27* gene read-through in ncAAs library was measured by fluorescence intensity. Twelve wells (A1-2, B1-2, and C1-2: without ncAAs and IPTG; D1-2, E1-2, and F1-2: without ncAAs but containing IPTG) were used as controls to measure background signals. Substrate specificity profiles of PylRS-L2•tRNA$^{Pyl}_{CUA}$ pair were observed by *sfGFP-UAG27* gene suppression. The experiments were performed in the presence of 1 mM ncAAs and IPTG in GMML medium at 37℃ for 12 h. *E. coli* BL21 (DE3) was used in the experiment.

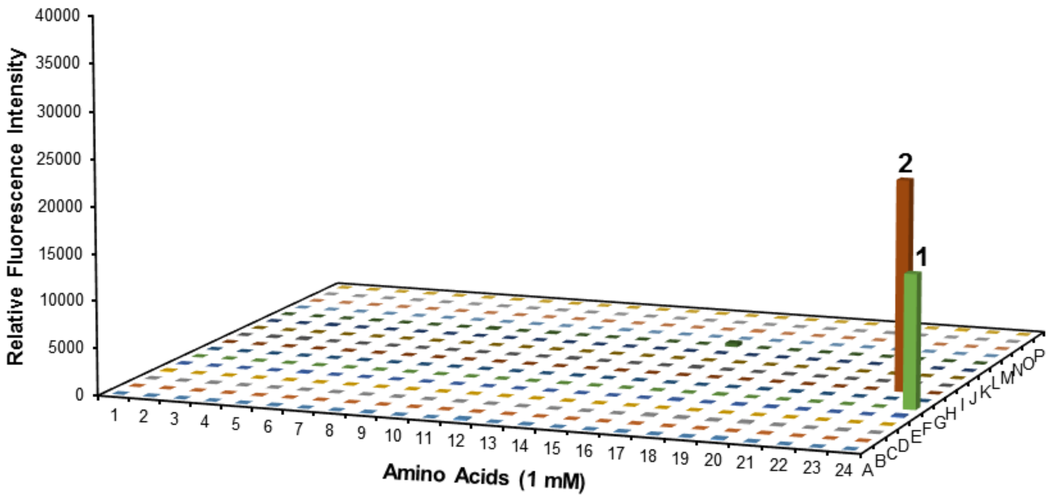

Figure S22. Substrate range of PylRS-L2•tRNA$^{Pyl}_{CUA}$ pair in producing sfGFP-UAG2 proteins. *sfGFP-UAG2* gene read-through in ncAAs library was measured by fluorescence intensity. Twelve wells (A1-2, B1-2, and C1-2: without ncAAs and IPTG; D1-2, E1-2, and F1-2: without ncAAs but containing IPTG) were used as controls to measure background signals. Substrate specificity profiles of PylRS-L2•tRNA$^{Pyl}_{CUA}$ pair were observed by *sfGFP-UAG2* gene suppression. The experiments were performed in the presence of 1 mM ncAAs and IPTG in GMML medium at 37℃ for 12 h. *E. coli* BL21 (DE3) was used in the experiment.

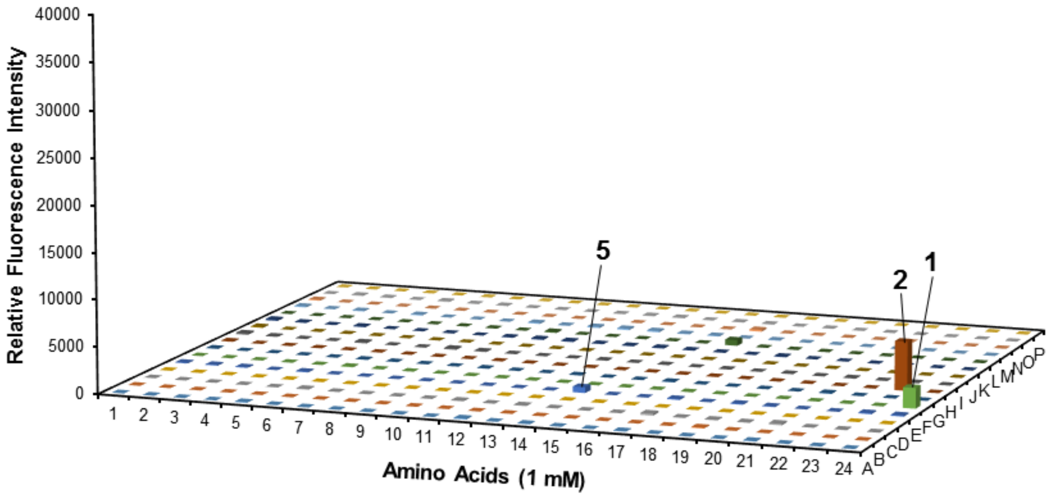

Figure S23. Substrate range of PylRS-L3•tRNA$^{Pyl}_{CUA}$ pair in producing sfGFP-UAG27 proteins. *sfGFP-UAG27* gene read-through in ncAAs library was measured by fluorescence intensity. Twelve wells (A1-2, B1-2, and C1-2: without ncAAs and IPTG; D1-2, E1-2, and F1-2: without ncAAs but containing IPTG) were used as controls to measure background signals. Substrate specificity profiles of PylRS-L3•tRNA$^{Pyl}_{CUA}$ pair were observed by *sfGFP-UAG27* gene suppression. The experiments were performed in the presence of 1 mM ncAAs and IPTG in GMML medium at 37℃ for 12 h. *E. coli* BL21 (DE3) was used in the experiment.

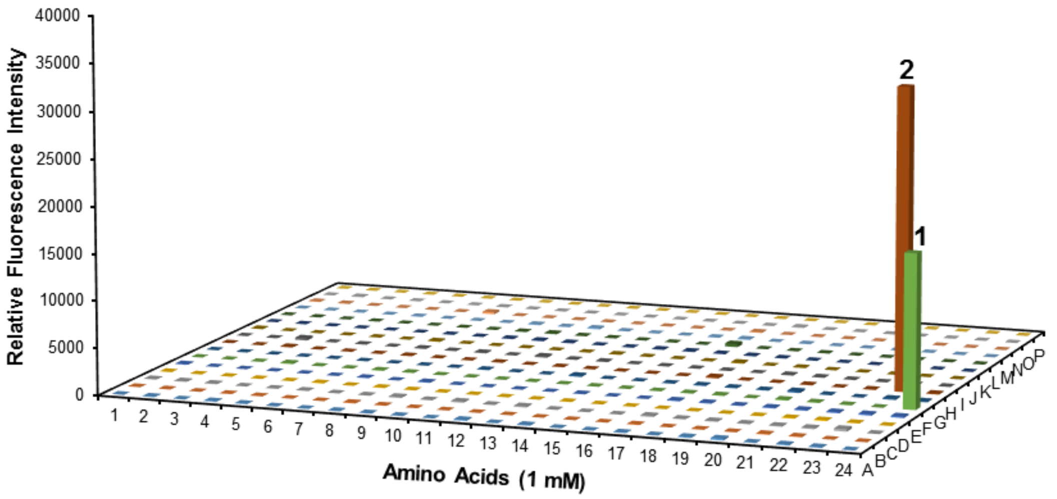

Figure S24. Substrate range of PylRS-L3•tRNA$^{Pyl}_{CUA}$ pair in producing sfGFP-UAG2 proteins. *sfGFP-UAG2* gene read-through in ncAAs library was measured by fluorescence intensity. Twelve wells (A1-2, B1-2, and C1-2: without ncAAs and IPTG; D1-2, E1-2, and F1-2: without ncAAs but containing IPTG) were used as controls to measure background signals. Substrate specificity profiles of PylRS-L3•tRNA$^{Pyl}_{CUA}$ pair were observed by *sfGFP-UAG2* gene suppression. The experiments were performed in the presence of 1 mM ncAAs and IPTG in GMML medium at 37℃ for 12 h. *E. coli* BL21 (DE3) was used in the experiment.

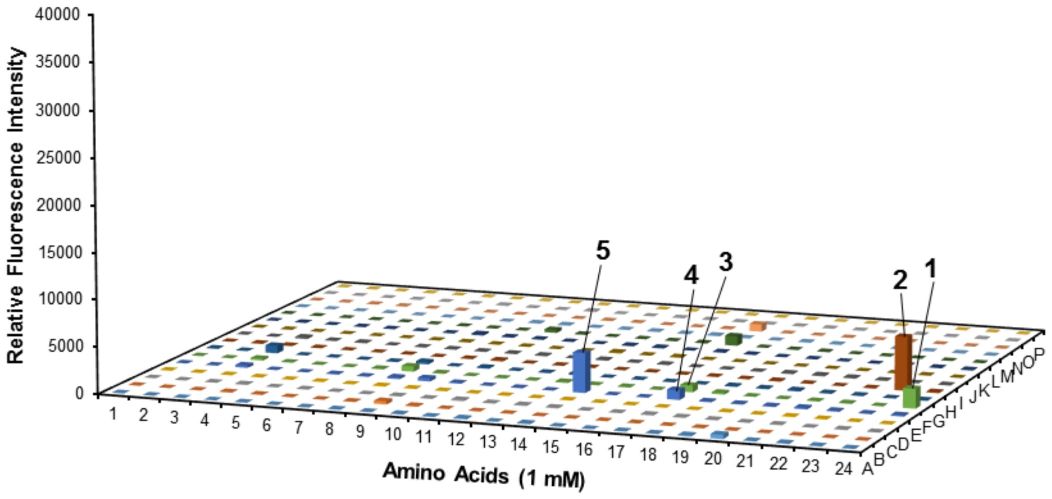

Figure S25. Substrate range of N-PylRS•tRNA$_{CUA}^{Pyl}$ pair in producing sfGFP-UAG27 proteins. *sfGFP-UAG27* gene read-through in ncAAs library was measured by fluorescence intensity. Twelve wells (A1-2, B1-2, and C1-2: without ncAAs and IPTG; D1-2, E1-2, and F1-2: without ncAAs but containing IPTG) were used as controls to measure background signals. Substrate specificity profiles of N-PylRS•tRNA$_{CUA}^{Pyl}$ pair were observed by *sfGFP-UAG27* gene suppression. The experiments were performed in the presence of 1 mM ncAAs and IPTG in GMML medium at 37℃ for 12 h. *E. coli* BL21 (DE3) was used in the experiment.

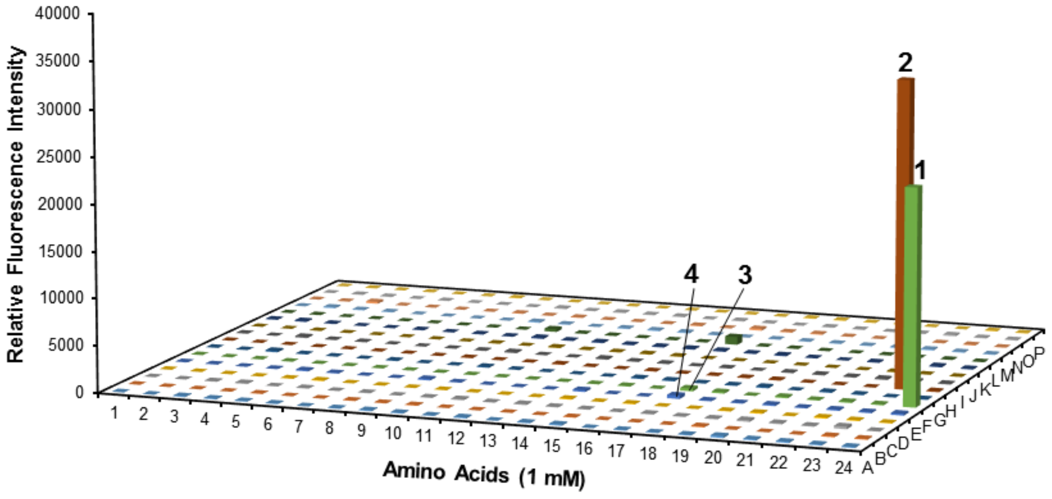

Figure S26. Substrate range of N-PylRS•tRNA$_{CUA}^{Pyl}$ pair in producing sfGFP-UAG2 proteins. *sfGFP-UAG2* gene read-through in ncAAs library was measured by fluorescence intensity. Twelve wells (A1-2, B1-2, and C1-2: without ncAAs and IPTG; D1-2, E1-2, and F1-2: without ncAAs but containing IPTG) were used as controls to measure background signals. Substrate specificity profiles of N-PylRS•tRNA$_{CUA}^{Pyl}$ pair were observed by *sfGFP-UAG2* gene suppression. The experiments were performed in the presence of 1 mM ncAAs and IPTG in GMML medium at 37℃ for 12 h. *E. coli* BL21 (DE3) was used in the experiment.

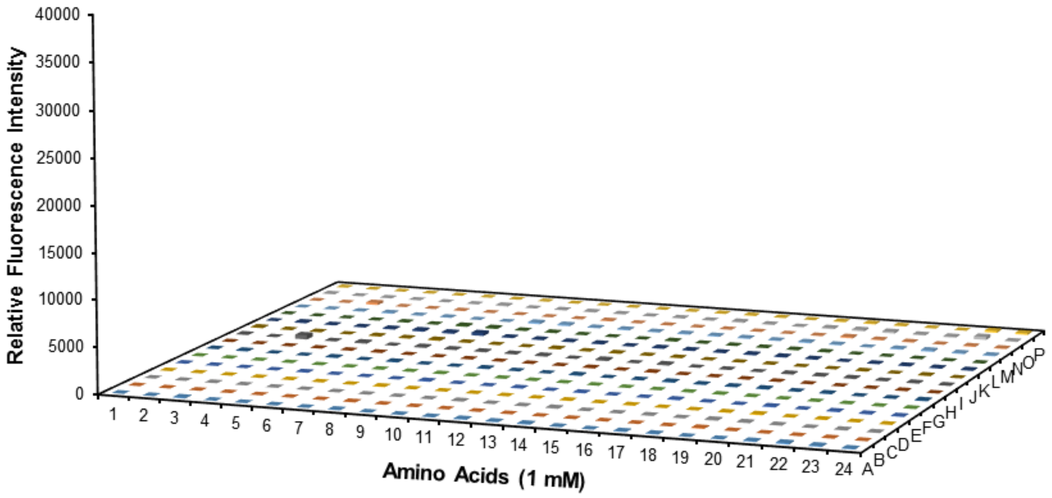

Figure S27. Substrate range of N-PylRS-D1•tRNA$^{Pyl}_{CUA}$ pair in producing sfGFP-UAG27 proteins. *sfGFP-UAG27* gene read-through in ncAAs library was measured by fluorescence intensity. Twelve wells (A1-2, B1-2, and C1-2: without ncAAs and IPTG; D1-2, E1-2, and F1-2: without ncAAs but containing IPTG) were used as controls to measure background signals. Substrate specificity profiles of N-PylRS-D1•tRNA$^{Pyl}_{CUA}$ pair were observed by *sfGFP-UAG27* gene suppression. The experiments were performed in the presence of 1 mM ncAAs and IPTG in GMML medium at 37℃ for 12 h. *E. coli* BL21 (DE3) was used in the experiment.

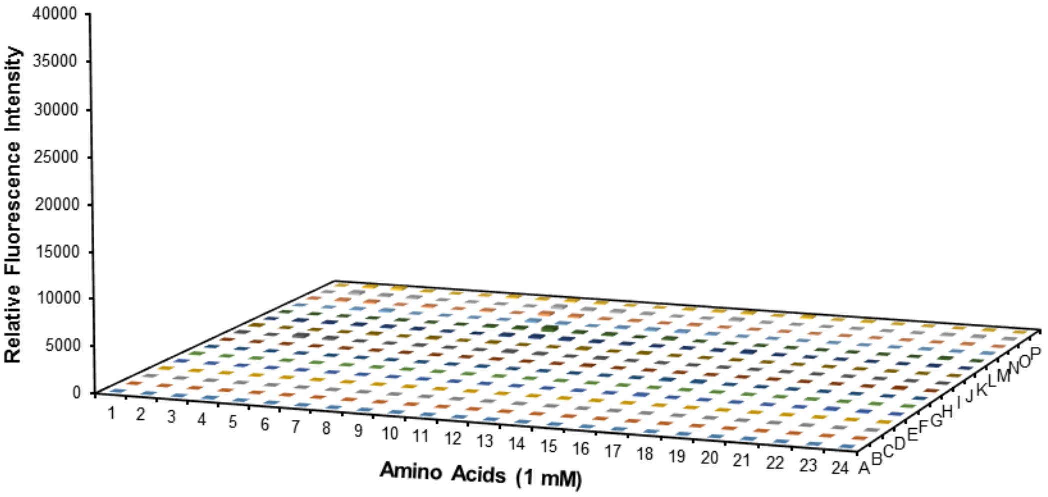

Figure S28. Substrate range of N-PylRS-D1•tRNA$^{Pyl}_{CUA}$ pair in producing sfGFP-UAG2 proteins. *sfGFP-UAG2* gene read-through in ncAAs library was measured by fluorescence intensity. Twelve wells (A1-2, B1-2, and C1-2: without ncAAs and IPTG; D1-2, E1-2, and F1-2: without ncAAs but containing IPTG) were used as controls to measure background signals. Substrate specificity profiles of N-PylRS-D1•tRNA$^{Pyl}_{CUA}$ pair were observed by *sfGFP-UAG2* gene suppression. The experiments were performed in the presence of 1 mM ncAAs and IPTG in GMML medium at 37℃ for 12 h. *E. coli* BL21 (DE3) was used in the experiment.

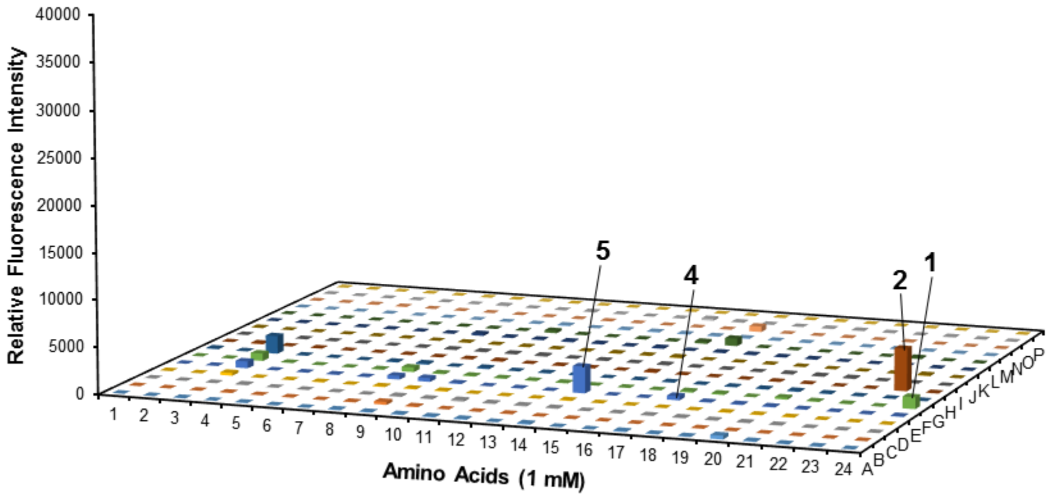

Figure S29. Substrate range of N-PylRS-L1•tRNA$^{Pyl}_{CUA}$ pair in producing sfGFP-UAG27 proteins. *sfGFP-UAG27* gene read-through in ncAAs library was measured by fluorescence intensity. Twelve wells (A1-2, B1-2, and C1-2: without ncAAs and IPTG; D1-2, E1-2, and F1-2: without ncAAs but containing IPTG) were used as controls to measure background signals. Substrate specificity profiles of N-PylRS-L1•tRNA$^{Pyl}_{CUA}$ pair were observed by *sfGFP-UAG27* gene suppression. The experiments were performed in the presence of 1 mM ncAAs and IPTG in GMML medium at 37℃ for 12 h. *E. coli* BL21 (DE3) was used in the experiment.

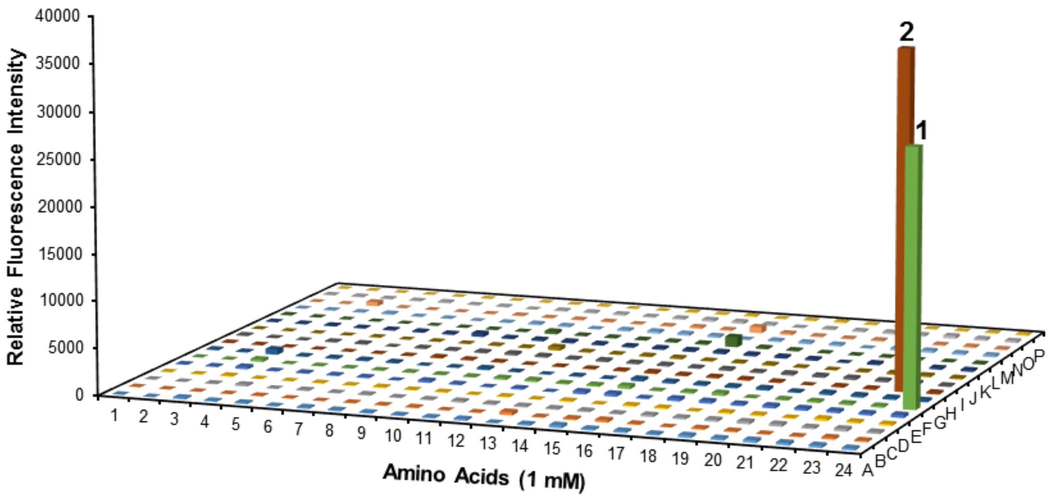

Figure S30. Substrate range of N-PylRS-L1•tRNA$^{Pyl}_{CUA}$ pair in producing sfGFP-UAG2 proteins. *sfGFP-UAG2* gene read-through in ncAAs library was measured by fluorescence intensity. Twelve wells (A1-2, B1-2, and C1-2: without ncAAs and IPTG; D1-2, E1-2, and F1-2: without ncAAs but containing IPTG) were used as controls to measure background signals. Substrate specificity profiles of N-PylRS-L1•tRNA$^{Pyl}_{CUA}$ pair were observed by *sfGFP-UAG2* gene suppression. The experiments were performed in the presence of 1 mM ncAAs and IPTG in GMML medium at 37℃ for 12 h. *E. coli* BL21 (DE3) was used in the experiment.

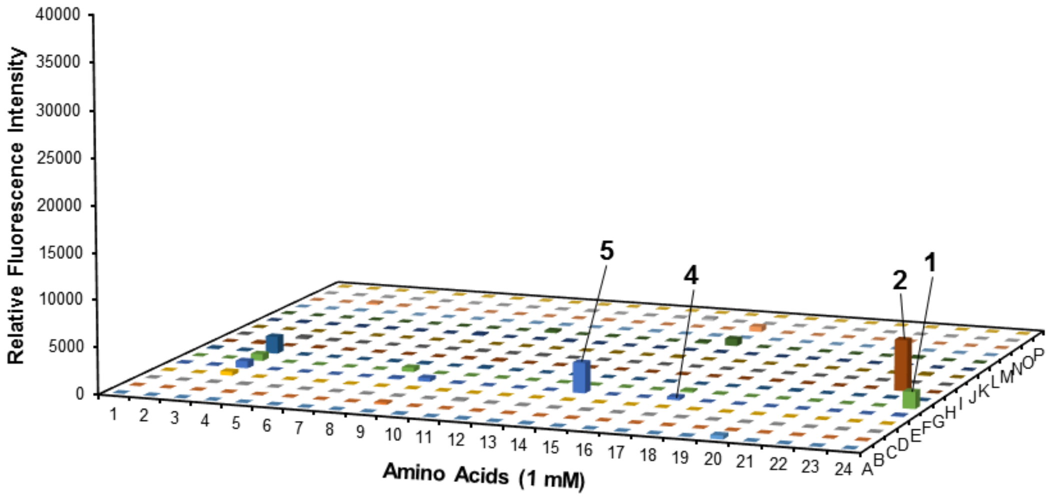

Figure S31. Substrate range of N-PylRS-L2•$tRNA^{Pyl}_{CUA}$ pair in producing sfGFP-UAG27 proteins. *sfGFP-UAG27* gene read-through in ncAAs library was measured by fluorescence intensity. Twelve wells (A1-2, B1-2, and C1-2: without ncAAs and IPTG; D1-2, E1-2, and F1-2: without ncAAs but containing IPTG) were used as controls to measure background signals. Substrate specificity profiles of N-PylRS-L2•$tRNA^{Pyl}_{CUA}$ pair were observed by *sfGFP-UAG27* gene suppression. The experiments were performed in the presence of 1 mM ncAAs and IPTG in GMML medium at 37℃ for 12 h. *E. coli* BL21 (DE3) was used in the experiment.

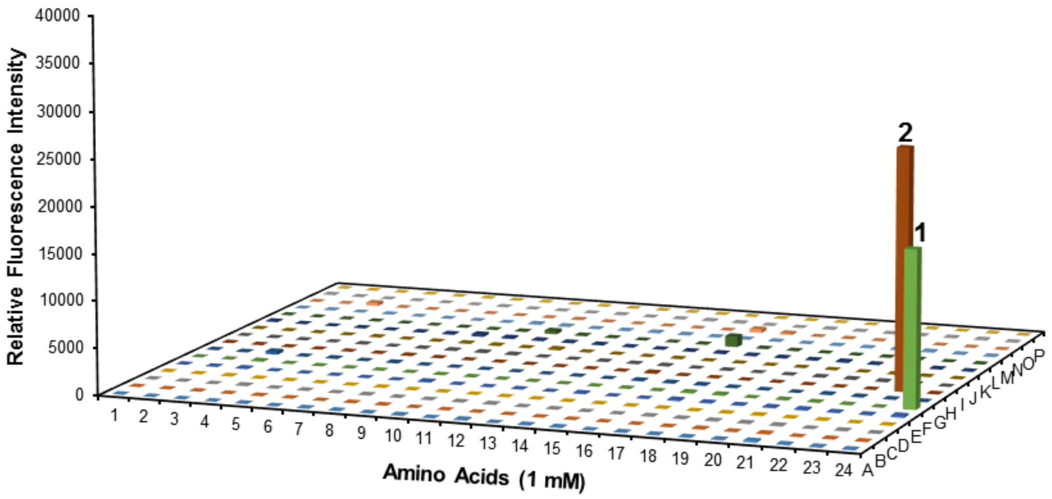

Figure S32. Substrate range of N-PylRS-L2•$tRNA^{Pyl}_{CUA}$ pair in producing sfGFP-UAG2 proteins. *sfGFP-UAG2* gene read-through in ncAAs library was measured by fluorescence intensity. Twelve wells (A1-2, B1-2, and C1-2: without ncAAs and IPTG; D1-2, E1-2, and F1-2: without ncAAs but containing IPTG) were used as controls to measure background signals. Substrate specificity profiles of N-PylRS-L2•$tRNA^{Pyl}_{CUA}$ pair were observed by *sfGFP-UAG2* gene suppression. The experiments were performed in the presence of 1 mM ncAAs and IPTG in GMML medium at 37℃ for 12 h. *E. coli* BL21 (DE3) was used in the experiment.

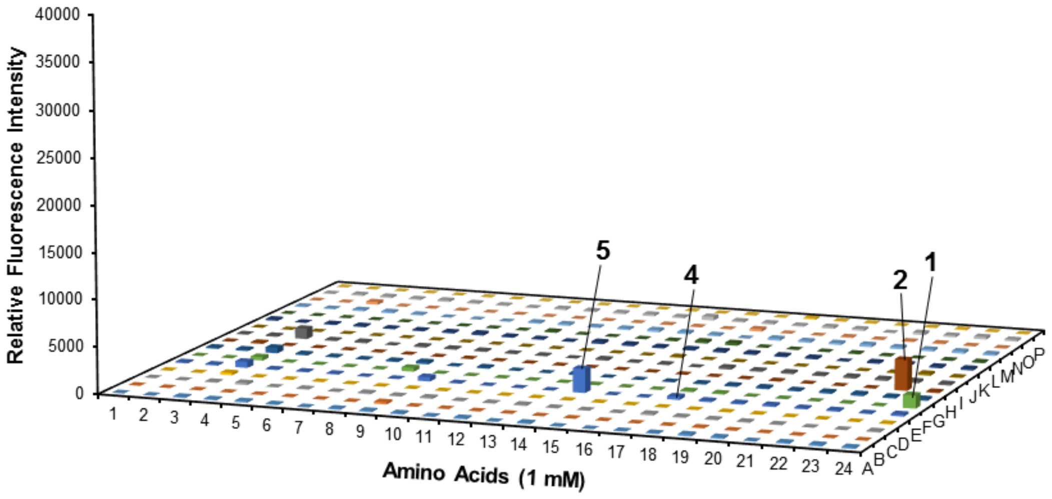

Figure S33. Substrate range of N-PylRS-L3•tRNA$^{Pyl}_{CUA}$ pair in producing sfGFP-UAG27 proteins. *sfGFP-UAG27* gene read-through in ncAAs library was measured by fluorescence intensity. Twelve wells (A1-2, B1-2, and C1-2: without ncAAs and IPTG; D1-2, E1-2, and F1-2: without ncAAs but containing IPTG) were used as controls to measure background signals. Substrate specificity profiles of N-PylRS-L3•tRNA$^{Pyl}_{CUA}$ pair were observed by *sfGFP-UAG27* gene suppression. The experiments were performed in the presence of 1 mM ncAAs and IPTG in GMML medium at 37℃ for 12 h. *E. coli* BL21 (DE3) was used in the experiment.

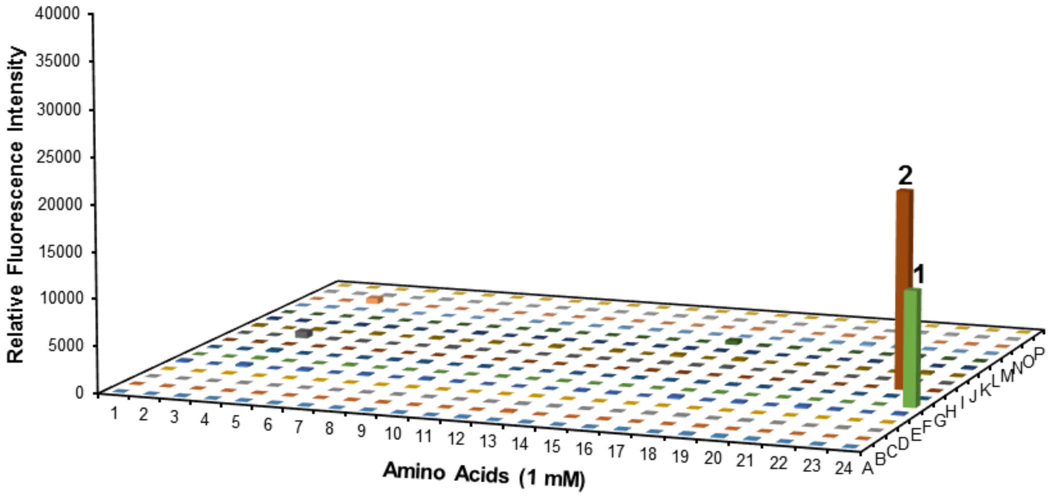

Figure S34. Substrate range of N-PylRS-L3•tRNA$^{Pyl}_{CUA}$ pair in producing sfGFP-UAG2 proteins. *sfGFP-UAG2* gene read-through in ncAAs library was measured by fluorescence intensity. Twelve wells (A1-2, B1-2, and C1-2: without ncAAs and IPTG; D1-2, E1-2, and F1-2: without ncAAs but containing IPTG) were used as controls to measure background signals. Substrate specificity profiles of N-PylRS-L3•tRNA$^{Pyl}_{CUA}$ pair were observed by *sfGFP-UAG2* gene suppression. The experiments were performed in the presence of 1 mM ncAAs and IPTG in GMML medium at 37℃ for 12 h. *E. coli* BL21 (DE3) was used in the experiment.

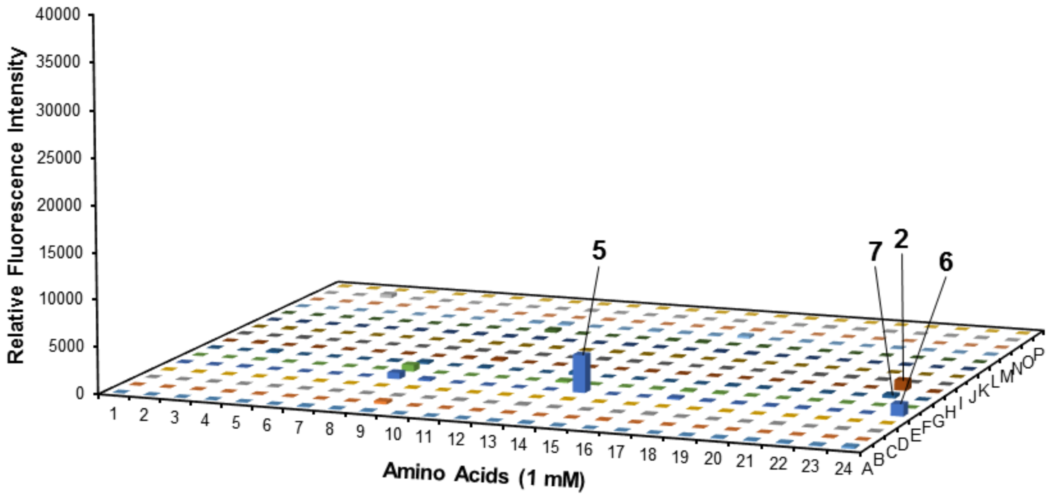

Figure S35. Substrate range of N-ZRS•tRNA$^{Pyl}_{CUA}$ pair in producing sfGFP-UAG27 proteins. *sfGFP-UAG27* gene read-through in ncAAs library was measured by fluorescence intensity. Twelve wells (A1-2, B1-2, and C1-2: without ncAAs and IPTG; D1-2, E1-2, and F1-2: without ncAAs but containing IPTG) were used as controls to measure background signals. Substrate specificity profiles of N-ZRS•tRNA$^{Pyl}_{CUA}$ pair were observed by *sfGFP-UAG27* gene suppression. The experiments were performed in the presence of 1 mM ncAAs and IPTG in GMML medium at 37℃ for 12 h. *E. coli* BL21 (DE3) was used in the experiment.

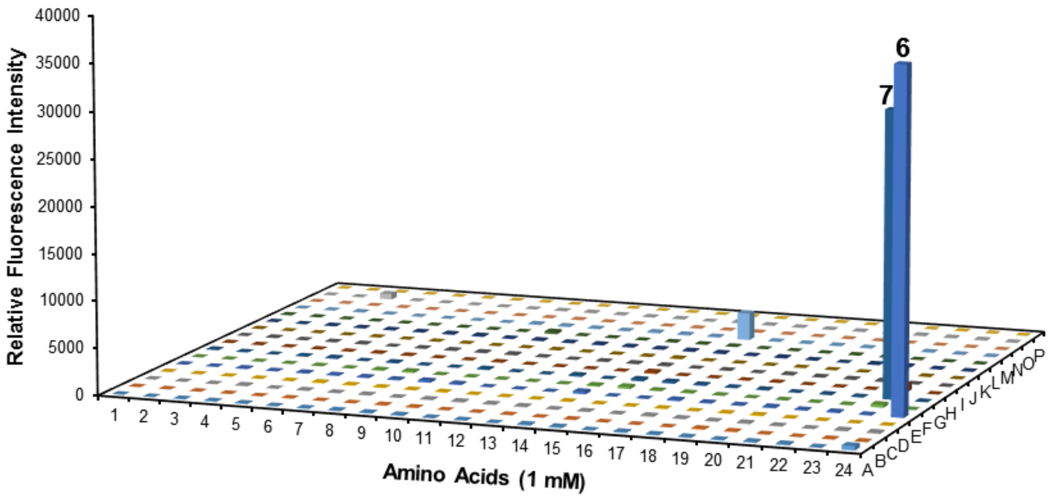

Figure S36. Substrate range of N-ZRS•tRNA$^{Pyl}_{CUA}$ pair in producing sfGFP-UAG2 proteins. *sfGFP-UAG2* gene read-through in ncAAs library was measured by fluorescence intensity. Twelve wells (A1-2, B1-2, and C1-2: without ncAAs and IPTG; D1-2, E1-2, and F1-2: without ncAAs but containing IPTG) were used as controls to measure background signals. Substrate specificity profiles of N-ZRS•tRNA$^{Pyl}_{CUA}$ pair were observed by *sfGFP-UAG2* gene suppression. The experiments were performed in the presence of 1 mM ncAAs and IPTG in GMML medium at 37℃ for 12 h. *E. coli* BL21 (DE3) was used in the experiment.

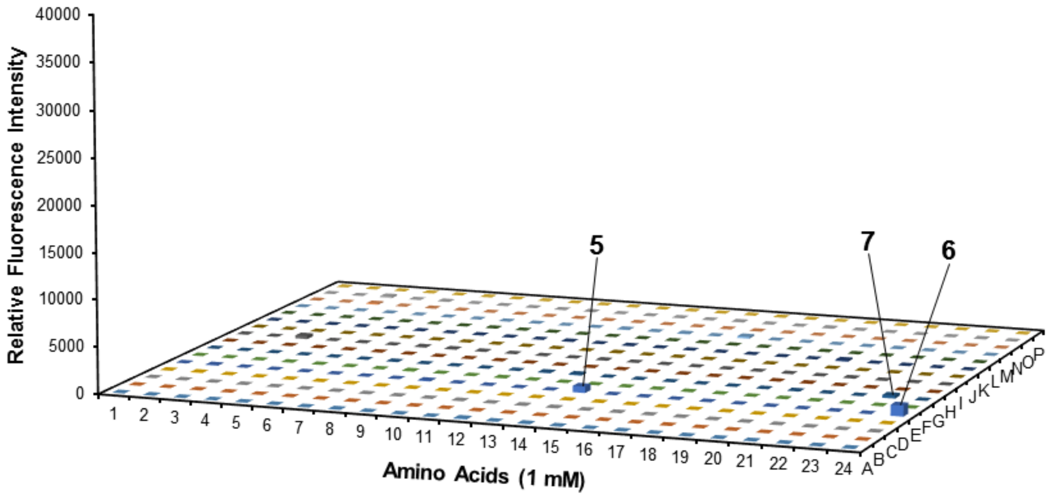

Figure S37. Substrate range of ZRS•tRNA$^{Pyl}_{CUA}$ pair in producing sfGFP-UAG27 proteins. *sfGFP-UAG27* gene read-through in ncAAs library was measured by fluorescence intensity. Twelve wells (A1-2, B1-2, and C1-2: without ncAAs and IPTG; D1-2, E1-2, and F1-2: without ncAAs but containing IPTG) were used as controls to measure background signals. Substrate specificity profiles of ZRS•tRNA$^{Pyl}_{CUA}$ pair were observed by *sfGFP-UAG27* gene suppression. The experiments were performed in the presence of 1 mM ncAAs and IPTG in GMML medium at 37℃ for 12 h. *E. coli* BL21 (DE3) was used in the experiment.

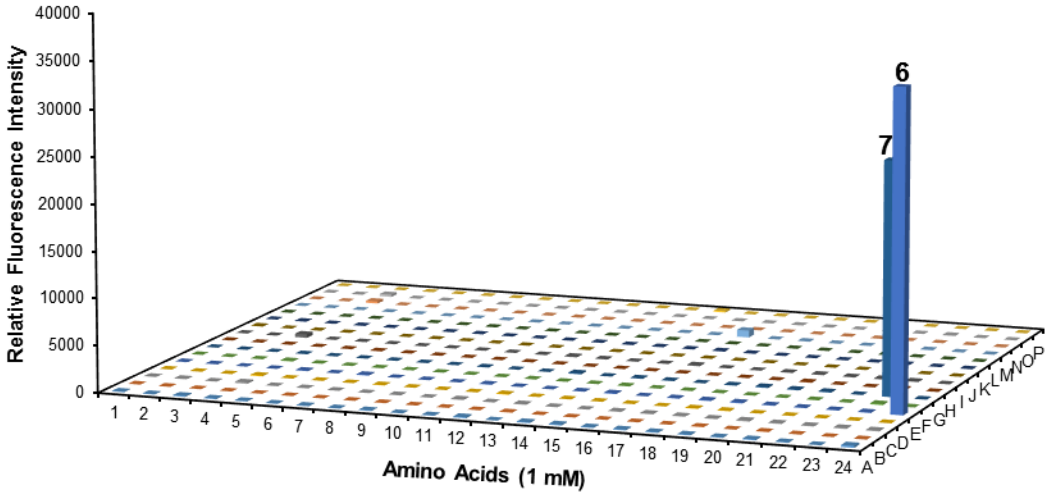

Figure S38. Substrate range of ZRS•tRNA$^{Pyl}_{CUA}$ pair in producing sfGFP-UAG2 proteins. *sfGFP-UAG2* gene read-through in ncAAs library was measured by fluorescence intensity. Twelve wells (A1-2, B1-2, and C1-2: without ncAAs and IPTG; D1-2, E1-2, and F1-2: without ncAAs but containing IPTG) were used as controls to measure background signals. Substrate specificity profiles of ZRS•tRNA$^{Pyl}_{CUA}$ pair were observed by *sfGFP-UAG2* gene suppression. The experiments were performed in the presence of 1 mM ncAAs and IPTG in GMML medium at 37℃ for 12 h. *E. coli* BL21 (DE3) was used in the experiment.

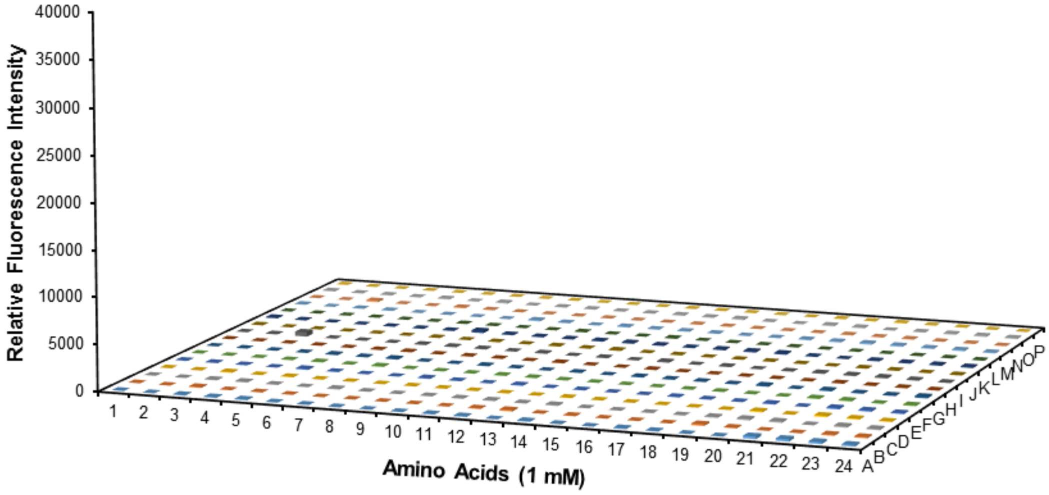

Figure S39. Substrate range of ZRS-D1•tRNA$^{Pyl}_{CUA}$ pair in producing sfGFP-UAG27 proteins. *sfGFP-UAG27* gene read-through in ncAAs library was measured by fluorescence intensity. Twelve wells (A1-2, B1-2, and C1-2: without ncAAs and IPTG; D1-2, E1-2, and F1-2: without ncAAs but containing IPTG) were used as controls to measure background signals. Substrate specificity profiles of ZRS-D1•tRNA$^{Pyl}_{CUA}$ pair were observed by *sfGFP-UAG27* gene suppression. The experiments were performed in the presence of 1 mM ncAAs and IPTG in GMML medium at 37°C for 12 h. *E. coli* BL21 (DE3) was used in the experiment.

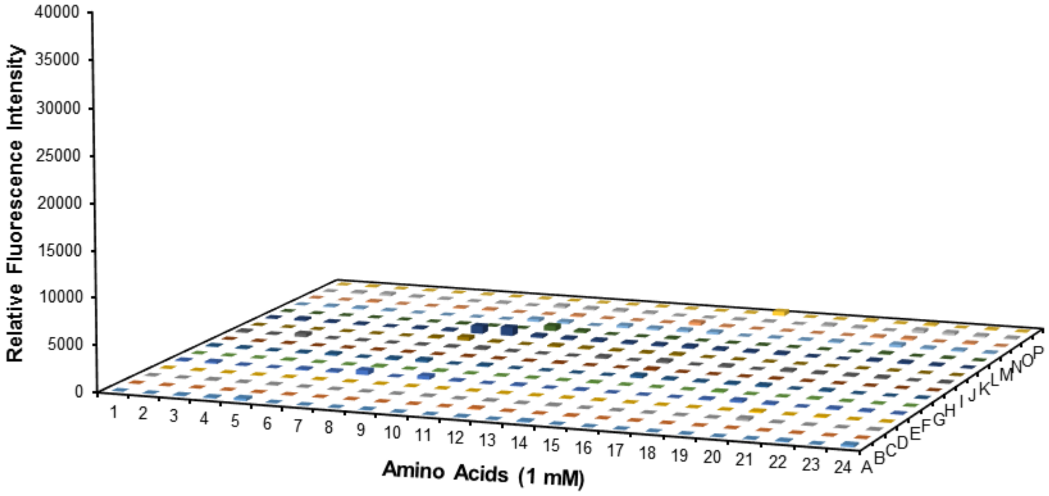

Figure S40. Substrate range of ZRS-D1•tRNA$^{Pyl}_{CUA}$ pair in producing sfGFP-UAG2 proteins. *sfGFP-UAG2* gene read-through in ncAAs library was measured by fluorescence intensity. Twelve wells (A1-2, B1-2, and C1-2: without ncAAs and IPTG; D1-2, E1-2, and F1-2: without ncAAs but containing IPTG) were used as controls to measure background signals. Substrate specificity profiles of ZRS-D1•tRNA$^{Pyl}_{CUA}$ pair were observed by *sfGFP-UAG2* gene suppression. The experiments were performed in the presence of 1 mM ncAAs and IPTG in GMML medium at 37°C for 12 h. *E. coli* BL21 (DE3) was used in the experiment.

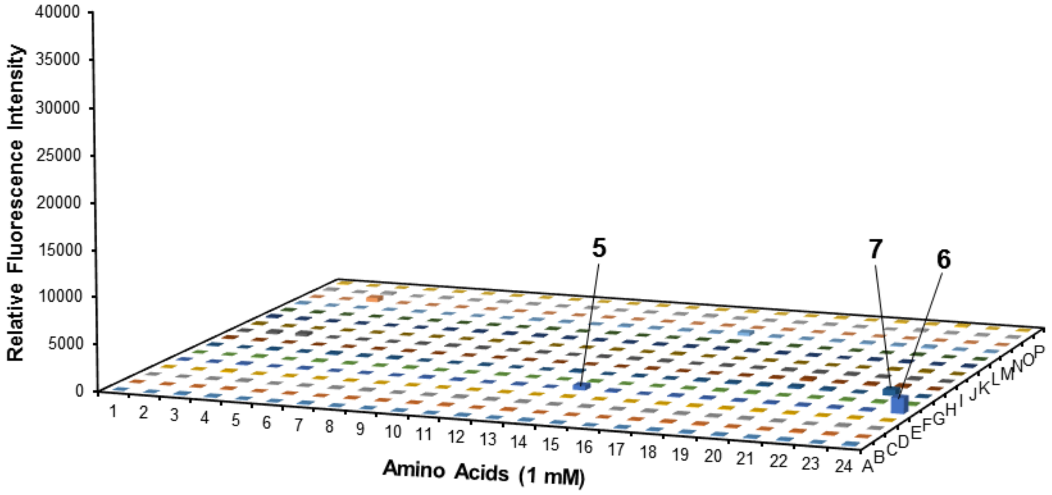

Figure S41. Substrate range of ZRS-L1•tRNA$^{Pyl}_{CUA}$ pair in producing sfGFP-UAG27 proteins. *sfGFP-UAG27* gene read-through in ncAAs library was measured by fluorescence intensity. Twelve wells (A1-2, B1-2, and C1-2: without ncAAs and IPTG; D1-2, E1-2, and F1-2: without ncAAs but containing IPTG) were used as controls to measure background signals. Substrate specificity profiles of ZRS-L1•tRNA$^{Pyl}_{CUA}$ pair were observed by *sfGFP-UAG27* gene suppression. The experiments were performed in the presence of 1 mM ncAAs and IPTG in GMML medium at 37℃ for 12 h. *E. coli* BL21 (DE3) was used in the experiment.

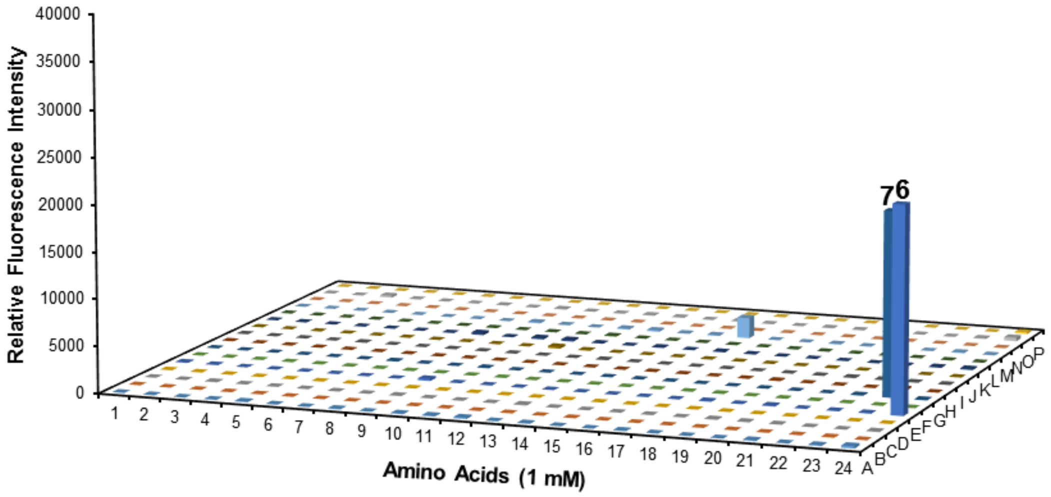

Figure S42. Substrate range of ZRS-L1•tRNA$^{Pyl}_{CUA}$ pair in producing sfGFP-UAG2 proteins. *sfGFP-UAG2* gene read-through in ncAAs library was measured by fluorescence intensity. Twelve wells (A1-2, B1-2, and C1-2: without ncAAs and IPTG; D1-2, E1-2, and F1-2: without ncAAs but containing IPTG) were used as controls to measure background signals. Substrate specificity profiles of ZRS-L1•tRNA$^{Pyl}_{CUA}$ pair were observed by *sfGFP-UAG2* gene suppression. The experiments were performed in the presence of 1 mM ncAAs and IPTG in GMML medium at 37℃ for 12 h. *E. coli* BL21 (DE3) was used in the experiment.

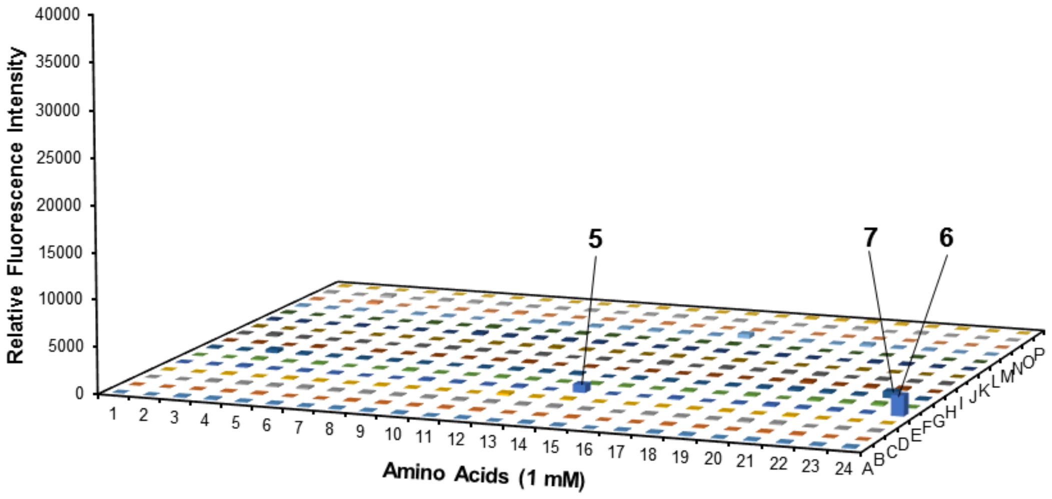

Figure S43. Substrate range of ZRS-L2•tRNA$^{Pyl}_{CUA}$ pair in producing sfGFP-UAG27 proteins. *sfGFP-UAG27* gene read-through in ncAAs library was measured by fluorescence intensity. Twelve wells (A1-2, B1-2, and C1-2: without ncAAs and IPTG; D1-2, E1-2, and F1-2: without ncAAs but containing IPTG) were used as controls to measure background signals. Substrate specificity profiles of ZRS-L2•tRNA$^{Pyl}_{CUA}$ pair were observed by *sfGFP-UAG27* gene suppression. The experiments were performed in the presence of 1 mM ncAAs and IPTG in GMML medium at 37℃ for 12 h. *E. coli* BL21 (DE3) was used in the experiment.

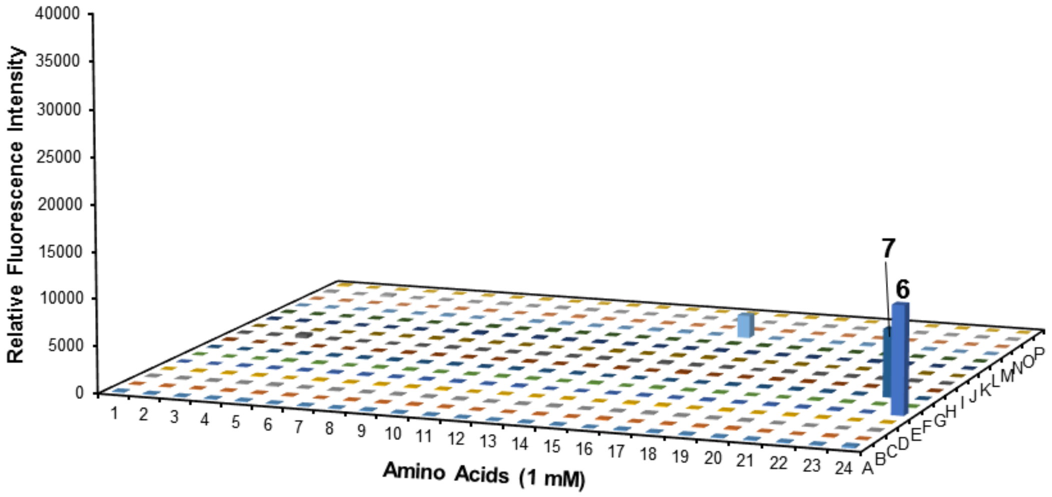

Figure S44. Substrate range of ZRS-L2•tRNA$^{Pyl}_{CUA}$ pair in producing sfGFP-UAG2 proteins. *sfGFP-UAG2* gene read-through in ncAAs library was measured by fluorescence intensity. Twelve wells (A1-2, B1-2, and C1-2: without ncAAs and IPTG; D1-2, E1-2, and F1-2: without ncAAs but containing IPTG) were used as controls to measure background signals. Substrate specificity profiles of ZRS-L2•tRNA$^{Pyl}_{CUA}$ pair were observed by *sfGFP-UAG2* gene suppression. The experiments were performed in the presence of 1 mM ncAAs and IPTG in GMML medium at 37℃ for 12 h. *E. coli* BL21 (DE3) was used in the experiment.

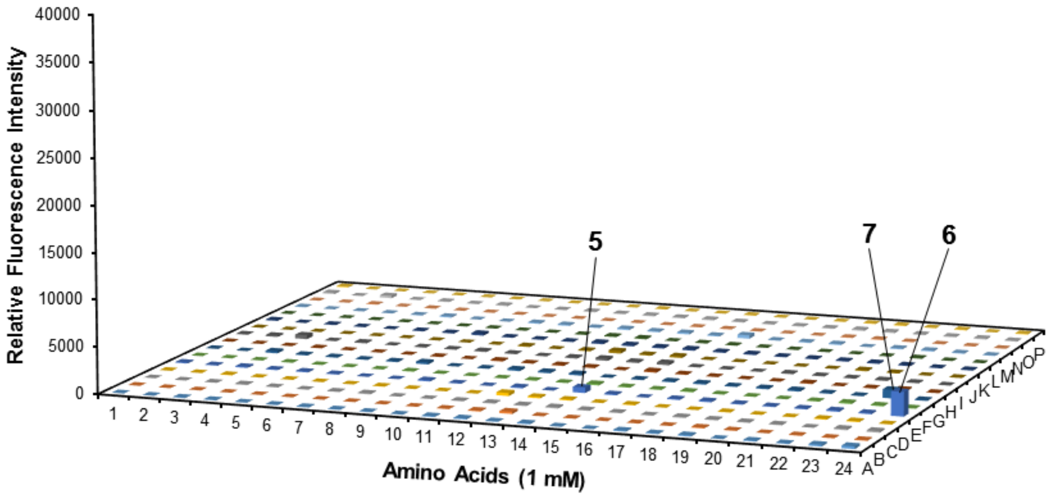

Figure S45. Substrate range of ZRS-L3•tRNA$^{Pyl}_{CUA}$ pair in producing sfGFP-UAG27 proteins. *sfGFP-UAG27* gene read-through in ncAAs library was measured by fluorescence intensity. Twelve wells (A1-2, B1-2, and C1-2: without ncAAs and IPTG; D1-2, E1-2, and F1-2: without ncAAs but containing IPTG) were used as controls to measure background signals. Substrate specificity profiles of ZRS-L3•tRNA$^{Pyl}_{CUA}$ pair were observed by *sfGFP-UAG27* gene suppression. The experiments were performed in the presence of 1 mM ncAAs and IPTG in GMML medium at 37℃ for 12 h. *E. coli* BL21 (DE3) was used in the experiment.

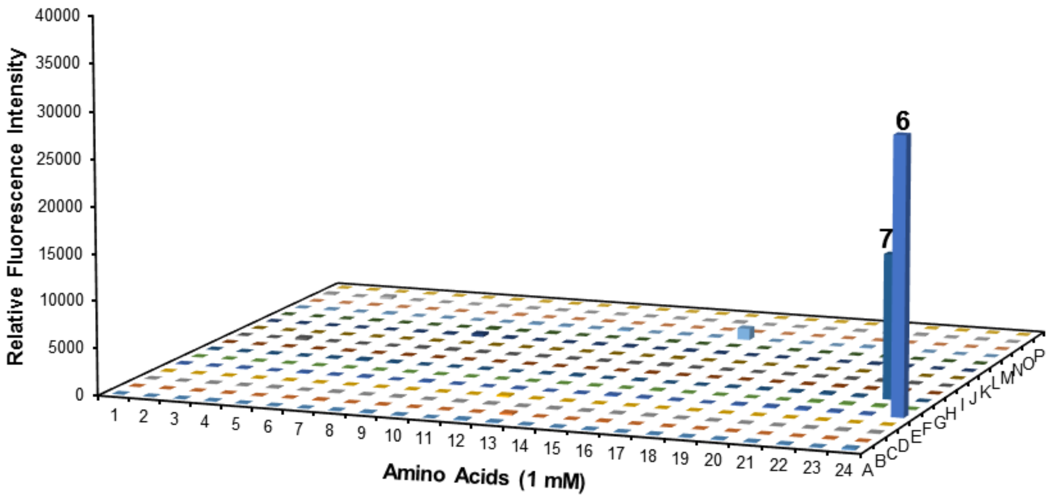

Figure S46. Substrate range of ZRS-L3•tRNA$^{Pyl}_{CUA}$ pair in producing sfGFP-UAG2 proteins. *sfGFP-UAG2* gene read-through in ncAAs library was measured by fluorescence intensity. Twelve wells (A1-2, B1-2, and C1-2: without ncAAs and IPTG; D1-2, E1-2, and F1-2: without ncAAs but containing IPTG) were used as controls to measure background signals. Substrate specificity profiles of ZRS-L3•tRNA$^{Pyl}_{CUA}$ pair were observed by *sfGFP-UAG2* gene suppression. The experiments were performed in the presence of 1 mM ncAAs and IPTG in GMML medium at 37℃ for 12 h. *E. coli* BL21 (DE3) was used in the experiment.

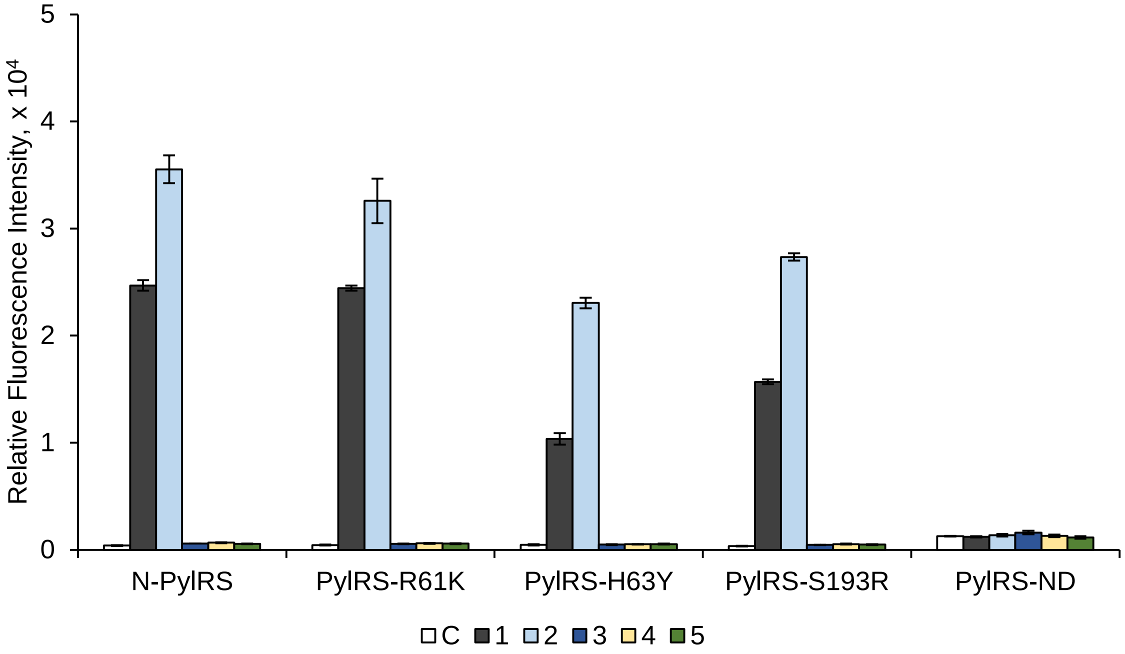

Figure S47. The *sfGFP-UAG2* gene suppression efficiencies of PylRS enzyme variants. Incorporation efficiencies of PylRS variants as measured by fluorescence intensities of sfGFP with an amber mutation at position 2. Proteins were expressed in 1 mM ncAA and IPTG in GMML medium at 37℃ for 12 h. Cells were excited at 485 nm and the fluorescence intensities were detected at 535 nm. The cell density was monitored by absorbance at 595 nm. C indicates the control experiments that cells with the supplement of 1 mM IPTG; 1–5 denote the supplement of 1 mM IPTG and ncAA **1**–**5** (Scheme 1). Background signals from cells without adding IPTG were subtracted from each group. Error bars represent the standard deviation of sfGFP production from four repeated experiments.

4. Table S1. Chemical Names and Structures in ncAAs Library

Position	CAS No.	Name	Chemical Structure
A01	N/A	N/A	N/A
A02	N/A	N/A	N/A
A03	42538-40-9	2-Bromo-L-phenylalanine	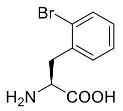
A04	57213-48-6	3-Cyano-L-phenylalanine	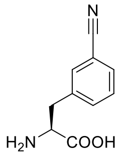
A05	126109-42-0	4-Carboxy-L-phenylalanine	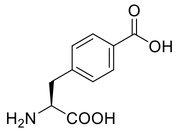
A06	24250-85-9	4-Iodo-L-phenylalanine	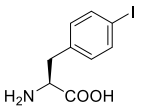
A07	111119-36-9	L-2,4-Dichlorophenylalanine	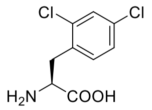
A08	2566-30-5	N^{α}-Methyl-L-phenylalanine hydrochloride	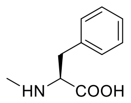
A09	37535-49-2	3-(4'-Pyridyl)-L-alanine	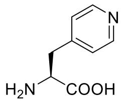
A10	31105-93-8	2,4-Difluoro-L-phenylalanine	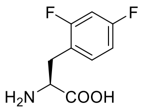
A11	515-30-0	α-Phenyllactic acid	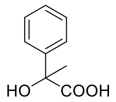
A12	6636-22-2	*O*-Acetyl-L-tyrosine	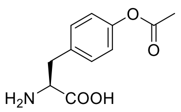
A13	1596-67-4	L-Thyronine	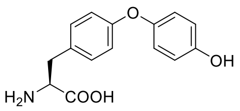
A14	32381-18-3	DL-α-Methylhistidine dihydrochloride	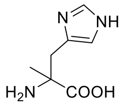

A15	154-08-5	5-Fluoro-DL-tryptophan	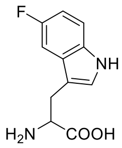
A16	1954-45-6	4-Methyl-DL-tryptophan	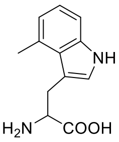
A17	638-23-3	*S*-Carboxymethyl-L-cysteine	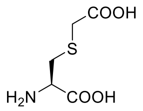
A18	387868-34-0	*S*-4-Methoxybenzyl-L-penicillamine	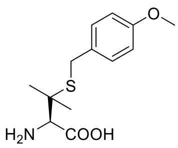
A19	16338-48-0	H-Allyl-L-glycine	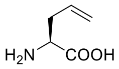
A20	6600-40-4	L-Norvaline	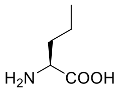
A21	62-57-7	α-Aminoisobutyric acid	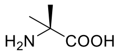
A22	25528-71-6	b-Cyclohexyl-L-alanine hydrochloride	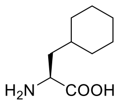
A23	66638-22-0	*O*-Acetyl-L-serine hydrochloride	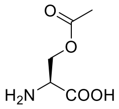
A24	692-04-6	N^{ε}-Acetyl-L-lysine	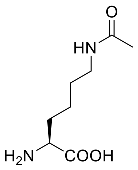
B01	N/A	N/A	N/A
B02	N/A	N/A	N/A
B03	167817-55-2	2-Iodo-L-phenylalanine	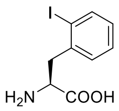
B04	19883-74-0	3-Nitro-L-phenylalanine	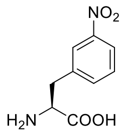
B05	223593-04-2	L-4-Carbamoylphenylalanine	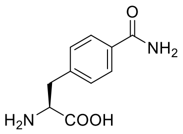

B06	155760-02-4	H-*p*-Phenyl-L-Phenylalanine	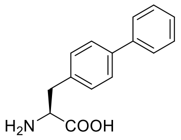
B07	49607-21-8	L-2,4-Dinitrophenylalanine	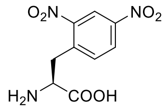
B08	62777-25-7	L-2-Amino-5-phenylpentanoic acid	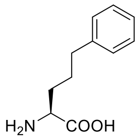
B09	64090-98-8	3-(3'-Pyridyl)-L-alanine dihydrochloride	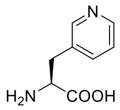
B10	31105-91-6	L-3,5-Difluorophenylalanine	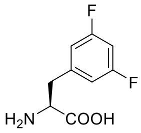
B11	149597-92-2	3,3-Diphenyl-L-alanine	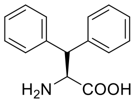
B12	18835-59-1	3,5-Diiodo-L-tyrosine dihydrate	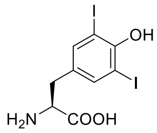
B13	55-06-1	3,3',5-Triiodo-L-thyronine sodium salt	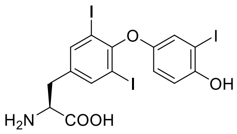
B14	2002-22-4	2-Mercapto-L-histidine	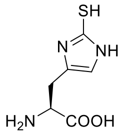
B15	55516-54-6	3-(1-Naphthyl)-L-alanine	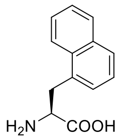
B16	33599-61-0	6-Bromo-DL-tryptophan	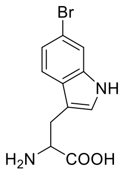
B17	28798-28-9	*S*-Acetamidomethyl-L-cysteine hydrochloride	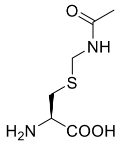

B18	1039102-11-8	*S*-4-Methylbenzyl-L-penicillamine	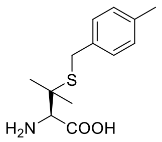
B19	144-24-1	DL-α-Methylleucine	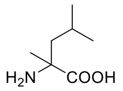
B20	6232-19-5	β-Cyano-L-alanine	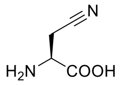
B21	54897-59-5	DL-2,3-Diaminopropionic acid monohydrochloride	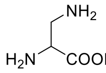
B22	23235-01-0	L-Propargylglycine	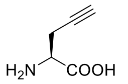
B23	18822-58-7	*O*-*tert*-Butyl-L-serine	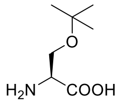
B24	2259-86-1	N^{ε}-Dimethyl-L-lysine hydrochloride	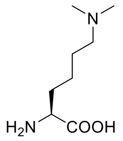
C01	N/A	N/A	N/A
C02	N/A	N/A	N/A
C03	263396-42-5	2-Cyano-L-phenylalanine	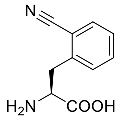
C04	19883-75-1	2-Nitro-L-phenylalanine	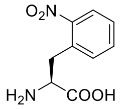
C05	122555-04-8	L-4-Acetylphenylalanine	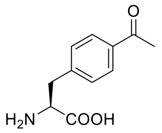
C06	949-99-5	4-Nitro-L-phenylalanine	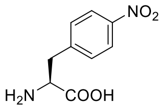
C07	52794-99-7	L-3,4-Dichlorophenylalanine	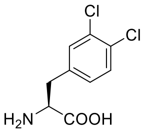
C08	267650-37-3	3-Styryl-L-alanine	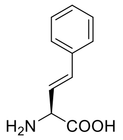

C09	37535-51-6	3-(2'-Pyridyl)-L-alanine	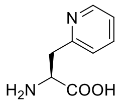
C10	31105-90-5	L-3,4-Difluorophenylalanine	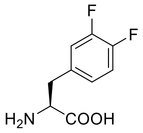
C11	57213-47-5	L-3-Aminomethylphenylalanine	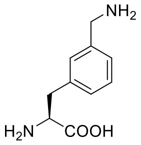
C12	6230-11-1	*O*-Methyl-L-tyrosine	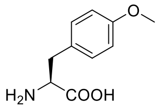
C13	21820-51-9	*O*-Phospho-L-tyrosine	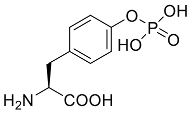
C14	332-80-9	1-Methyl-L-Histidine	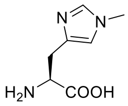
C15	72120-71-9	3-Benzothienyl-L-alanine	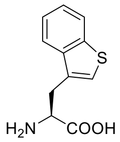
C16	852391-45-8	7-Bromo-DL-tryptophan	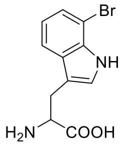
C17	2481-09-6	*S*-*tert*-Butyl-L-cysteine hydrochloride	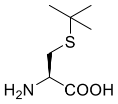
C18	4099-35-8	*S*-(2)-Aminoethyl-L-cysteine hydrochloride	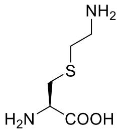
C19	155239-51-3	L-β-indanylglycine	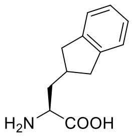
C20	57224-50-7	β-*tert*-Butyl-L-alanine	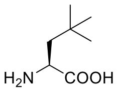
C21	594-61-6	α-Hydroxyisobutyric acid	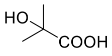

C22	2521-84-8	L-Cyclopentylglycine	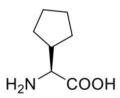
C23	32595-59-8	DL-β-(2-Thienyl)-serine	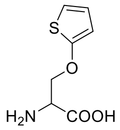
C24	55528-53-5	N^{ε}-(trimethyl)-L-lysine chloride	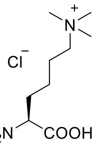
D01	N/A	N/A	N/A
D02	N/A	N/A	N/A
D03	14464-68-7	3-(Trifluoromethyl)-L-phenylalanine	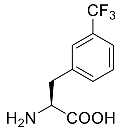
D04	1217651-22-3	L-3-Carbamoylphenylalanine	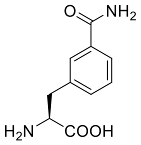
D05	82372-74-5	4-*tert*-Butyl-L-phenylalanine	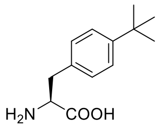
D06	943-80-6	4-Amino-L-phenylalanine	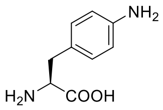
D07	32161-30-1	L-3,4-Dimethoxyphenylalanine	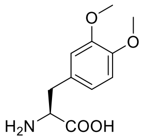
D08	70663-55-7	*N*-Methyl-4-nitro-L-phenylalanine	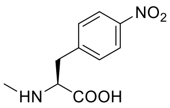
D09	270262-99-2	(3-Thienyl)-L-β-homoalanine	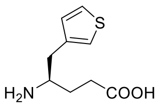
D10	749847-57-2	L-2,4,5-Trifluorophenylalanine	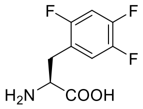
D11	621-44-3	3-Nitro-L-tyrosine	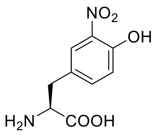

D12	51-48-9	L-Thyroxine	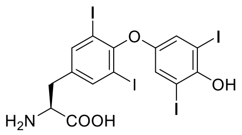
D13	775-06-4	DL-*m*-Tyrosine	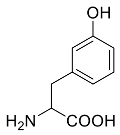
D14	2022956-35-8	N^{im}-Benzyl-D-histidine	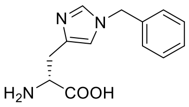
D15	6383-69-3	5-Amino-DL-tryptophan	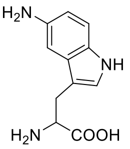
D16	153-91-3	DL-α-Methyl-tryptophan	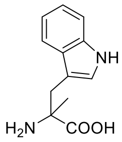
D17	30044-51-0	*S*-tert-Butylthio-L-cysteine	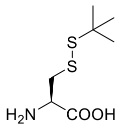
D18	7314-32-1	L-Methionine sulfone	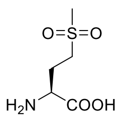
D19	13748-90-8	L-α-Hydroxyisocaproic acid	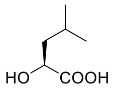
D20	27527-05-5	β-Cyclohexyl-L-alanine	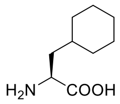
D21	1492-24-6	L-α-Aminobutyric acid	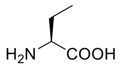
D22	626-69-7	*O*-Sulfo-L-serine	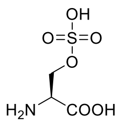
D23	10009-20-8	N^{ε}-Trifluoroacetyl-L-lysine	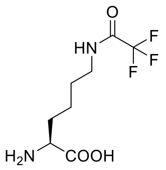

D24	7622-29-9	N^{ε}-Methyl-L-lysine hydrochloride	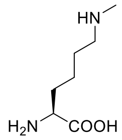
E01	N/A	N/A	N/A
E02	N/A	N/A	N/A
E03	80126-51-8	3-Chloro-L-phenylalanine	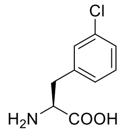
E04	58438-03-2	3-(2-Naphthyl)-L-alanine	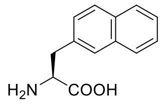
E05	1991-87-3	4-Methyl-L-phenylalanine	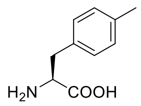
E06	104531-20-6	4-Cyano-L-phenylalanine	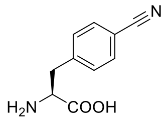
E07	2935-35-5	L-Phenylglycine	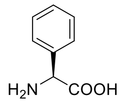
E08	3685-51-6	β-(3-Thienyl)-L-alanine	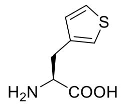
E09	19883-78-4	2-Fluoro-L-phenylalanine	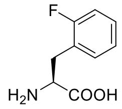
E10	646066-73-1	3,4,5-Trifluoro-L-phenylalanine	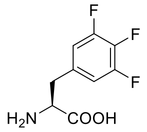
E11	23279-22-3	3-Amino-L-tyrosine	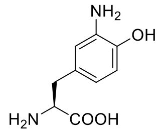
E12	7423-93-0	3-Chloro-L-tyrosine	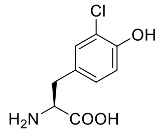
E13	17360-11-1	3,5-Dinitro-L-tyrosine monohydrate	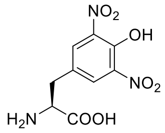

E14	368-16-1	H-*N*-3-Methyl-L-histidine	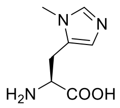
E15	25631-05-4	4-Fluoro-DL-tryptophan	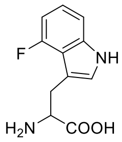
E16	25197-99-3	5-Bromo-L-tryptophan	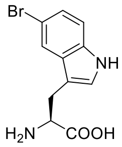
E17	2544-31-2	*S*-4-Methoxybenzyl-L-cysteine	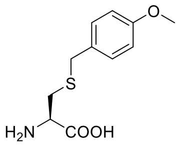
E18	87392-13-0	4,5-Dehydro-L-leucine	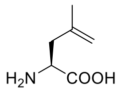
E19	2566-29-2	Diethylglycine	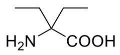
E20	99295-82-6	β-Cyclopentyl-L-alanine	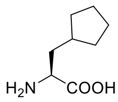
E21	7685-44-1	H-Allyl-DL-glycine	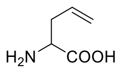
E22	4726-96-9	*O*-Benzyl-L-serine	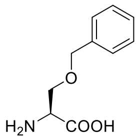
E23	10009-97-9	N^{δ}-Phthaloyl-L-ornithine hydrochloride	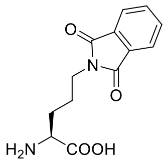
E24	1155-64-2	N^{ε}-Z-L-lysine	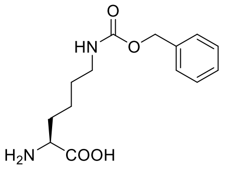
F01	N/A	N/A	N/A
F02	N/A	N/A	N/A
F03	82311-69-1	3-Bromo-L-phenylalanine	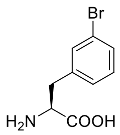

F04	150338-20-8	4-(Aminomethyl)-L-phenylalanine	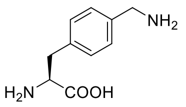
F05	114926-38-4	*p*-Trifluoromethyl-L-phenylalanine	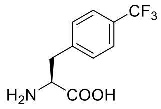
F06	1080496-42-9	4-Propargyloxy-L-phenylalanine	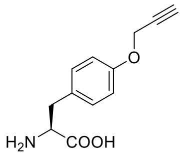
F07	943-73-7	L-Homophenylalanine	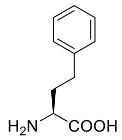
F08	22951-96-8	β-(2-Thienyl)-L-alanine	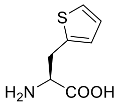
F09	19883-77-3	3-Fluoro-L-phenylalanine	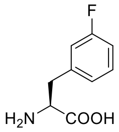
F10	138109-65-6	Pentafluoro-L-phenylalanine	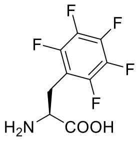
F11	21106-04-7	*O*-*Z*-L-tyrosine	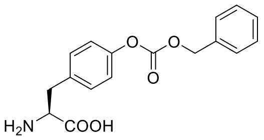
F12	300-38-9	L-3,5-Dibromotyrosine	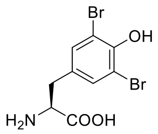
F13	119433-80-6	3-(4-Thiazolyl)-L-alanine	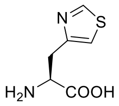
F14	161513-46-8	3-(2'-Quinolyl)-L-alanine	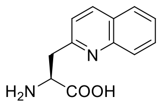
F15	7730-20-3	6-Fluoro-DL-tryptophan	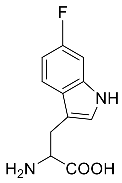

F16	17332-70-6	7-Methyl-DL-tryptophan	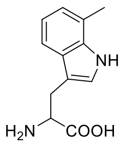
F17	42294-52-0	*S*-4-Methylbenzyl-L-cysteine	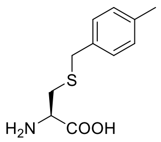
F18	96386-92-4	L-Homoleucine Hydrochloride	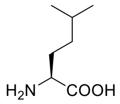
F19	1883-09-6	L-2,4-Diaminobutyric acid dihydrochloride	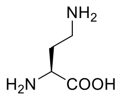
F20	102735-53-5	H-β-Cyclopropyl-L-Alanine	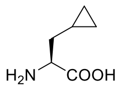
F21	14328-51-9	L-2-Cyclohexylglycine	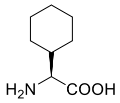
F22	32595-59-8	DL-β-(2-Thienyl)-serine	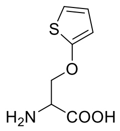
F23	3557-90-2	N^{ε}-4-Nitro-Z-L-lysine	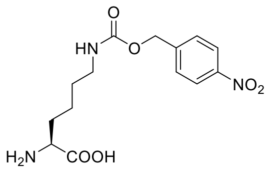
F24	2418-95-3	N^{ε}-Boc-L-Lysine	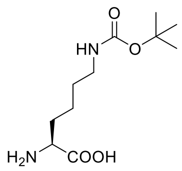
G01	80126-53-0	2-Methyl-L-phenylalanine	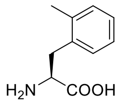
G02	193546-31-5	L-2-Methoxyphenylalanine	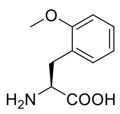
G03	20846-39-3	3-Iodo-L-phenylalanine	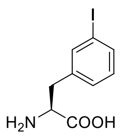

G04	74578-48-6	4-(Boc-amino)-L-phenylalanine	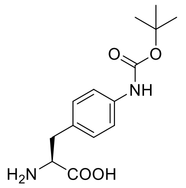
G05	14173-39-8	4-Chloro-L-phenylalanine	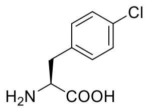
G06	104504-45-2	4-Benzoyl-L-phenylalanine	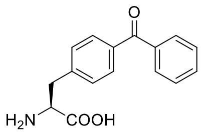
G07	23239-35-2	α-Methyl-L-phenylalanine	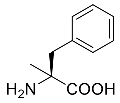
G08	127682-08-0	H-β-(2-Furyl)-L-alanine	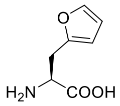
G09	1132-68-9	4-Fluoro-L-phenylalanine	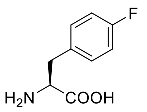
G10	889957-22-6	(*R,S*)2-Hydroxy-3-(3-pyridyl)propionic acid	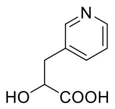
G11	16652-64-5	*O*-Benzyl-L-tyrosine	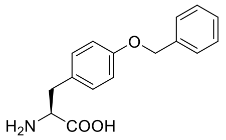
G12	40298-69-9	*O*-2,6-Dichlorobenzyl-L-tyrosine	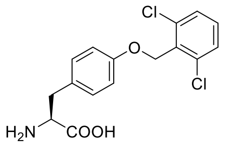
G13	1596-65-2	β-(2-Thiazolyl)-DL-alanine	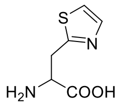
G14	4350-09-8	5-Hydroxy-L-tryptophan	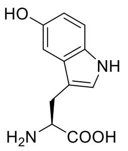
G15	146645-63-8	N^{in}-Boc-L-tryptophan	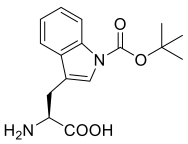

G16	1821-52-9	DL-Indole-3-lactic acid	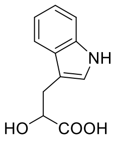
G17	3054-01-1	*S*-Benzyl-L-cysteine	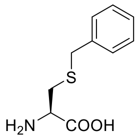
G18	20859-02-3	L-α-*tert*-Butyl-Gly-OH	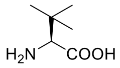
G19	1509-34-8	L-allo-Isoleucine	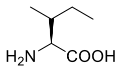
G20	1492-24-6	L-α-Aminobutyric acid	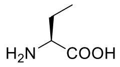
G21	33105-81-6	DL-α-tert-Butylglycine	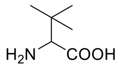
G22	407-41-0	*O*-Phospho-L-serine	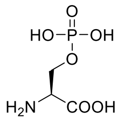
G23	42390-97-6	N^{ε}-2-Chloro-Z-L-lysine	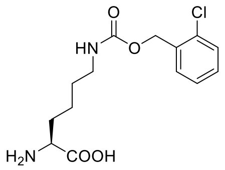
G24	3184-13-2	L-Ornithine hydrochloride	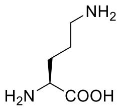
H01	119009-47-1	H-L-Phe(2-trifluoromethyl)-OH	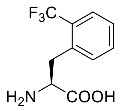
H02	103616-89-3	2-Chloro-L-phenylalanine	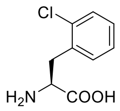
H03	57213-47-5	L-3-Aminomethylphenylalanine	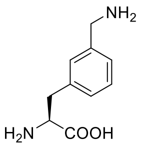
H04	33173-53-4	4-Azido-L-phenylalanine	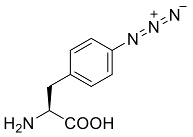

H05	24250-84-8	4-Bromo-L-phenylalanine	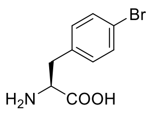
H06	259726-56-2	L-2,4-Dimethylphenylalanine	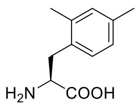
H07	130855-57-1	α-Methyl-L-4-Fluorophenylalanine	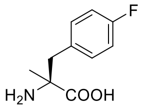
H08	154593-58-5	L-2-(5-Bromothienyl)alanine	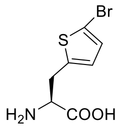
H09	33787-05-2	2,6-Difluoro-L-phenylalanine	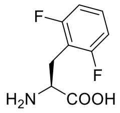
H10	20312-36-1	L-β-Phenyllactic acid	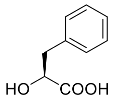
H11	18822-59-8	*O*-*tert*-Butyl-L-tyrosine	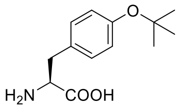
H12	52939-33-0	*N*-Methyl-*O*-methyl-L-tyrosine hydrochloride	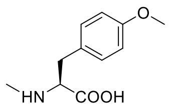
H13	16832-24-9	N^{im}-Benzyl-L-histidine	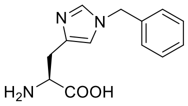
H14	25197-96-0	5-Methoxy-L-tryptophan	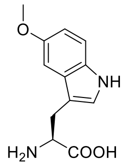
H15	38023-86-8	N^{in}-Formyl-L-tryptophan hydrochloride	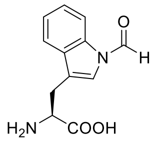
H16	5191-80-0	*S*-Diphenylmethyl-L-cysteine	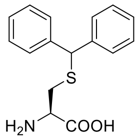
H17	1113-41-3	L-Penicillamine	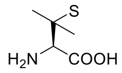

H18	2566-31-6	Di-n-propylglycine	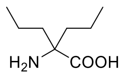
H19	17407-55-5	L-α-Hydroxyisovaleric acid	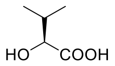
H20	2731-73-9	β-Chloro-L-alanine	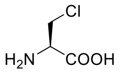
H21	208660-68-8	H-β-(7-Methoxycoumarin-4-yl)-Ala-OH	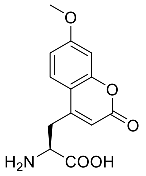
H22	672-15-1	L-Homoserine	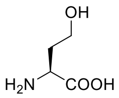
H23	6298-03-9	N^{ε}-Allyloxycarbonyl-L-lysine	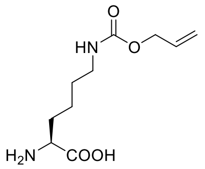
H24	156463-09-1	N^{δ}-Azido-L-Ornithine hydrochloride	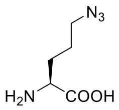
I01	1190-49-4	L-Homocitrulline	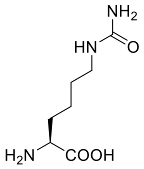
I02	200116-81-0	N^{ω}-(2,2,4,6,7-Pentamethyldihydrobenzofuran)-5-sulfonyl-D-arginine	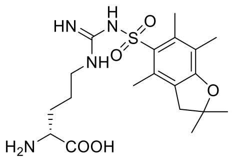
I03	2922-83-0	L-Kynurenine	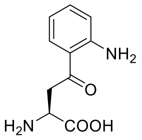
I04	112471-82-6	L-Glutamic acid-γ-cyclohexyl ester	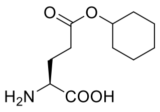
I05	61884-74-0	L-β-Homoglutamic acid hydrochloride	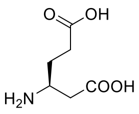

I06	2438-57-5	*cis* -4-Fluoro-L-proline	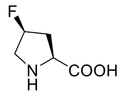
I07	1279049-67-0	(*R*)-γ-Benzyl-L-proline·HCl	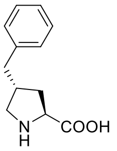
I08	103733-65-9	(3*R*)-1,2,3,4-Tetrahydroisoquinoline-3-carboxylic acid	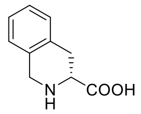
I09	54631-81-1	Boc-*O* -benzyl-L-*trans* -4-hydroxyproline	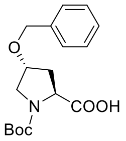
I10	56-86-0	L-Glutamic acid	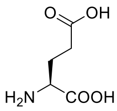
I11	72-19-5	L-Threonine	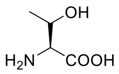
I12	5959-95-5	D-Glutamine	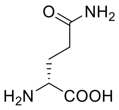
I13	157-06-2	D-Arginine	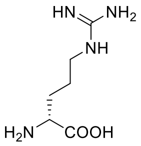
I14	153-94-6	D-tryptophan	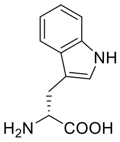
I15	169530-97-6	3-Nitro-D-phenylalanine	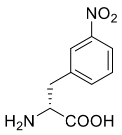
I16	2578-33-8	D-Glutamic acid-γ-benzyl ester	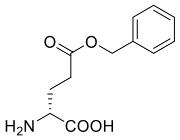
I17	264903-53-9	3-Styryl-D-alanine	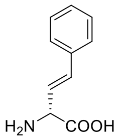

I18	270063-44-0	L-β-HomoAla(3-benzothienyl)-OH·HCl	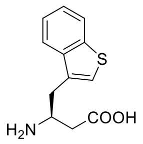
I19	270062-92-5	3-Methyl-L-β-homophenylalanine hydrochloride	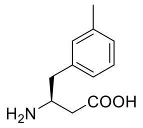
I20	439918-59-9	D-β-Homoproline.HCl	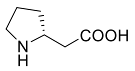
I21	567-35-1	*cis* -L-3-Hydroxyproline	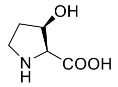
I22	80126-50-7	2-Chloro-D-phenylalanine	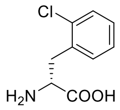
I23	49759-61-7	4-Methyl-D-phenylalanine	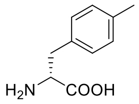
I24	21339-55-9	1-Methyl-L-tryptophan	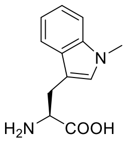
J01	156-86-5	L-Homoarginine	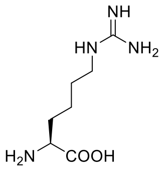
J02	156706-47-7	N^{ω}-Methyl-L-arginine hydrochloride	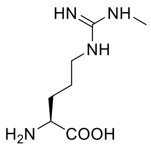
J03	1483-07-4	3-[(Aminocarbony)amino]-L-alanine	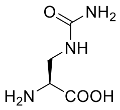
J04	4311-12-0	N^{δ}-Isopropyl-L-glutamine	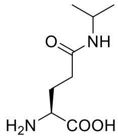
J05	2643-70-1	γ-Glu-4-Abz-OH	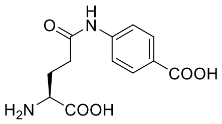

J06	1049734-52-2	(*R*)-γ-(3-Methylbenzyl)-L-proline	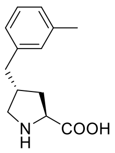
J07	79775-07-8	*O*-*tert*-Butyl-L-*trans*-4-hydroxyproline	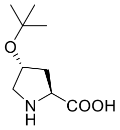
J08	19771-63-2	L-Thiazolidin-2-one-4-carboxylic acid	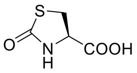
J09	2133-34-8	L-Azetidine-2-carboxylic acid	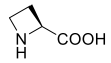
J10	71-00-1	L-Histidine	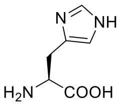
J11	73-22-3	L-tryptophan	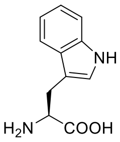
J12	328-38-1	D-Leucine	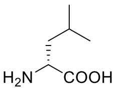
J13	2058-58-4	D-Asparagine hydrate	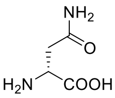
J14	56564-52-4	N^{α}-Methyl-D-phenylalanine hydrochloride	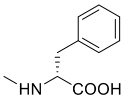
J15	80126-52-9	3-Chloro-D-phenylalanine	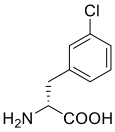
J16	45125-00-6	D-Glutamic acid-γ-tert-butyl ester	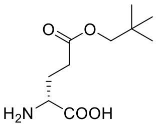
J17	52-67-5	D-Penicillamine	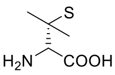
J18	270063-41-7	Pentafluoro-L-β-homophenylalanine hydrochloride	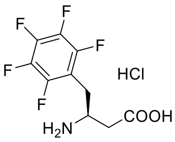
J19	270596-38-8	3-Chloro-L-b-homophenylalanine hydrochloride	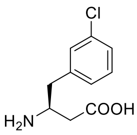

J20	133367-32-5	N^{im}-4-Methyltrityl-L-histidine	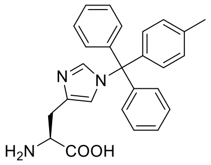
J21	736184-44-4	2-Iodo-D-phenylalanine	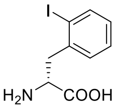
J22	263396-44-7	4-Cyano-D-phenylalanine	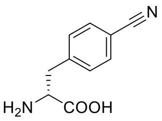
J23	114926-37-3	3-Methyl-L-phenylalanine	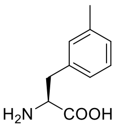
J24	N/A	N/A	N/A
K01	200115-86-2	L-Arg(Pbf)-OH	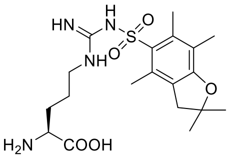
K02	2149-70-4	N^{ω}-Nitro-L-arginine	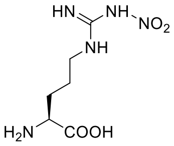
K03	2419-56-9	L-Glutamic acid-γ-tert-butyl ester	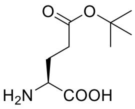
K04	3081-61-6	N^{γ}-Ethyl-L-glutamine	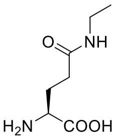
K05	14525-44-1	L-Glutamic acid-γ-(β-naphthylamide)	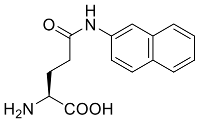
K06	1049755-32-9	(*R*)-γ-Propynyl-L-proline·HCl	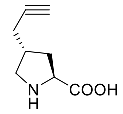
K07	66831-16-1	*O*-Benzyl-L-*trans*-L-4-hydroxyproline hydrochloride	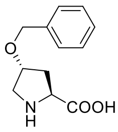

K08	42438-90-4	L-1,2,3,4-Tetrahydronorharman-3-carboxylic acid	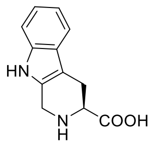
K09	56-40-6	Glycine	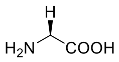
K10	61-90-5	L-Leucine	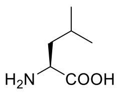
K11	60-18-4	L-Tyrosine	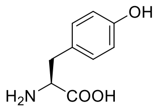
K12	673-06-3	D-Phenylalanine	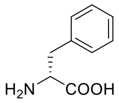
K13	351-50-8	D-Histidine	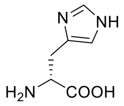
K14	7326-19-4	D-β-Phenyllactic acid	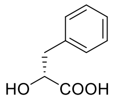
K15	114926-39-5	3-Methyl-D-phenylalanine	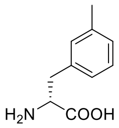
K16	51621-57-9	N^{ε}-Acetyl-D-lysine	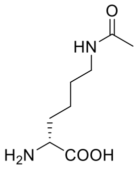
K17	2584-71-6	*cis*-D-4-Hydroxyproline	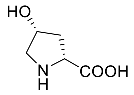
K18	275826-34-1	(*S*)-3-Amino-3-(2-bromophenyl)propionic acid	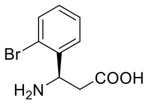
K19	270596-38-8	3-Chloro-L-b-homophenylalanine hydrochloride	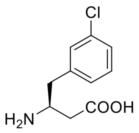
K20	35146-32-8	N^{im}-Trityl-histidine	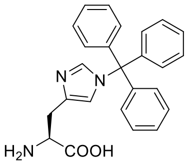

K21	14091-08-8	4-Chloro-D-phenylalanine	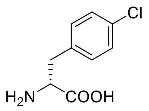
K22	122839-51-4	2-Fluoro-D-phenylalanine	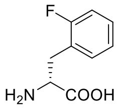
K23	170642-31-6	D-2-Methoxyphenylalanine	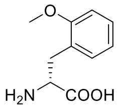
K24	N/A	N/A	N/A
L01	4125-79-5	N^{ω}, N^{ω}-Di-Z-L-arginine	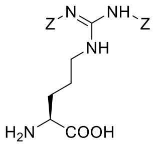
L02	4353-32-6	N^{ω}-(4-Toluenesulfonyl)-L-arginine	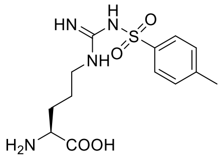
L03	5963-60-0	L-Glutamic acid-γ-anilide	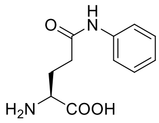
L04	5963-60-0	L-Glutamic acid-γ-anilide	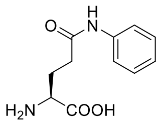
L05	336182-11-7	L-β-Homohydroxyproline hydrochloride	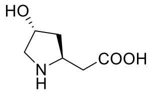
L06	1049743-68-1	(*R*)-γ-(4-Trifluoromethylbenzyl)-L-proline	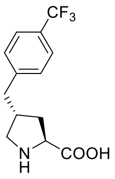
L07	51-35-4	*trans*-L-4-Hydroxyproline	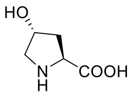
L08	152286-30-1	7-hydroxy-D-Tic-OH	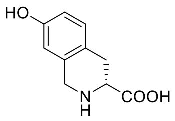
L09	56-41-7	L-Alanine	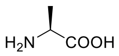

L10	63-68-3	L-Methionine	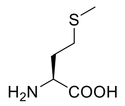
L11	72-18-4	L-Valine	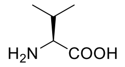
L12	312-84-5	D-Serine	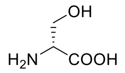
L13	632-20-2	D-Threonine	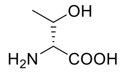
L14	331763-65-6	3-Fluoro-D-β-homophenylalanine hydrochloride	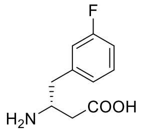
L15	14464-67-6	3-Trifluoromethyl-D-phenylalanine	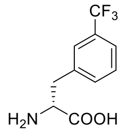
L16	31202-69-4	N^{ε}-Boc-D-lysine	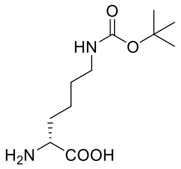
L17	1723-00-8	D-Homoproline	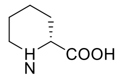
L18	151911-32-9	(*S*)-3-Amino-3-(2-fluorophenyl)propionic acid	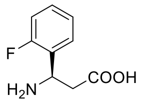
L19	270065-91-3	(2-Thienyl)-L-β-homoalanine hydrochloride	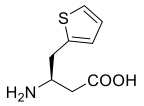
L20	84888-34-6	*S*-9-Fluorenylmethyl-L-cysteine hydrochloride	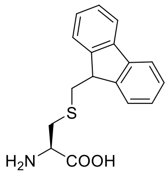
L21	18125-46-7	4-Fluoro-D-phenylalanine	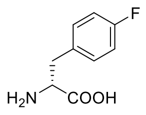
L22	62561-75-5	4-Iodo-D-phenylalanine	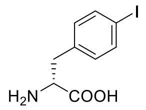

L23	145306-65-6	D-3-Methoxyphenylalanine	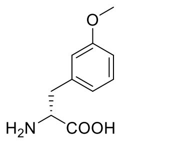
L24	N/A	N/A	N/A
M01	80745-10-4	*N*$^{\omega}$-(4-Methoxy-2,3,6-trimethylbenzenesulfonyl)-L-arginine	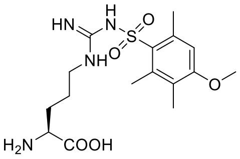
M02	2177-63-1	L-Aspartic acid β-benzyl ester	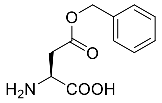
M03	1119-33-1	L-Glutamic acid-γ-ethyl ester	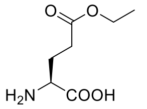
M04	67953-08-6	L-Glutamic acid-γ-(p-nitroanilide) hydrochloride	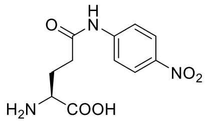
M05	213475-47-9	L-2,2-Dimethyl-thiaproline hydrochloride	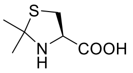
M06	1049740-11-5	(*R*)-γ-(3-Nitrobenzyl)-L-proline·HCl	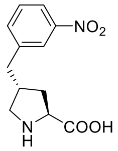
M07	4043-88-3	3,4-Dehydro-L-proline	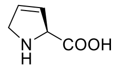
M08	618-27-9	*cis*-L-4-Hydroxyproline	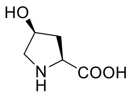
M09	74-79-3	L-Arginine	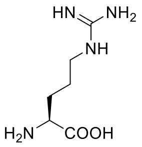
M10	63-91-2	L-Phenylalanine	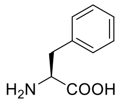
M11	52-90-4	L-Cysteine	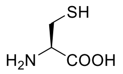
M12	556-02-5	D-Tyrosine	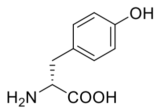

M13	348-67-4	D-Methionine	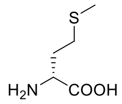
M14	263396-43-6	3-Cyano-D-phenylalanine	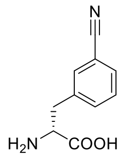
M15	20312-37-2	D-α-Hydroxyisocaproic acid	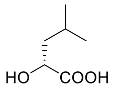
M16	201014-19-9	*N*[ε]-2-Chloro-Z-D-lysine	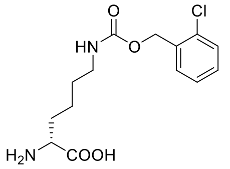
M17	45521-09-3	D-Thiaproline	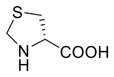
M18	270596-50-4	3-Fluoro-L-β-homophenylalanine hydrochloride	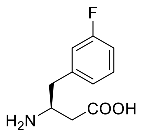
M19	N/A	N/A	N/A
M20	144317-20-4	N[γ]-4-Methyltrityl-L-asparagine	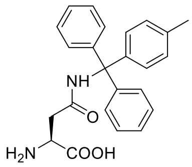
M21	56613-61-7	4-Nitro-D-phenylalanine monohydrate	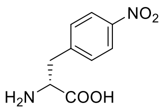
M22	130930-49-3	H-D-Phe(2-trifluoromethyl)-OH	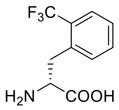
M23	80126-54-1	2-Methyl-D-phenylalanine	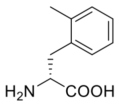
M24	N/A	N/A	N/A
N01	121080-96-4	D-Homocitrulline	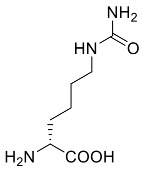

N02	112259-66-2	L-Aspartic acid-β-cyclohexyl ester	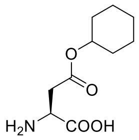
N03	2922-83-0	L-Kynurenine	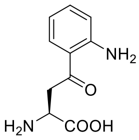
N04	1499-55-4	L-Glutamic acid-γ-methyl ester	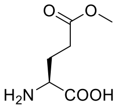
N05	53912-85-9	L-β-Homoproline hydrochloride	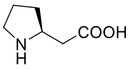
N06	1049745-26-7	(*R*)-γ-(4-Biphenylmethyl)-L-proline·HCl	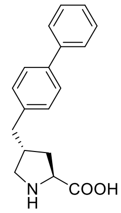
N07	128502-56-7	7-hydroxy-L-Tic-OH	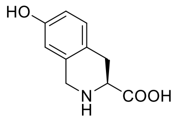
N08	79815-20-6	L-Indoline-2-carboxylic acid	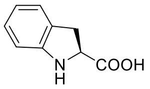
N09	5794-13-8	L-Asparagine monohydrate	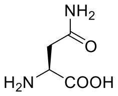
N10	147-85-3	L-Proline	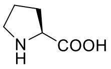
N11	56-87-1	L-Lysine	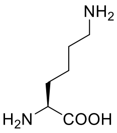
N12	640-68-6	D-Valine	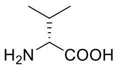
N13	319-78-8	D-Isoleucine	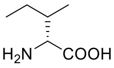
N14	110117-84-5	3-Fluoro-D-phenylalanine	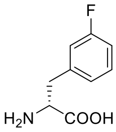

N15	17407-56-6	D-α-Hydroxyisovaleric acid	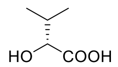
N16	274260-42-3	N^{ε}-Allyloxycarbonyl-D-lysine	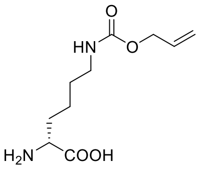
N17	201290-11-1	N^{in}-Boc-D-tryptophan	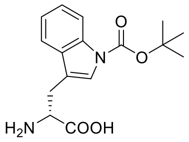
N18	719995-40-1	(*S*)-3-Amino-3-(3-trifluoromethylphenyl)propionic acid	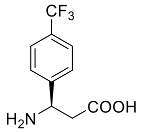
N19	269398-82-5	3-Methyl-D-β-homophenylalanine hydrochloride	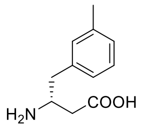
N20	84624-28-2	N^{ε}-Fmoc-L-lysine	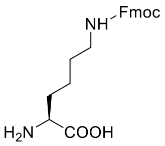
N21	263396-41-4	2-Cyano-D-phenylalanine	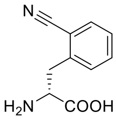
N22	39878-65-4	*O*-Methyl-D-tyrosine	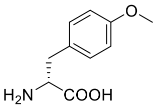
N23	267225-27-4	2-Bromo-D-phenylalanine	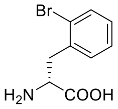
N24	N/A	N/A	N/A
O01	112160-37-9	N^{ω}-(2,2,5,7,8-Pentamethylchroman-6-sulfonyl)-L-arginine	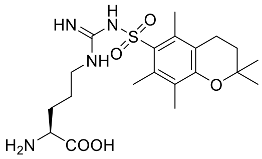
O02	16856-13-6	L-Aspartic acid-β-methyl ester hydrochloride	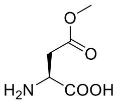
O03	1118-90-7	L-α-Aminoadipic acid	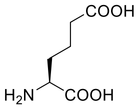

O04	122864-94-2	L-Glutamic acid 5-(4-nitroanilide) monohydrate	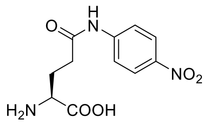
O05	3105-95-1	L-Homoproline	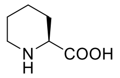
O06	1049733-88-1	(*R*)-γ-(4-Chlorobenzyl)-L-proline	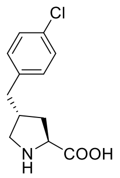
O07	74163-81-8	(3*S*)-1,2,3,4-Tetrahydroisoquinoline-3-carboxylic acid	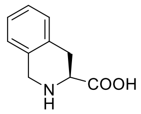
O08	34592-47-7	L-Thiaproline	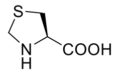
O09	56-84-8	L-Aspartic acid	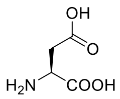
O10	73-32-5	L-Isoleucine	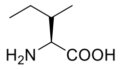
O11	338-69-2	D-Alanine	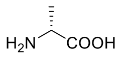
O12	344-25-2	D-Proline	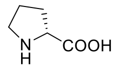
O13	923-27-3	D-Lysine	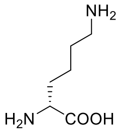
O14	99295-78-0	3-Bromo-D-phenylalanine	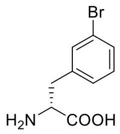
O15	66036-77-9	N^{ω}-Nitro-D-arginine	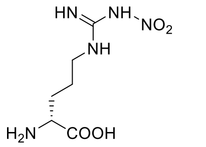
O16	269398-95-0	D-β-HomoAla(3-benzothienyl)-OH	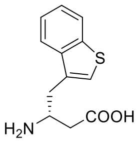

O17	367453-01-8	N^{in}-Formyl-D-tryptophan	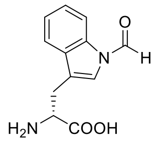
O18	270065-85-5	3-Cyano-L-β-homophenylalanine	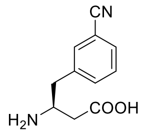
O19	331763-55-4	3-Chloro-D-β-homophenylalanine	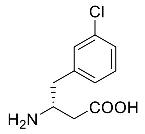
O20	2799-07-7	*S*-Trityl-L-cysteine	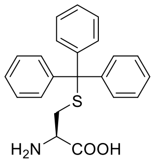
O21	114872-99-0	ρ-Trifluoromethyl-D-phenylalanine	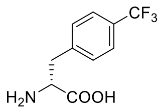
O22	126257-07-6	4-Amino-D-phenylalanine	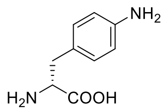
O23	N/A	N/A	N/A
O24	N/A	N/A	N/A
P01	191869-60-0	N^{ω}-(2,2,5,7,8-Pentamethylchroman-6-sulfonyl)-D-arginine	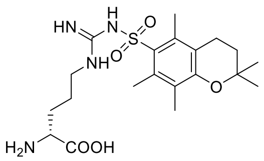
P02	3057-74-7	L-Aspartic acid-β-tert-butyl ester	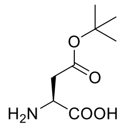
P03	1676-73-9	L-Glutamic acid-γ-benzyl ester	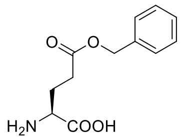
P04	1119-33-1	L-Glutamic acid-γ-ethyl ester	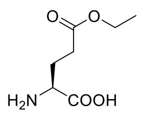
P05	2507-61-1	*trans*-4-Fluoro-L-proline	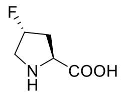

P06	1049734-62-4	(*R*)-γ-(4-Methylbenzyl)-L-proline	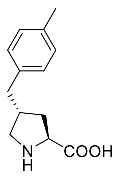
P07	59981-63-4	L-4,5,6,7-Tetrahydro-1H-imidazo[4,5-c]pyridine-6-carboxylic acid	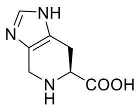
P08	892128-58-4	(4*S*)-Azido-L-Proline	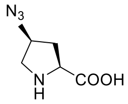
P09	56-85-9	L-Glutamine	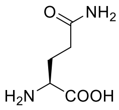
P10	56-45-1	L-Serine	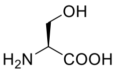
P11	6893-26-1	D-Glutamic acid	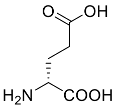
P12	1783-96-6	D-Aspartic acid	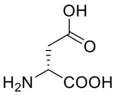
P13	921-01-7	D-Cysteine	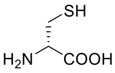
P14	269726-82-1	3-Cyano-D-β-homophenylalanine	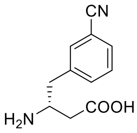
P15	97233-92-6	N^{ω}-(4-Toluenesulfonyl)-D-arginine	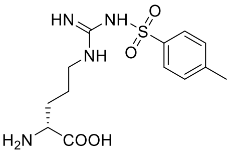
P16	111139-55-0	3-Benzothienyl-D-alanine	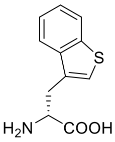
P17	40332-58-9	Pentafluoro-D-phenylalanine	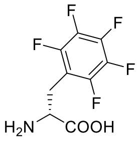
P18	270065-76-4	3-Trifluoromethyl-L-β-homophenylalanine	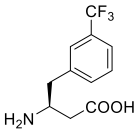

P19	269726-73-0	3-Trifluoromethyl-D-β-homophenylalanine	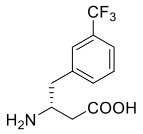
P20	132388-58-0	N^{γ}-Trityl-L-asparagine	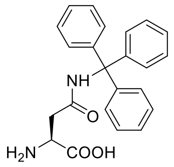
P21	62561-74-4	4-Bromo-D-phenylalanine	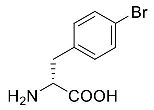
P22	274262-82-7	4-tert-Butyl-D-phenylalanine	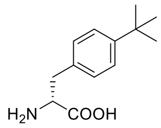
P23	110117-83-4	1-Methyl-D-tryptophan	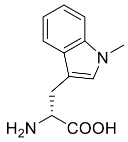
P24	5162-90-3	3-(2-Oxo-1,2-dihydro-4-quinolinyl)alanine hydrochloride monohydrate	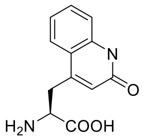

*N/A designates an empty well

5. Table S2. Arrangement of ncAAs Library

	1	2	3	4	5	6	7	8	9	10	11	12	13	14	15	16	17	18	19	20	21	22	23	24
P	P1	P2	P3	P4	P5	P6	P7	P8	P9	P10	P11	P12	P13	P14	P15	P16	P17	P18	P19	P20	P21	P22	P23	P24
O	O1	O2	O3	O4	O5	O6	O7	O8	O9	O10	O11	O12	O13	O14	O15	O16	O17	O18	O19	O20	O21	O22	O23	O24
N	N1	N2	N3	N4	N5	N6	N7	N8	N9	N10	N11	N12	N13	N14	N15	N16	N17	N18	N19	N20	N21	N22	N23	N24
M	M1	M2	M3	M4	M5	M6	M7	M8	GMML	M10	M11	M12	M13	M14	M15	M16	M17	M18	M19	M20	M21	M22	M23	M24
L	L1	L2	L3	L4	L5	L6	L7	L8	L9	L10	L11	L12	L13	L14	L15	L16	L17	L18	L19	L20	L21	L22	L23	L24
K	K1	K2	K3	K4	K5	K6	K7	K8	K9	K10	K11	K12	K13	K14	K15	K16	K17	K18	K19	K20	K21	K22	K23	K24
J	J1	J2	J3	J4	J5	J6	J7	J8	J9	J10	J11	J12	J13	J14	J15	J16	J17	J18	J19	J20	J21	J22	J23	J24
I	I1	I2	I3	I4	I5	I6	I7	I8	I9	I10	I11	I12	I13	I14	I15	I16	I17	I18	I19	I20	I21	I22	I23	I24
H	H1	H2	H3	H4	H5	H6	H7	H8	H9	H10	H11	H12	H13	H14	H15	H16	H17	H18	H19	H20	H21	H22	H23	H24
G	G1	G2	G3	G4	G5	G6	G7	G8	G9	G10	G11	G12	G13	G14	G15	G16	G17	G18	G19	G20	G21	G22	G23	G24
F	F1	F2	F3	F4	F5	F6	F7	F8	F9	F10	F11	F12	F13	F14	F15	F16	F17	F18	F19	F20	F21	F22	F23	F24
E	E1	E2	E3	E4	E5	E6	E7	E8	E9	E10	E11	E12	E13	E14	E15	E16	E17	E18	E19	E20	E21	E22	E23	E24
D	D1	D2	D3	D4	D5	D6	D7	D8	D9	D10	D11	D12	D13	D14	D15	D16	D17	D18	D19	D20	D21	D22	D23	D24
C	C1	C2	C3	C4	C5	C6	C7	C8	C9	C10	C11	C12	C13	C14	C15	C16	C17	C18	C19	C20	C21	C22	C23	C24
B	B1	B2	B3	B4	B5	B6	B7	B8	B9	B10	B11	B12	B13	B14	B15	B16	B17	B18	B19	B20	B21	B22	B23	B24
A	A1	A2	A3	A4	A5	A6	A7	A8	A9	A10	A11	A12	A13	A14	A15	A16	A17	A18	A19	A20	A21	A22	A23	A24

*The wells labelled in red are used as controls (without ncAAs and IPTG); the wells without ncAAs but containing IPTG are labelled in green.
